# Fullerene-Functionalized Halogen-Bonding Heteroditopic Hosts for Ion-Pair Recognition

**DOI:** 10.1021/jacs.3c07774

**Published:** 2023-12-07

**Authors:** Krzysztof M. Bąk, Igor Marques, Heike Kuhn, Kirsten E. Christensen, Vítor Félix, Paul D. Beer

**Affiliations:** † Chemistry Research Laboratory, Department of Chemistry, University of Oxford, Oxford OX1 3TA, U.K.; ‡ CICECO - Aveiro Institute of Materials, Department of Chemistry, University of Aveiro, 3810-193 Aveiro, Portugal

## Abstract

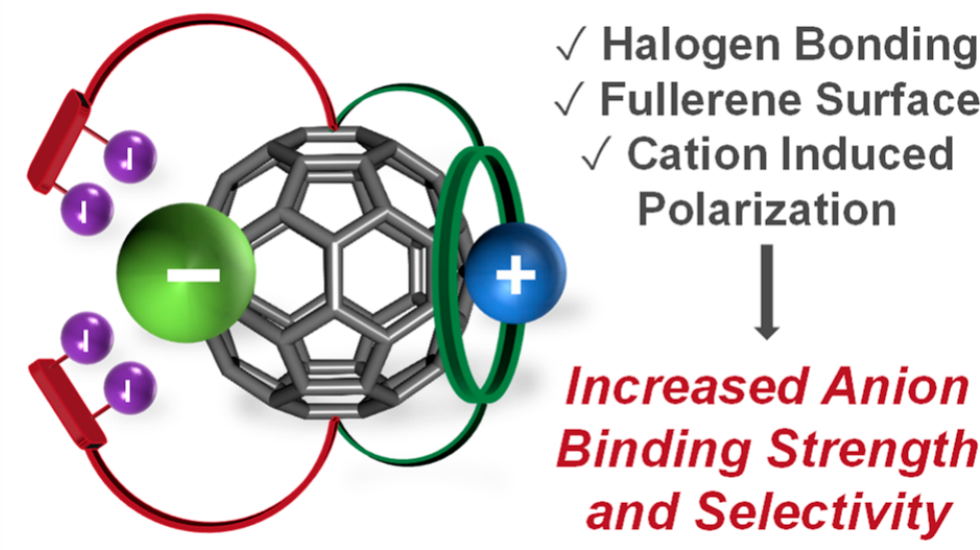

Despite their hydrophobic surfaces with localized π-holes and rigid well-defined architectures providing a scaffold for preorganizing binding motifs, fullerenes remain unexplored as potential supramolecular host platforms for the recognition of anions. Herein, we present the first example of the rational design, synthesis, and unique recognition properties of novel fullerene-functionalized halogen-bonding (XB) heteroditopic ion-pair receptors containing cation and anion binding domains spatially separated by C_60_. Fullerene spatial separation of the XB donors and the crown ether complexed potassium cation resulted in a rare example of an artificial receptor containing two anion binding sites with opposing preferences for hard and soft halides. Importantly, the incorporation of the C_60_ motif into the heteroditopic receptor structure has a significant effect on the halide binding selectivity, which is further amplified upon K^+^ cation binding. The potassium cation complexed fullerene-based receptors exhibit enhanced selectivity for the soft polarizable iodide ion which is assisted by the C_60_ scaffold preorganizing the potent XB-based binding domains, anion−π interactions, and the exceptional polarizability of the fullerene moiety, as evidenced from DFT calculations. These observations serve to highlight the unique properties of fullerene surfaces for proximal charged guest binding with potential applications in construction of selective molecular sensors and modulating the properties of solar cell devices.

## Introduction

Fullerenes are unique molecules with a large spherical surface, strong electron acceptor properties, high electric polarizability, and curved π-electron systems capable of forming noncovalent interactions with electron-rich molecules.^1−13^ As a result, they have been incorporated in numerous functional molecular assemblies and supramolecular arrays with a wide variety of applications in photochemistry, medicinal chemistry, and organic electronics.^14−26^ Although molecular electrostatic potential (MEP) surfaces of simple fullerenes are positive, surprisingly, their interaction with anions has been largely overlooked.^6,27^ Only recently, Matile and co-workers demonstrated remarkable examples of the stabilization of anionic transition states in anion−π catalysis on a fullerene surface,^28−30^ while Lei and co-workers reported facilitated charge transfer in solid-state aggregates of self-*n*-doped fullerene ammonium iodide, which is believed to be a result of iodide–C_60_ interactions.^31^ However, thus far fullerene surfaces have not been exploited as potential supramolecular host platforms for the recognition of simple anions (e.g., halides) in molecular receptor structural design. Nevertheless, it is worth noting that an open-cage fullerene was demonstrated as a molecular container for F^–^, Cl^–^, Br^–^, and I^–^.^32^

**Figure 1:**
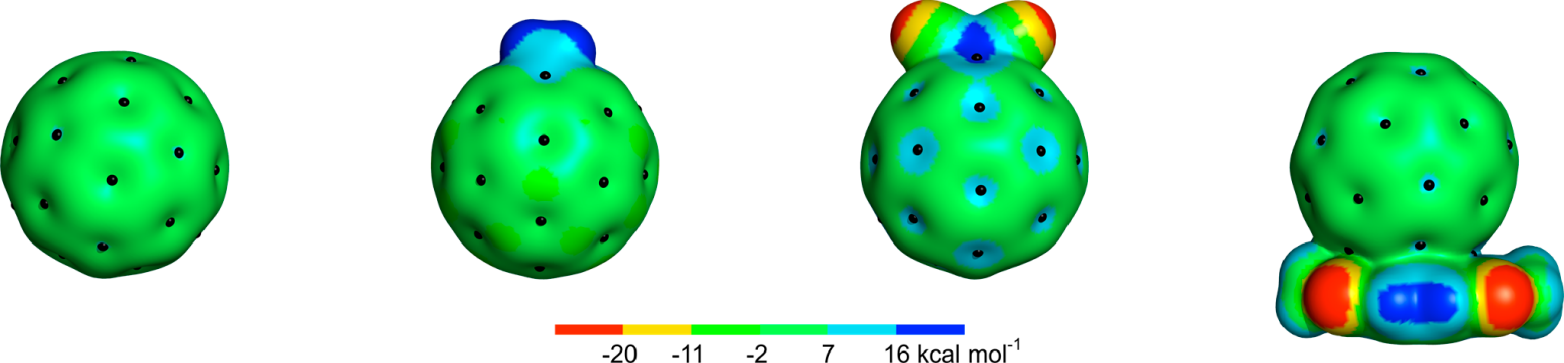
Molecular electrostatic potential (MEP) surfaces of C_60_ (left), C_60_ functionalized with methylene (center left) or two cyano electron-withdrawing groups (center right), and C_60_ in association with naphthalene diimide (right). The MEP surfaces are rendered at the 0.001 electrons Bohr^–3^ contour and the π-holes on the surface of C_60_ are identified as black dots.

The MEP surface of C_60_ reveals highly localized areas of positive potential, π-holes (Figure 1), which can be presumably used in anion recognition. Anion−π interactions are widely recognized and frequently exploited in the design of selective anion binding receptors.^33−36^ Strong attraction between an anion and a π-system can be achieved by electron-withdrawing substituents that further polarize the molecule and lead to a positive quadrupole moment along the axis perpendicular to the π-system, increasing the depth of a π-hole.^33^ Interestingly, fullerenes are known for their remarkable polarizability^37^ which, in principle, may enable a significant enhancement of anion−π interactions by exposure to an external electric field, produced for example by a proximate anion (so-called dynamic contribution) or cation. Moreover, the well-defined bulky architecture of fullerenes provides a potential scaffold for preorganization of binding motifs and a hydrophobic shield, which can create a microenvironment that excludes solvent molecules and enhances strength of noncovalent interactions.^38,39^ Due to these features, C_60_ constitutes an exceptional and unexplored platform for the design and construction of heteroditopic ion-pair receptors with increased affinity and selectivity. The positive cooperativity associated with the simultaneous proximal binding of oppositely charged species has been crucial in augmenting the ion-pair binding properties of heteroditopic receptors relative to their monotopic receptor counterparts. As such, heteroditopic receptors have been increasingly employed in a myriad of applications including salt extraction and solubilization,^40,41^ membrane transport,^42,43^ and biological zwitterion binding.^44,45^

**Figure 2:**
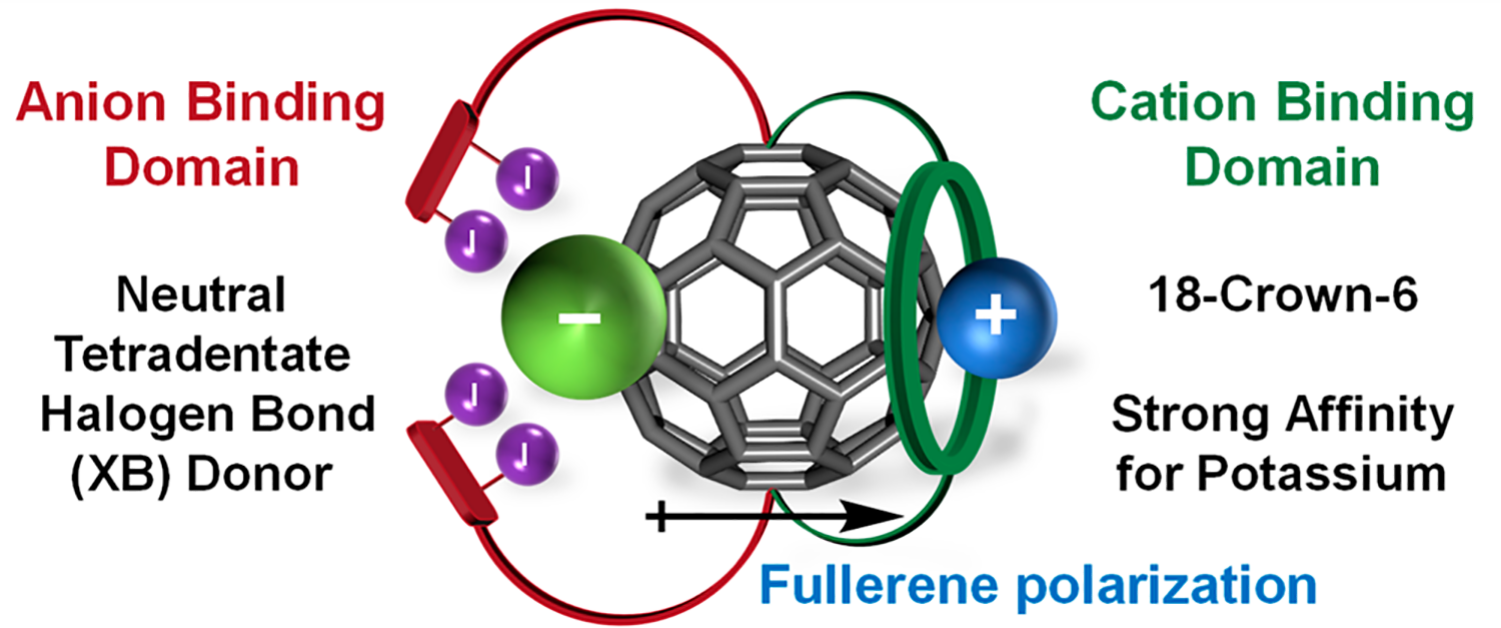
Cartoon representation of a tetradentate XB fullerene-functionalized heteroditopic host for ion-pair recognition. Purple spheres represent iodine halogen bond donors.

The vast majority of reported heteroditopic receptors contain well-established recognition motifs such as crown ethers for cation recognition and hydrogen bond donors for anion complexation.^46−48^ In recent years, however, halogen bonding (XB), an interaction between a Lewis base and the σ-hole of an electron-deficient halogen atom, has emerged as a powerful addition to the anion supramolecular host–guest chemistry toolbox, due to its stringent linearity, comparable binding strength to hydrogen bonding (HB), and distinctive selectivity.^49−52^ Herein, we describe for the first time the rational design, synthesis, and unique recognition properties of novel fullerene-functionalized halogen-bonding heteroditopic ion-pair receptors containing cation and anion binding domains spatially separated by C_60_ (Figure 2).

## Results and Discussion

### Synthesis of Fullerene Heteroditopic Ion-Pair Receptors

**Figure 3:**
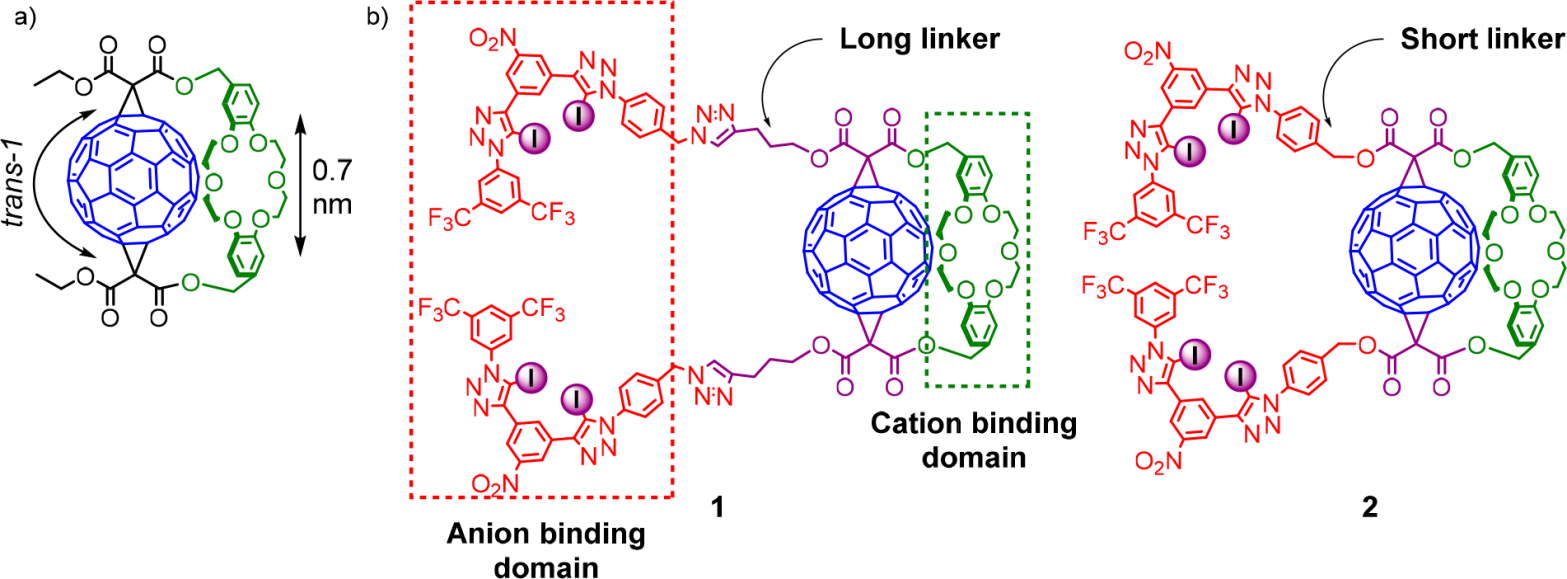
(a) C_60_-dibenzo-18-crown-6 adduct obtained by Echegoyen, Pretsch, Diederich, and co-workers. (b) Heteroditopic ion-pair receptors **1** and **2** with different linkers separating anion binding domains from the C_60_ surface.

Interest in crown ether–fullerene adduct materials has been stimulated primarily by their photophysical, electrochemical, and superconducting properties.^53^ In particular, Echegoyen, Pretsch, Diederich, and co-workers obtained the C_60_-dibenzo-18-crown-6 (DB18C6) adduct in a highly regioselective double cyclopropanation (Bingel addition) taking place exclusively in the *trans-1* positions on the opposite poles of C_60_ (Figure 3a).^54,55^ Potassium cation crown ether binding in the proximity of the fullerene surface was shown to elicit significant perturbations of the fullerene host’s reduction potentials, proving that alkali metal cation complexation can alter the physicochemical properties of C_60_. We hypothesized that a complexed cation could further polarize the fullerene surface, resulting in anion binding enhancement on the opposite size of the molecule (Figure 2). Therefore, we adapted the regioselective DB18C6 double Bingel fullerene addition for the synthesis of heteroditopic ion-pair receptors **1** and **2** containing neutral acyclic XB donors based on 1,3-bis(iodotriazole)nitroaryl motifs in the anion binding domains,^56^ spatially separated from the polarizable fullerene surface by linkers of different lengths (Figure 3b).

**Scheme 1:**
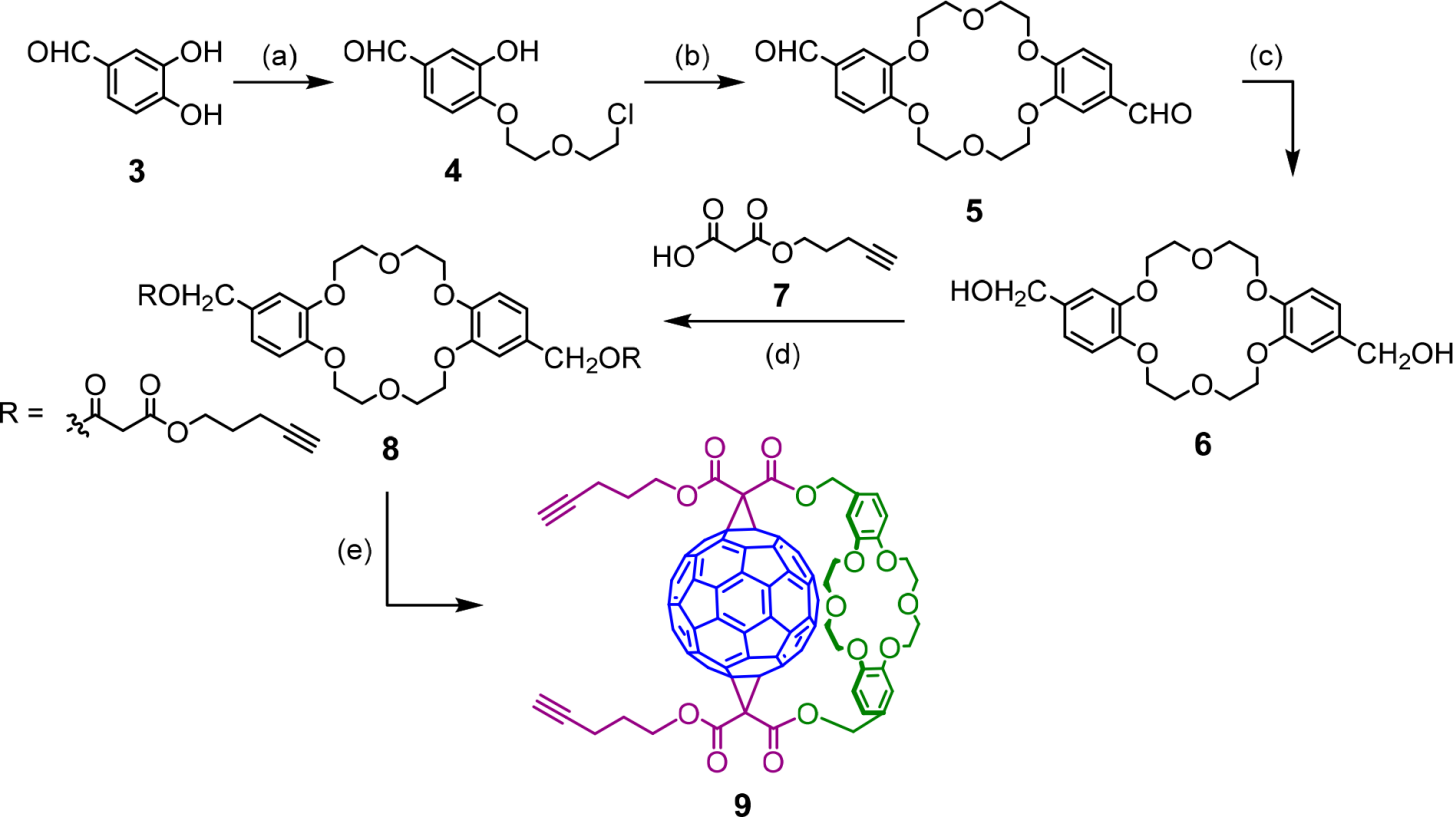
Synthesis of C_60_-DB18C6 Adduct **9** Reagents and conditions: (a) (ClCH_2_)_2_O, K_2_CO_3_, DMF, 80 °C, 36 h, 21%; (b) K_2_CO_3_, DMF, 80 °C, 24 h, 31%; (c) NaBH_4_, THF/MeOH 9:1, 0 °C, 2 h, 62%; (d) EDC·HCl, DMAP, KPF_6_, CH_2_Cl_2_/CH_3_CN 4:1, 0 °C, 48 h, 76%; (e) C_60_, I_2_, DBU, KPF_6_, toluene, RT, 6 h, 25%.

**Scheme 2:**
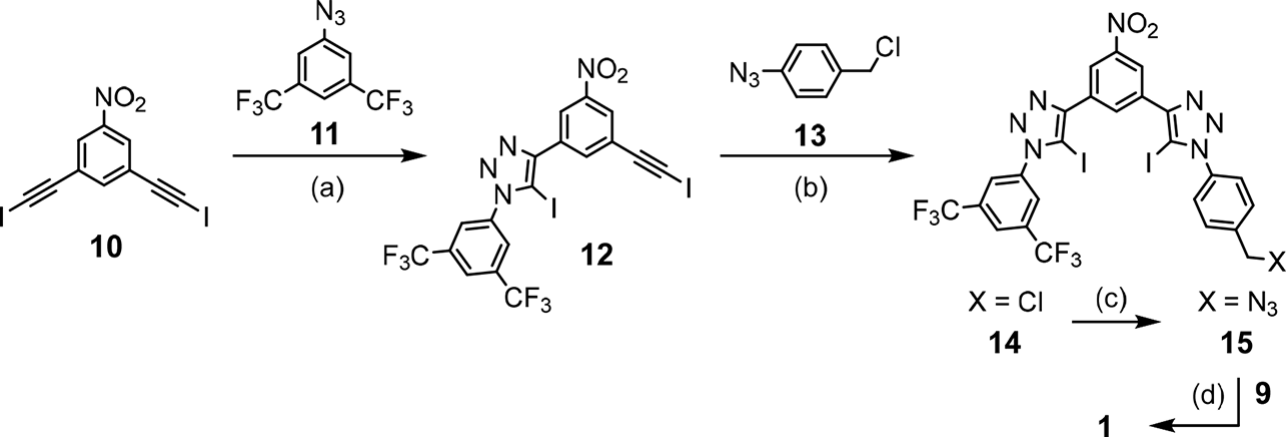
Synthesis of Anion Binding Domain and Receptor **1** Reagents and conditions: (a) [Cu(CH_3_CN)_4_]PF_6_, TBTA, CH_2_Cl_2_, RT, 16 h, 65%; (b) [Cu(CH_3_CN)_4_]PF_6_, TBTA, CH_2_Cl_2_, RT, 16 h, 72%; (c) NaN_3_, DMF, RT, 24 h, 60%; (d) [Cu(CH_3_CN)_4_]PF_6_, TBTA, CH_2_Cl_2_, RT, 48 h, 36%.

**Figure 4:**
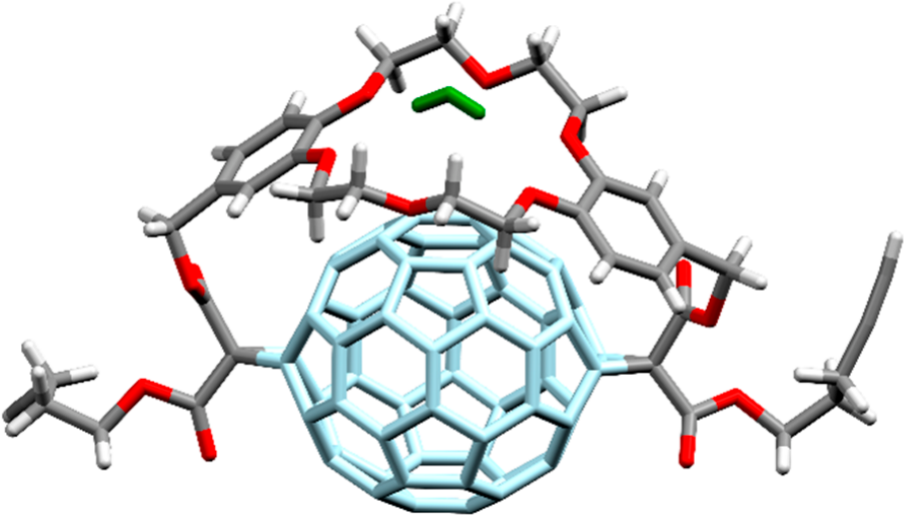
Solid-state structure of C_60_-DB18C6 adduct **9** with a water molecule (green color) hydrogen bonded to a crown ether.

The separate appropriately functionalized crown ether–fullerene cation and halogen-bonding anion binding domain synthons were prepared according to Schemes 1 and 2. The synthesis of the bis-alkyne appended crown ether–fullerene synthon **9** was achieved via modification of the regioselective procedure reported by Diederich and co-workers (Scheme 1).^54^ 3,4-Dihydroxybenzaldehyde **3** was alkylated with an excess of bis(2-chloroethyl) ether to obtain **4** (21%), which could be readily separated from the other regioisomer. Macrocyclization of **4** in the presence of the K^+^ template afforded the poorly soluble *trans*-dialdehyde of DB18C6 **5** (31%), which upon reduction using NaBH_4_ gave the diol **6** (62%). Monomalonate **7** was prepared either by treating 4-pentyn-1-ol with Meldrum’s acid (68%) or via an alternative approach involving selective monohydrolysis of a symmetric malonic ester (see the Supporting Information for details). Diol **6** was coupled with excess **7** using EDC to obtain bis-malonate ester crown ether **8** (76%). Bingel reaction of **8** with C_60_ in the presence of K^+^ and I_2_ led exclusively to doubly substituted fullerene adduct **9** (25%). The ^1^H NMR and ^13^C NMR spectra of **9** (Figures S5 and S6)were in agreement with the *trans*-1 addition pattern (*C*_2_ symmetry), which was later unambiguously confirmed by single crystal X-ray diffraction structural analysis (Figure 4).^57^ Solid-state analysis also revealed that the DB18C6 ester groups of the cyclopropane rings are situated on the same side of the fullerene (out–out isomer). Interestingly, rotation of the crown ether moiety is significantly limited, causing planar chirality and splitting of the benzylic and ether CH_2_ signals in the ^1^H NMR spectrum (see Figure S5).

The synthesis of the azide-functionalized XB anion binding domain **15** required desymmetrization of bis-iodoalkyne building block **10** (Scheme 2). An excess of **10** was treated with azide **11** in the presence of [Cu(MeCN)_4_]PF_6_ and Cu(I) stabilizing ligand TBTA to obtain mono-iodotriazole **12** (65%). It was then reacted with azide **13** to obtain asymmetric bis-iodotriazole **14** (72%), which could be readily transformed into azide **15** (60%). In the final step, the anion binding moiety was doubly “clicked” with bis-alkyne crown ether fullerene adduct **9** to obtain ion-pair receptor **1** following purification by flash and size-exclusion chromatography (36%).

**Scheme 3:**
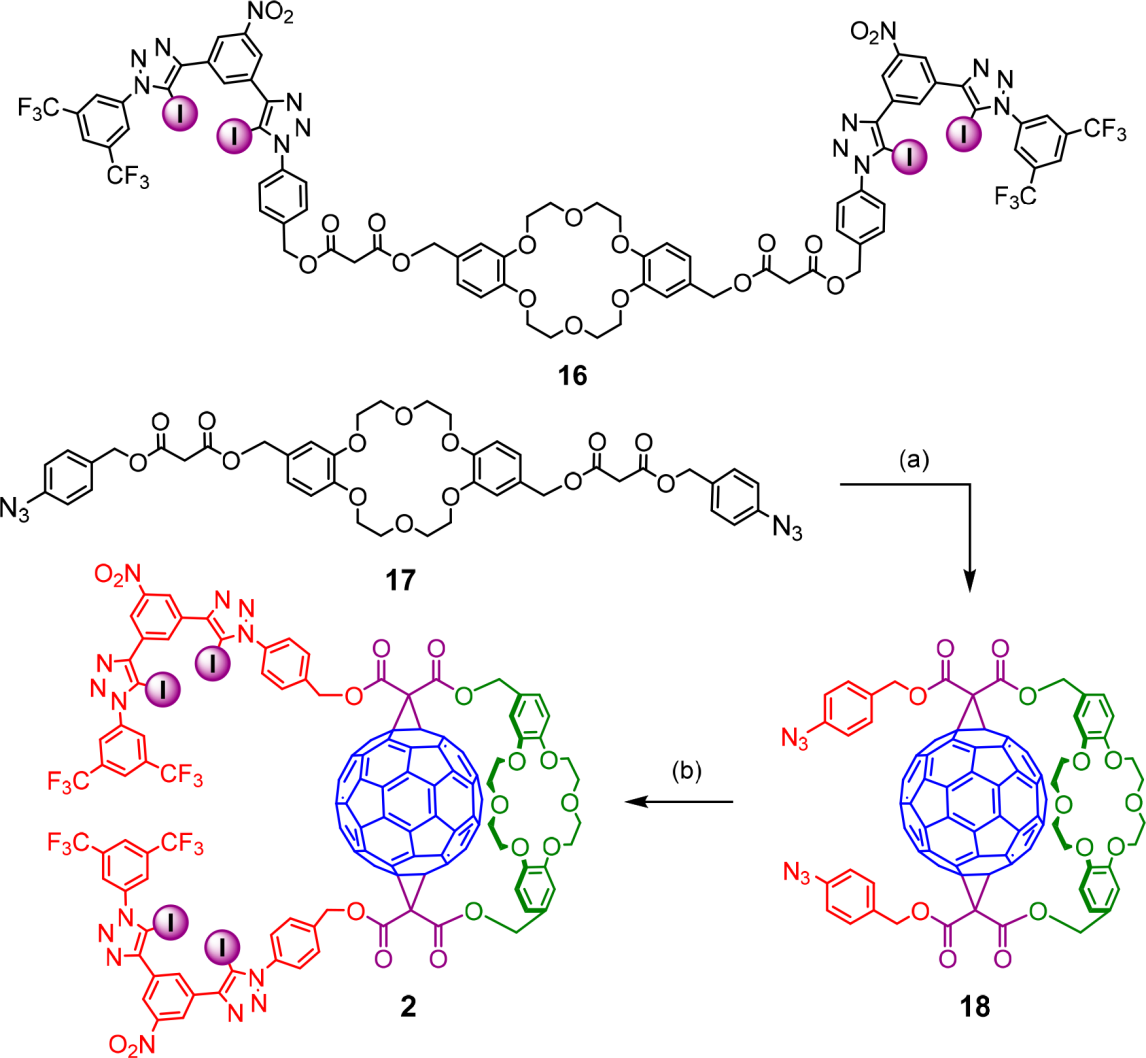
Receptor **16** and the Synthesis of Receptor **2** Reagents and conditions: (a) C_60_, I_2_, DBU, KPF_6_, toluene, RT, 1 h; (b) **12**, [Cu(CH_3_CN)_4_]PF_6_, TBTA, CH_2_Cl_2_, RT, 18 h, 14%.

The synthesis of receptor **2** with a shorter linker between the anion binding domain and the fullerene surface was initially attempted in an alternative approach with a Bingel reaction conducted on precursor **16** (Scheme 3). Unfortunately, this resulted in a complex mixture of products, suggesting that the anion binding motif is not compatible with the conditions of the cyclopropanation reaction. An alternative route involving Cu(I)-catalyzed azide–alkyne cycloaddition (CuAAC) reaction between alkyne **12** and DB18C6-fullerene bis-azide **18** proved successful (Scheme 3). Fullerene azides are known to be highly unstable due to possible reactions at the fullerene surface.^58−60^ However, a Bingel reaction of short duration time between **17** and C_60_, followed by rapid chromatographic purification afforded **18**, which was used immediately in the next step to obtain final receptor **2** (14%).

### Anion and Ion-Pair Binding Studies

**Figure 5:**
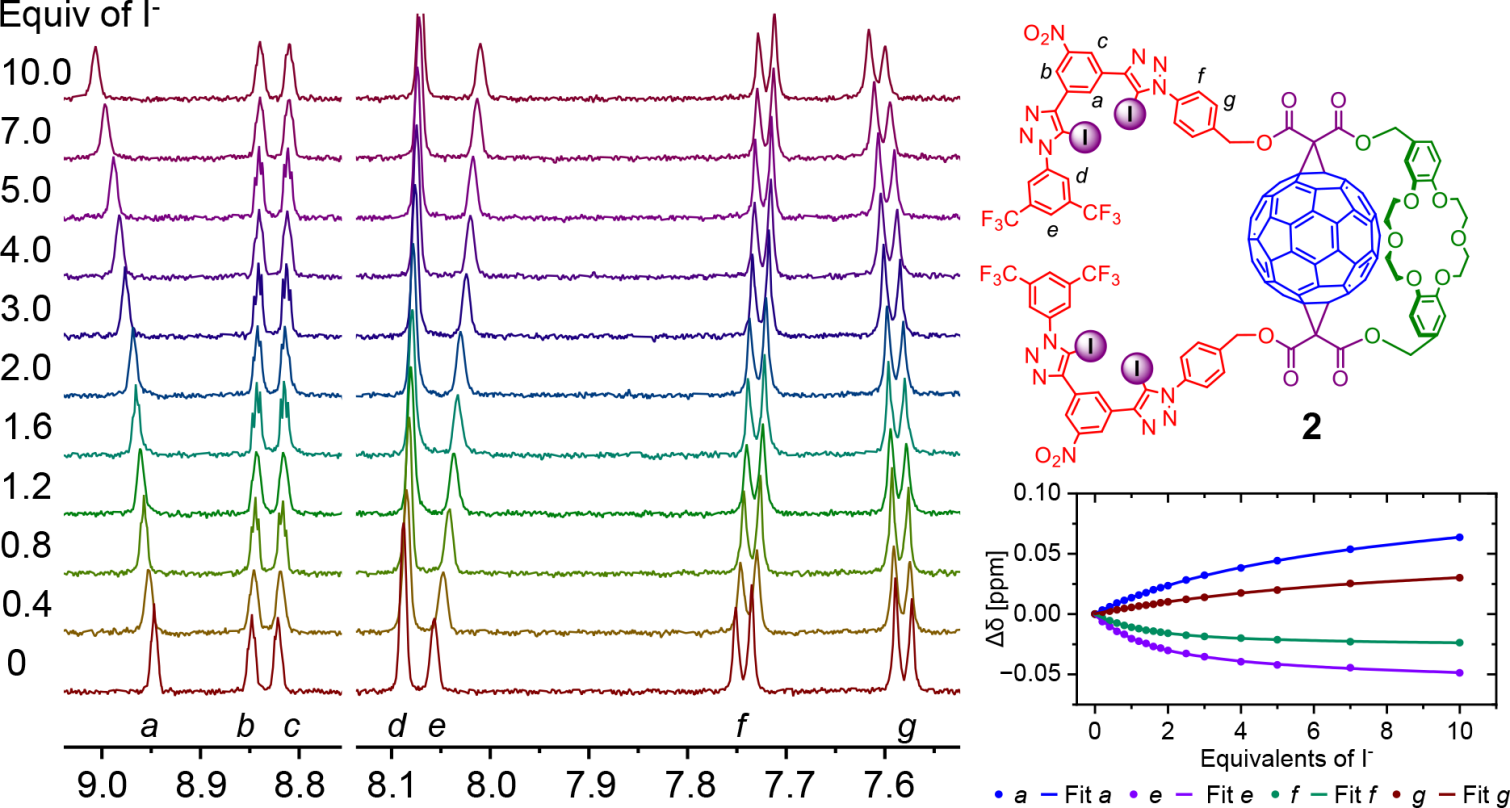
Truncated ^1^H NMR spectra of receptor **2** in 3:1 CDCl_3_:CD_3_CN with an increasing amount of TBAI. Corresponding binding isotherms were used for determining values of binding constant.

The anion binding properties of fullerene containing heteroditopic receptors **1** and **2** were investigated by ^1^H NMR titration experiments in 3:1 CDCl_3_:CD_3_CN. Addition of TBA halides (Cl^–^, Br^–^, I^–^) to solutions of the free receptors **1** or **2** caused significant shifts of the XB anion binding domain proton signals, and no changes (Δδ < 0.01 ppm) of the crown ether cation binding protons were observed. Such a behavior strongly indicates that the ditopic binding domains of receptors **1** and **2** are electronically and spatially well-separated from each other. Notably, the respective receptor’s internal nitroaryl proton (*a*) shifted downfield, which is indicative of halide binding in a cavity formed by the iodotriazole XB donors (Figure 5). Bindfit analysis of the titration isotherm data revealed that 1:1 and 1:2 stoichiometric host–guest complexes are formed (Table 1).^74,75^ In the 1:1 complex, the halide anion is most likely bound by both bidentate XB motifs, contributing up to four halogen bond donors. Upon addition of excess halide anion, each appended XB bidentate recognition site binds an individual anion with two halogen bond donors to form a 1:2 stoichiometric host–guest complex. Unsurprisingly then, *K*_1:1_ association constant values are more than an order of magnitude larger than those of *K*_1:2_. Receptor **1** binds halides more strongly than **2** with a selectivity trend of Br^–^ > I^–^ > Cl^–^. The enhanced halide anion binding by **1** may be attributed to the longer, flexible linker providing the conformational freedom to facilitate the formation of stronger XB–halide anion interactions in the 1:1 complex. Control receptor **16**, which does not contain fullerene, exhibits a halide selectivity trend mirroring that of **1**, however with lower *K*_1:1_ values. Interestingly, receptor **2** exhibits a unique, however modest, preference for I^–^ over Br^–^ and Cl^–^ which may be a result of a shorter distance between the XB binding units and the fullerene surface. The combination of the C_60_ fullerene scaffold’s preorganization of the two XB anion binding arms proximal to the hydrophobic fullerene surface and possible additional anion−π interactions are most likely responsible for the enhanced binding of **1** and a unique iodide binding selectivity of **2**.

**Table 1:** Halide Anion Association Constants (*K*_a_, M^–1^) for Receptors **1**, **2**, and **16**

anion		**1**^a^	**2**^a^	**16**^a^
Cl^–^	*K*1:1	3600 ± 300	2400 ± 100	2700 ± 100
	*K*1:2	150 ± 10	200 ± 30	140 ± 10
Br^–^	*K*1:1	5200 ± 800	2900 ± 300	4000 ± 400
	*K*1:2	190 ± 10	160 ± 10	170 ± 30
I^–^	*K*1:1	4600 ± 100	3400 ± 100	3200 ± 300
	*K*1:2	180 ± 20	140 ± 20	160 ± 10

a Solvent: 3:1 CDCl_3_:CD_3_CN at 298 K. Values reported as the mean and the standard error of the mean from independently repeated experiments.

**Figure 6:**
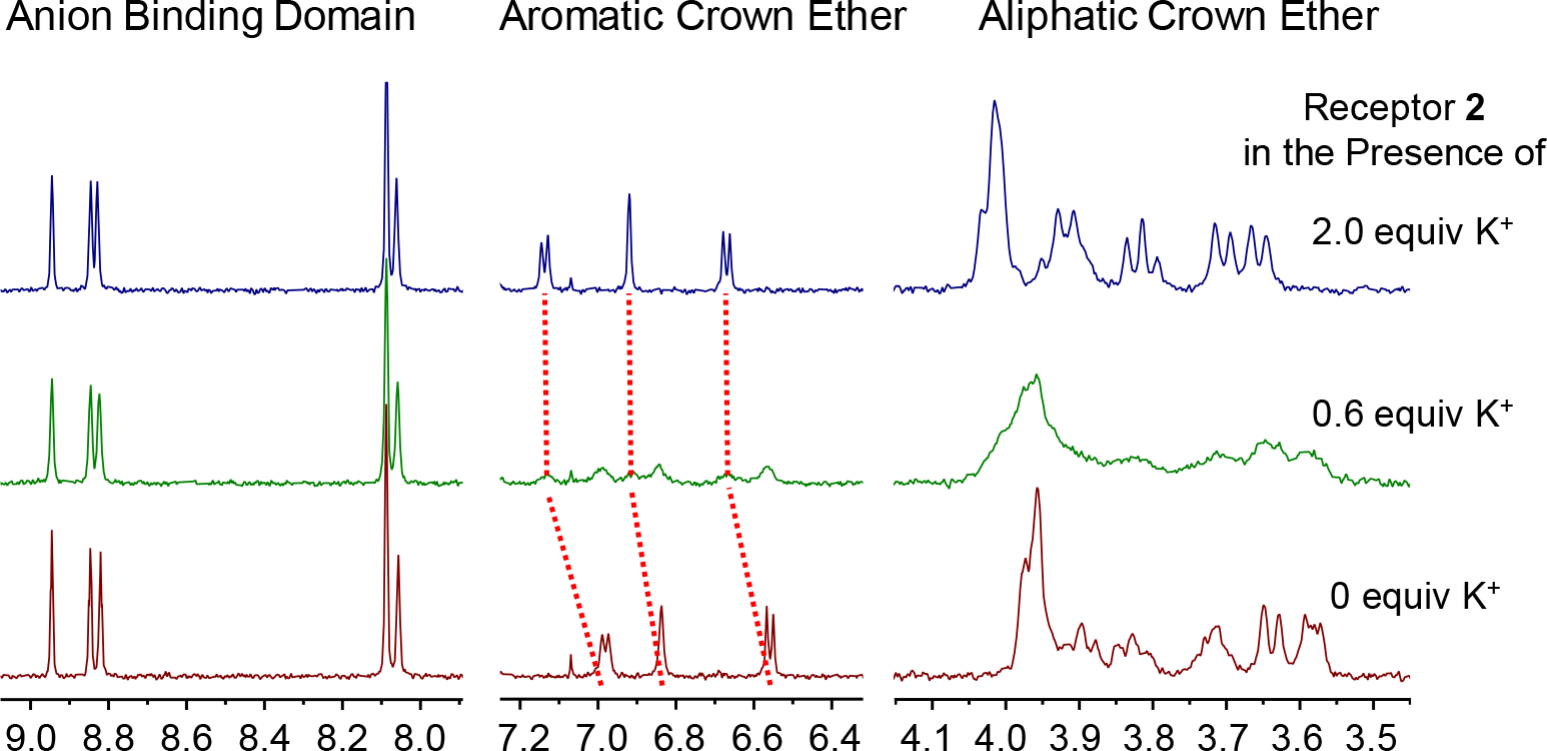
Truncated ^1^H NMR spectra of receptor **2** in 3:1 CDCl_3_:CD_3_CN with an increasing amount of KBAr_4_^F^. Broadening of the aliphatic crown ether signals hinders their assignment.

Dibenzo-18-crown-6 is known for its high affinity for potassium cations. Therefore, the K^+^ binding properties of the fullerene containing receptors **1** and **2** were also investigated by ^1^H NMR titration experiments in 3:1 CDCl_3_:CD_3_CN. Addition of KBAr_4_^F^ to solutions of free receptors **1** or **2** resulted in significant perturbations of the crown ether cation binding domain chemical shifts. With the first aliquots of KBAr_4_^F^, notable signal broadening was observed, and a new set of signals emerged due to slow exchange on the NMR time scale (Figure 6). In particular, the aromatic proton signals of the receptor’s DB18C6 motif experienced notable downfield shifts (Δδ ≈ 0.10 ppm), concomitant with −OCH_2_– crown ether perturbations, which due to significant broadening were difficult to follow. After 1.4 equiv of K^+^, however, no further changes were observed, which is indicative of strong binding in a 1:1 stoichiometric host–guest complex. Importantly, no significant changes (Δδ < 0.01 ppm) were observed in the proton signals of the XB anion binding domains of both receptors. This observation further corroborates a good separation of the anion binding domain from the cation one.

**Figure 7:**
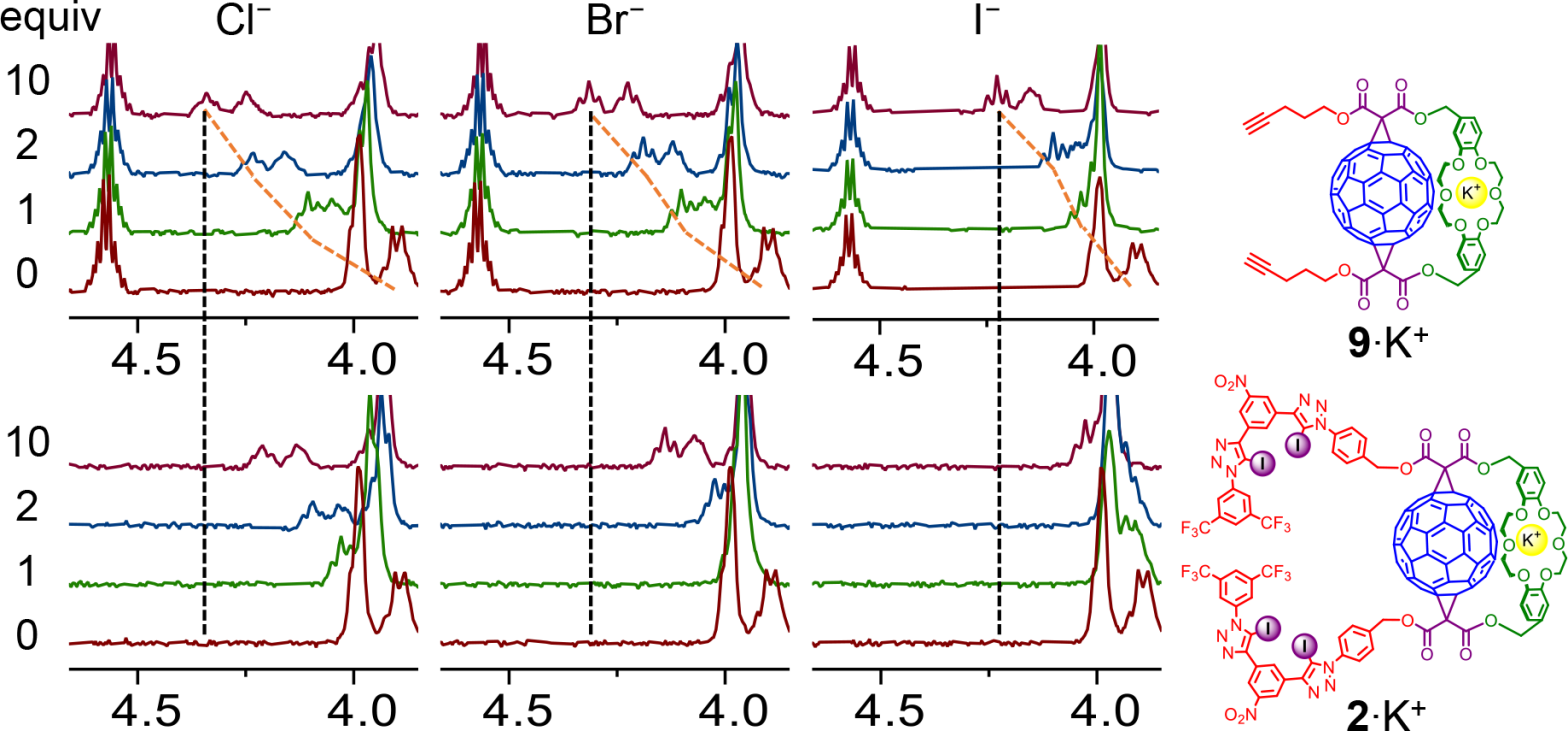
Comparison of crown ether regions during ^1^H NMR titrations of receptors **9** (top) and **2** (bottom) in the presence of 1 equiv of K^+^ with TBA salts of Cl^–^, Br^–^, and I^–^.

To investigate the ion-pair binding properties of receptors **1** and **2**, halide anion ^1^H NMR titration experiments in the presence of 1 equiv of KBAr_4_^F^ were undertaken. Addition of TBAI to a solution of K^+^ complexed **1** or **2** caused perturbations of the XB anion binding sites, confirming that XB donors are involved in anion binding. In fact, these shift patterns were qualitatively similar to those observed during titrations of the free receptors. However, small changes were also observed in the −OCH_2_– proton signals of the crown ether cation binding domain. Notably, the downfield perturbations of the −OCH_2_– crown ether signals around 3.90 ppm were larger during titrations with bromide and even greater with chloride (Figure 7). This perturbation pattern cannot be explained by simple potassium cation decomplexation of the crown ether and precipitation of the potassium halide salt (compare with the spectra shown in Figure 6).

The aforementioned observations suggest that crown ether bound K^+^ is directly involved in complexation of the anions. Interestingly, a qualitatively similar perturbation pattern of the crown ether signals was also observed during analogous titrations of K^+^ complexed C_60_-DB18C6 adduct **9**, which does not contain a XB anion binding domain (Figure 7). In this case, however, the overall signal shifts were more pronounced. In the presence of 1 equiv of KBAr_4_^F^ receptor **9** forms 1:1 stoichiometric halide complexes with the preference for hard small anions: Cl^–^ > Br^–^ > I^–^ in 3:1 CDCl_3_:CD_3_CN, as determined by the Bindfit analysis of binding isotherms (Table 2). This assembly is driven predominantly by electrostatic interactions with the crown ether complexed potassium cation resulting in the formation of a close contact ion-pair.

**Table 2:** Overall Anion Association Constants (*K*_1:1_, *K*_1:2_/M^–1^) for Receptors **1**, **2**, and **9** in the Presence of 1 equiv of K^+^

anion		**1**^a^	**2**^b^	**9**^b^
Cl^–^	*K*1:1	15100 ± 300	9300 ± 300	5000 ± 100
	*K*1:2	490 ± 30	400 ± 50	–b
Br^–^	*K*1:1	25600 ± 1500	8300 ± 300	2700 ± 100
	*K*1:2	330 ± 50	280 ± 30	–b
I^–^	*K*1:1	36000 ± 2800	19800 ± 700	1400 ± 100
	*K*1:2	600 ± 30	610 ± 60	–b

a Solvent: 3:1 CDCl_3_:CD_3_CN at 298 K. Values reported as the mean and the standard error of the mean from independently repeated experiments.

b Not formed.

**Figure 8:**
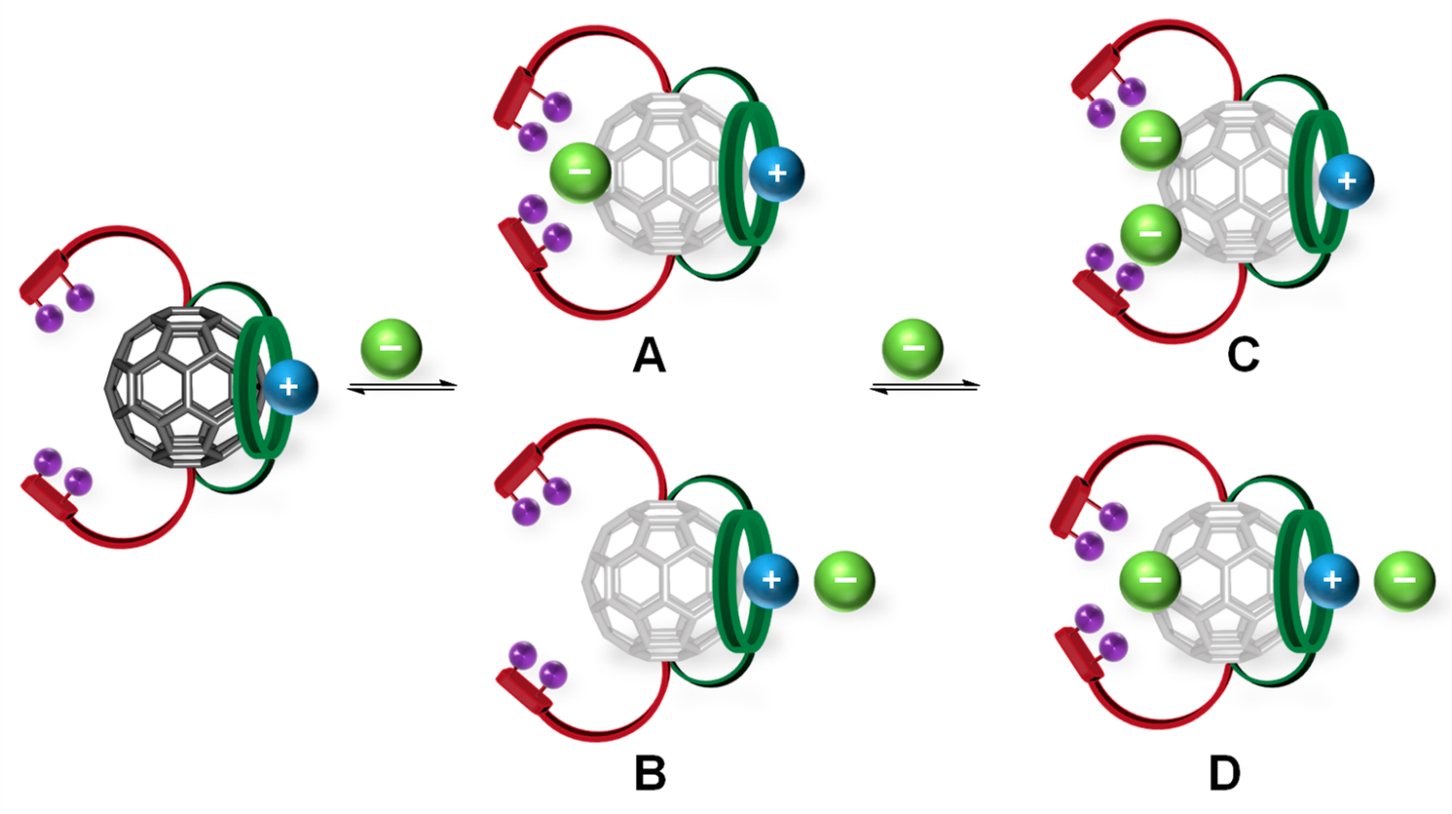
Proposed anion binding modes of fullerene-functionalized halogen-bonding heteroditopic hosts in the presence of crown ether bound potassium cation.

We suspect that such a binding mode is also present during titrations of receptors **1** and **2** with halides, particularly harder ones such as Cl^–^ and Br^–^. However, due to spatial separation of the cation and anion binding domains in receptors **1** and **2**, two distinct types of 1:1 stoichiometric complex **A** and **B** can be simultaneously formed, contributing to the experimentally determined overall 1:1 association constants (Figure 8). In binding mode **A**, the anion is exclusively bound by the XB binding site, while in mode **B** the anion associates solely with the crown ether bound potassium cation in a contact ion-pair recognition fashion.^61,62^ Similarly, upon excess addition of anion, two types of 1:2 stoichiometric complexes are possible: **C**, with two anions bound individually by the XB donor arms, and **D**, in which one anion is bound in a tetradentate XB fashion and the other anion is associating with the potassium cation (Figure 8).

This hypothesis was further corroborated by the ^1^H NMR titration of **2**·K^+^ with NO_3_^–^, which exhibits strong preference for the close contact ion-pair formation (mode **B**). During the titration with TBANO_3_ no changes were observed in the XB anion binding domain, while significant perturbation of the crown ether signals was observed until ca. 1.5 equiv of anion was added (Figure S38). Addition of up to 10 equiv of NO_3_^–^ had no further impact on the proton signals of **2**·K^+^, suggesting the oxoanion forms only the 1:1 stoichiometric anion complex of mode **B**. Interestingly, addition of 2 equiv of TBACl at the end of the titration with NO_3_^–^ (10 equiv) induced perturbations of XB binding domain signals, and no changes of crown ether signals were observed. This confirms the independence of the anion binding sites and formation of type **D** complex with chloride occupying the XB binding domain and nitrate forming the contact ion-pair with the crown ether bound K^+^.

Quantitative analysis of the halide binding isotherms in the presence of 1 equiv of KBAr_4_^F^, using a 1:2 stoichiometric host–guest model, gave overall association constant values shown in Table 2, where *K*_1:1_ represents the sum of 1:1 stoichiometric anion binding modes **A** and **B** and *K*_1:2_ the sum of 1:2 stoichiometric anion binding modes **C** and **D**. Comparing Tables 1 and 2, the presence of the complexed K^+^ in the respective C_60_-DB18C6 cation binding domain of the XB receptors **1** and **2** results in a significant enhancement of halide association constants, particularly of *K*_1:1_. Importantly, it was possible to deconvolute and estimate the individual contributions of **A** and **B** halide binding modes to the overall *K*_1:1_ association constant value through the analysis of the chemical shifts of the crown ether protons of receptors **1** and **2** and control receptor **9** during halide anion titrations in the presence of KBAr_4_^F^ (see the Supporting Information for details). In the case of **1**, the binding mode **A** accounts for approximately 63% of the overall 1:1 chloride association constant, 83% of 1:1 bromide association constant, and more than 95% of 1:1 iodide association constant, clearly showing the preference of the heavier softer halide anions toward fullerene-assisted XB binding. Further corroborating these estimates, the values of 1:1 association constants for mode **B** of receptors **1** and **2**, obtained using this method, are in good agreement with the values obtained during titrations of control receptor **9**, which is able to bind anions only via mode **B**.

Deconvolution of the **A** and **B** binding modes of 1:1 association enabled a direct comparison of K^+^ coordination effects on the fullerene-assisted halide binding in the XB domain. The *K*_1:1_ association constant values for anion binding mode **A** (*K*_1:1_^**A**^) of both heteroditopic receptors **1** and **2** are significantly increased in the presence of cobound K^+^ (Table 3). Notably, the binding enhancement for iodide is particularly strong, resulting in a remarkably increased selectivity for this anion. In the case of receptor **2**, *K*_1:1_^**A**^ association constants increased by a factor α = 2.2 (α = *K*_1:1_^**A**^(K^+^)/*K*_1:1_) and 2.1 for chloride and bromide, respectively, while for iodide α = 5.2. Even stronger I^–^ enhancement was observed for receptor **1** (α = 7.4); however, overall selectivity of **1** for I^–^ vs other halides is reduced in comparison with **2**. In the presence of cobound K^+^, control heteroditopic receptor **16**, without the fullerene scaffold, binds all the halides significantly more strongly (α = 12.7–13.5), however notably at the expense of much lower selectivity. This is most likely due to the formation of a close contact ion-pair, resulting in stronger electrostatic interactions. The remarkable properties and influence of C_60_ for ion recognition are particularly evident in comparison of iodide binding affinity exhibited by **1** and **16**. Impressively, the iodide *K*_1:1_ association constant value of receptor **1**, whose anion and cation binding domains are separated by the fullerene, almost matches the magnitude for receptor **16**, which is capable of anion binding assisted by close contact with the crown ether cobound potassium cation. Importantly, this suggests that the polarizing C_60_ surface can elicit particularly strong interactions with polarizable soft anion species such as iodide and can transfer electrostatic effects over significant distances within the fullerene heteroditopic host design.

**Table 3:** Anion Association Constants for Receptors **1** and **2** (*K*_1:1_^**A**^, M^–1^, Anion Binding Mode A) and **16** (*K*_1:1_, M^–1^) in the Presence of 1 equiv of K^+^

	**1**			**2**			**16**	
anion	*K*1:1^**A**^ a	α^b^	*A*^c^ (%)	*K*1:1^**A**^ a	α^b^	*A*^c^ (%)	*K*1:1^**A**^ a	α^b^
Cl^–^	9500 ± 200	2.6	63	5300 ± 200	2.2	57	34200 ± 600	12.7
Br^–^	21200 ± 1300	4.1	83	6100 ± 200	2.1	73	54000 ± 4600	13.5
I^–^	34200 ± 2700	7.4	>95	17800 ± 600	5.2	>90	43100 ± 3700	13.5

a Solvent: 3:1 CDCl_3_:CD_3_CN at 298 K. Values reported as the mean and the standard error of the mean from independently repeated experiments.

b Binding enhancement factors in the presence of K^+^.

c Contributions of anion binding mode **A** to overall 1:1 binding.

### Computational Analysis

Having demonstrated the unique ion-pair recognition properties of fullerene-containing heteroditopic receptors **1** and **2**, DFT calculations were undertaken to gain insight into the electronic and structural aspects of ion-pair complexation. In the computational analysis, we focused on receptor **2**, which manifested the highest selectivity, presumably due to the closer proximity of the fullerene surface. DFT calculations in the gas-phase were performed with Gaussian16,^63^ using the M06-2X functional, chosen for its ability to accurately describe halogen bonds and π–π stacking interactions.^64−66^ The Def2-SVP basis set was selected,^67^ except for the anions and iodine binding units, which were described with the Def2-TZVPD basis set,^67,68^ taken from the Basis Set Exchange website.^69−71^ This combination was employed to balance the accurate description of the noncovalent interactions and the structural features of the large receptors **1** and **2**.

**Figure 9:**
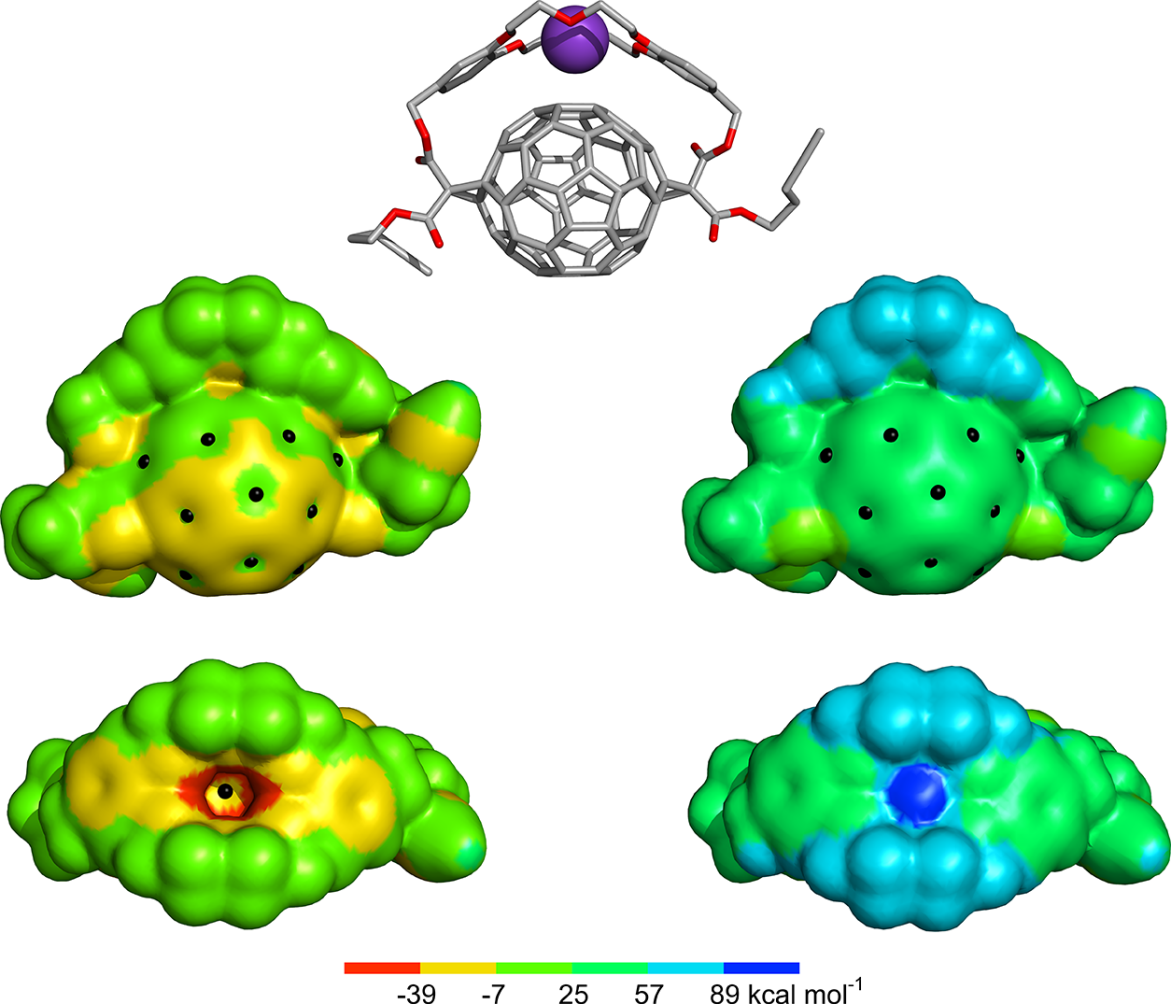
DFT-optimized structure of **9**·K^+^ (center, top), together with the MEP surfaces calculated on the adduct free of potassium (left) or on the complex (right), in lateral and top views. The MEP surfaces are rendered at the 0.001 electrons Bohr^–3^ contour and the π-holes on the surface of C_60_ are identified as black dots.

The MEP surface of C_60_ (Figure 1) exhibits distinct electrophilic regions, with π-holes situated above its 12 five-membered (C_5_) rings and 20 six-membered (C_6_) rings. These π-holes have molecular surface electrostatic potential (*V*_S_) values ranging between 7.2 and 7.9 kcal mol^–1^. For comparison, **9** and its K^+^ complex were also optimized by DFT (Figure 9). The DB18C6 motif in adduct **9** has a significant effect on the electrostatic potential map, resulting in a nearly negatively charged fullerene surface. The exposed surface of the fullerene displays several π-holes with *V*_S_ values ranging between −5.5 and −0.2 kcal mol^–1^. The lowest *V*_S_ values of **9** were found between the oxygen atoms of the crown ether cavity, varying between −58.6 and −57.5 kcal mol^–1^. The MEP surface of **9**’s C_60_ moiety has an additional negative point of *V*_S_ with −29.5 kcal mol^–1^, perpendicular to the C_6_ ring just below the crown ether. This electron-rich site is perfectly prepared for the coordination of K^+^, as evidenced by a computed K^+^···C_6_ distance of 2.93 Å in **9**·K^+^. The potassium cation binding induces a significant redistribution of the electrostatic potential in **9**, with the C_60_ π-holes’ *V*_S_ values now ranging from 30.4 to 47.2 kcal mol^–1^. For comparison, typical π-hole donors trifluoro-1,3,5-triazene or hexafluorobenzene,^72^ investigated at the M06-2X/Def2-SVP theory level, respectively display *V*_S,max_ values of 40.9 and 21.5 kcal mol^–1^. However, in complex **9**·K^+^, the *V*_S,max_ of 111.8 kcal mol^–1^ is found over the metal cation, which explains its strong tendency to form close putative ion-pair contacts with halides, as depicted in Figure S71 with the DFT optimized structures of **9**·K·X (X = Cl, Br, or I) complexes. The computed K^+^···X^–^ distances (Cl^–^: 2.85; Br^–^: 3.02; I^–^: 3.23 Å) mirror the anion’s size, with the shortest contact corresponding to the highest association constant, found for **9**·KCl (Table 2).

**Figure 10:**
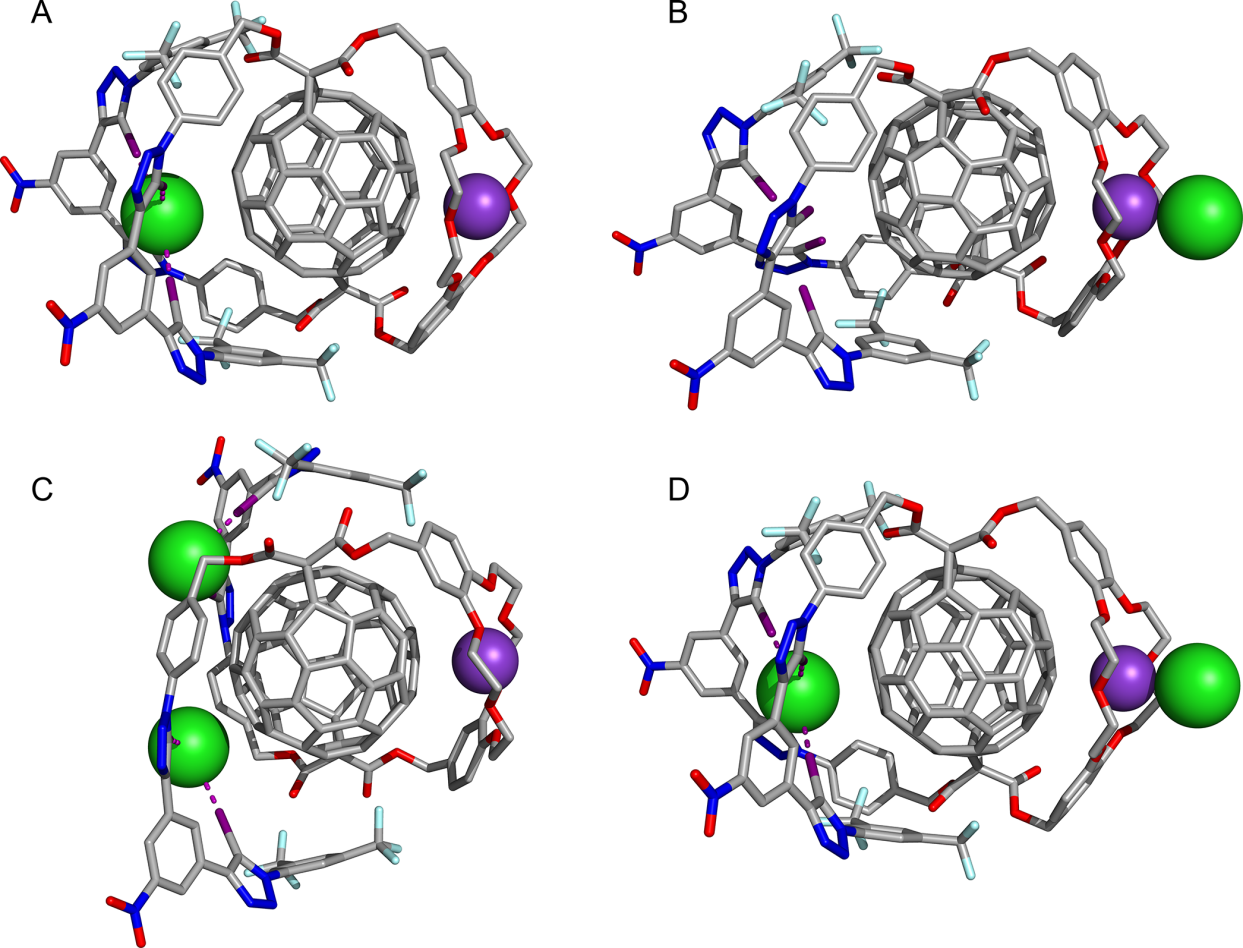
DFT-optimized anion binding modes of fullerene-functionalized halogen-bonding heteroditopic host **2** in the presence of the crown ether complexed potassium cation.

The starting geometries of **1** and **2** were generated via crude gas-phase MD simulations of KCl complexes, enforcing halogen bonds through geometric restraints, as detailed in the Supporting Information. Multiple conformations were selected and subsequently subjected to geometry optimizations using DFT. Figure 10 shows the optimized structures of chloride complexes of **2** with potassium hosted within the DB18C6 cation binding domain for the **A**–**D** anion binding scenarios, consistent with the binding modes hypothesized based on ^1^H NMR titrations, while Figures S72 and S73 show equivalent optimized binding arrangements for bromide and iodide.

Potassium binding by the DB18C6 moiety can be characterized by K^+^···C_6_ distances summarized in Table S7. In scenarios **A** and **C**, the average K^+^···C_6_ distance is ca. 3 Å, whereas in binding mode **B**, the ca. 3.5 Å average K^+^···C_6_ distance is significantly larger. Due to the close ion-pair contact in **B**, the anion pulls K^+^ from the crown ether, weakening the potential interaction with the fullerene surface.^54^ Interestingly, in scenario **D**, the K^+^···C_6_ distances have intermediate values between those computed for **B** and **A**/**C**, showing that binding in one domain can influence the behavior on the opposite side.

In binding mode **A**, the four convergent halogen bonds with chloride are not equivalent (Table S8). Two interactions have an average I···Cl^–^ distance of 3.35 Å and an average C–I···Cl^–^ angle of 163°, while the other two interactions are nearly linear with distances of 3.03 Å and angles of 173°, consistent with highly directional σ-hole XB interactions. The XB distances and angles in the bromide (3.58 Å, 162°; 3.21 Å, 174°) and iodide (3.83 Å, 162°; 3.43 Å, 176°) complexes follow a similar pattern, adjusted for the sizes of the ions (Table S8). This asymmetry is not surprising considering the differences in the iodotriazole units of the anion binding domain. One is directly connected to a strong electron-withdrawing 3,5-bis(trifluoromethyl)phenyl group, while the other is connected to an electron-rich alkyl-substituted phenyl. Importantly, the halogen-bonding anions’ recognition is assisted by anion−π interactions with short contacts between the fullerene surface and each anion, leading to the trend of C_6_···X^–^ distances Cl^–^ (3.13 Å) < Br^–^ (3.35 Å) < I^–^ (3.63 Å).

**Figure 11:**
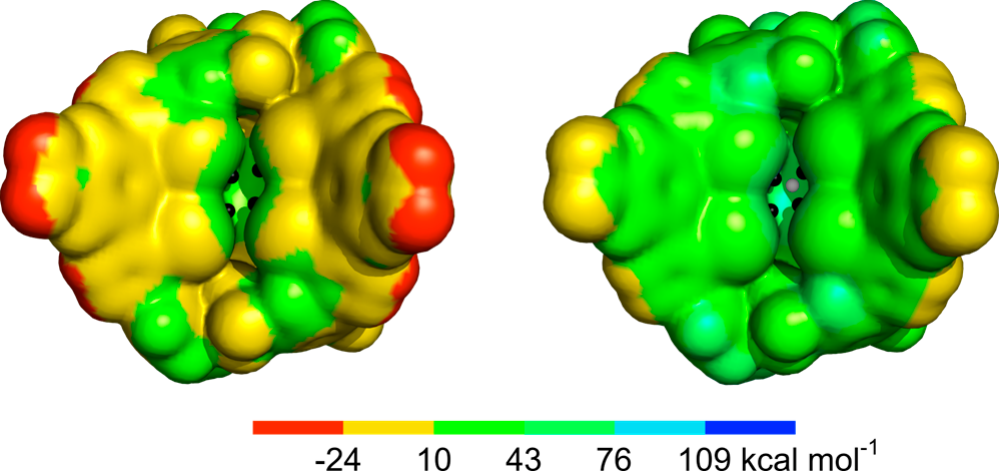
MEP surfaces calculated on the DFT-optimized geometry of **2** in binding scenario **D** (Figure 10D): free of ions (left) and in the presence of K^+^ (right). The MEP surfaces are rendered at the 0.001 electrons Bohr^–3^ contour and the σ-holes in front of the XB binding units are identified with black dots, while a neighboring π-hole on the surface of the C_60_ scaffold is identified with a white dot.

After optimization of the putative binding arrangements for the recognition of halides in binding modes **A**–**D**, the MEP maps of **2** and **2**·K^+^ were evaluated through single-point DFT calculations. To achieve this, we used the optimized structure of the Cl^–^ association in scenario **D** and removed the necessary ions (Figure 11). The most negative region of electrostatic potential on the electronic surface of **2** covers the oxygen atoms of the crown ether (including the *V*_S__,min_ of −45.8 kcal mol^–1^), while the most positive regions are found in front of the iodine binding clefts, with their σ-holes characterized by *V*_S_ values of 43.6 and 44.3 kcal mol^–1^ and of 48.2 and 48.3 kcal mol^–1^ for the iodo-triazole unit activated by neighboring −CF_3_ groups. Complexation of K^+^ in the cation binding domain of **2** leads to a significant redistribution of the MEP surface. Naturally, the *V*_S,max_ of 130.2 kcal mol^–1^ is located over the cation; however, the four XB units display augmented *V*_S_ values between 75.3 and 79.7 kcal mol^–1^. Notably, an *V*_S_ point of 61.5 kcal mol^–1^ was found positioned over the C_6_ ring in the vicinity of the preorganized XB binding units.

The strength of the XB interactions in different binding modes was further evaluated with the natural bond orbital (NBO) analysis using the second-order perturbation theory interaction energies (*E*^2^, see Table S9). The analysis of the *E*^2^ values for the interactions between the C–I antibonding orbitals of **2** and the halides’ lone pairs orbitals (n_X_ → σ*_C–I_) revealed that in binding mode **A** the total energies follow the trend Cl^–^ (37.5 kcal mol^–1^) > Br^–^ (36.3 kcal mol^–1^) > I^–^ (33.1 kcal mol^–1^). A similar analysis was also performed for the interactions between the fullerene scaffold’s π-holes and the three halides in binding modes **A** and **D**, revealing that *E*^2^ values resulting from n_X_ → σ*_C–C_ are over an order of magnitude weaker (0.5–1.3 kcal mol^–1^) than the XB interactions.

Although the computational analysis suggests that in the gas phase binding mode **B** is preferred for **2** by 3.0 (Cl^–^), 5.1 (Br^–^), and 6.2 kcal mol^–1^ (I^–^) , it is worth noting that in the case of **1**·KCl, binding mode **A** is favored by 24.9 kcal mol^–1^ relative to **B** (Figure S74).^73^ In this complex, however, the halide does not form a contact with the fullerene surface, and the receptor adopts a conformation that maximizes the strength of the halogen bonding.

Altogether, the computational analysis reveals that the fullerene platform can play a dual role in anion binding by receptors **1** and **2**. Indeed, it can be actively involved in the binding events by exploiting π-holes on its surface, but it can also serve as a bulky scaffold to preorganize the potent XB-based binding units into a tight binding cavity.

## Conclusions

For the first time, the C_60_ fullerene motif has been successfully integrated into a heteroditopic ion-pair host design. The combination of highly potent XB donors and a crown ether moiety separated by C_60_ led to the rationally designed receptors with ion-pair binding properties influenced and modulated by the fullerene motif. Receptors **1** and **2**, which differ in length of linkers separating anion binding motifs from the fullerene surface, are capable of strong and more selective binding of halide anions than their non-fullerene analogue. This is achieved by the preorganization of binding units, solvent shielding, and π-hole assistance provided by the bulky and highly polarizable C_60_ architecture. Remarkable halide anion binding enhancements can be achieved by the complexation of a potassium cation by the rigid crown ether moiety located close to the fullerene surface. Notably, potassium binding by receptors **1** and **2** results in strong augmented iodide binding selectivity, with association constants matching the value of the non-fullerene heteroditopic receptor analogue which is capable of anion binding assisted by the close contact with the crown ether cobound potassium cation.

Fullerene spatial separation of XB donors and the crown ether complexed K^+^ resulted in a rare example of an artificial receptor containing two anion binding sites with opposing preferences for hard and soft halides. Detailed analysis of ^1^H NMR titration data allowed for deconvolution of the ion-pair binding modes contributing to the overall 1:1 stoichiometric halide anion binding by **1**·K^+^ and **2**·K^+^. Soft polarizable iodide was bound almost exclusively (>90%) by the tetradentate XB binding domain in proximity to the fullerene surface. This binding mode also dominated in the case of smaller and harder chloride (ca. 60%); however, a significant portion of chloride (ca. 40%) was associated in a close contact ion-pair with the crown ether bound potassium cation without assistance of the XB binding domains.

Altogether, the presented results demonstrate the unprecedented potential of fullerene surfaces in anion recognition host–guest chemistry. Importantly, polarizing the C_60_ surface via proximal cation recognition can elicit particularly strong interactions with polarizable soft anion species such as iodide and transfer electrostatic effects over significant distances. Modulating the properties of fullerene-based compounds via reversible noncovalent charged guest recognition may find future applications in molecular sensors, solar cell devices, and photodynamic therapy.

## Supporting Information Available

Experimental procedures and methods, NMR spectra, titration data, additional data, and figures (PDF)

## References

[ref1] Supramolecular Chemistry of Fullerenes and Carbon Nanotubes; Martín N., Nierengarten J.-F., Eds.; Wiley-VCH Verlag GmbH & Co. KGaA: 2012.

[ref2] Fullerenes: Chemistry and Reactions; Hirsch A., Brettreich M., Eds.; Wiley: 2004.

[ref3] Hirao T.; Haino T. Supramolecular Ensembles Formed via Calix[5]Arene Fullerene Host Guest Interactions. Chem.—Asian J. 2022, 17, e202200344 10.1002/asia.202200344. 35647739

[ref4] Pérez E. M.; Martín N. Curves Ahead: Molecular Receptors for Fullerenes Based on Concave-Convex Complementarity. Chem. Soc. Rev. 2008, 37, 1512–1519. 10.1039/b802589b. 18648677

[ref5] Yamada M. Unraveling the Nature and Strength of Non Covalent Interactions on the Surface of Fullerenes. ChemPlusChem 2023, 88, e202300062 10.1002/cplu.202300062. 36882368

[ref6] Yamada M.; Narita H.; Maeda Y. A Fullerene Based Molecular Torsion Balance for Investigating Noncovalent Interactions at the C _60_ Surface. Angew. Chem., Int. Ed. 2020, 59, 16133–16140. 10.1002/anie.202005888. 32458522

[ref7] Lu Z.; Ronson T. K.; Heard A. W.; Feldmann S.; Vanthuyne N.; Martinez A.; Nitschke J. R. Enantioselective Fullerene Functionalization through Stereochemical Information Transfer from a Self-Assembled Cage. Nat. Chem. 2023, 15, 405–412. 10.1038/s41557-022-01103-y. 36550231

[ref8] Chen Q.; Thompson A. L.; Christensen K. E.; Horton P. N.; Coles S. J.; Anderson H. L. β,β-Directly Linked Porphyrin Rings: Synthesis, Photophysical Properties, and Fullerene Binding. J. Am. Chem. Soc. 2023, 145, 11859–11865. 10.1021/jacs.3c03549. 37201942 PMC10236496

[ref9] Moreno-Simoni M.; Torres T.; De La Torre G. Subphthalocyanine Capsules: Molecular Reactors for Photoredox Transformations of Fullerenes. Chem. Sci. 2022, 13, 9249–9255. 10.1039/D2SC01931K. 36092995 PMC9384690

[ref10] Hasegawa S.; Meichsner S. L.; Holstein J. J.; Baksi A.; Kasanmascheff M.; Clever G. H. Long-Lived C_60_ Radical Anion Stabilized Inside an Electron-Deficient Coordination Cage. J. Am. Chem. Soc. 2021, 143, 9718–9723. 10.1021/jacs.1c02860. 34156243

[ref11] Rothschild D. A.; Kopcha W. P.; Tran A.; Zhang J.; Lipke M. C. Gram-Scale Synthesis of a Covalent Nanocage That Preserves the Redox Properties of Encapsulated Fullerenes. Chem. Sci. 2022, 13, 5325–5332. 10.1039/D2SC00445C. 35655559 PMC9093146

[ref12] Barendt T. A.; Myers W. K.; Cornes S. P.; Lebedeva M. A.; Porfyrakis K.; Marques I.; Félix V.; Beer P. D. The Green Box: An Electronically Versatile Perylene Diimide Macrocyclic Host for Fullerenes. J. Am. Chem. Soc. 2020, 142, 349–364. 10.1021/jacs.9b10929. 31778308

[ref13] Fuertes-Espinosa C.; García-Simón C.; Pujals M.; Garcia-Borràs M.; Gómez L.; Parella T.; Juanhuix J.; Imaz I.; Maspoch D.; Costas M.; Ribas X. Supramolecular Fullerene Sponges as Catalytic Masks for Regioselective Functionalization of C60. Chem. 2020, 6, 169–186. 10.1016/j.chempr.2019.10.010.

[ref14] Megiatto J. D.; Guldi D. M.; Schuster D. I. Design, Synthesis and Photoinduced Processes in Molecular Interlocked Photosynthetic [60]Fullerene Systems. Chem. Soc. Rev. 2020, 49, 8–20. 10.1039/C9CS00638A. 31808480

[ref15] Pan Y.; Liu X.; Zhang W.; Liu Z.; Zeng G.; Shao B.; Liang Q.; He Q.; Yuan X.; Huang D.; Chen M. Advances in Photocatalysis Based on Fullerene C60 and Its Derivatives: Properties, Mechanism, Synthesis, and Applications. Appl. Catal., B 2020, 265, 118579 10.1016/j.apcatb.2019.118579.

[ref16] Guldi D. M.; Illescas B. M.; Atienza C. M.; Wielopolski M.; Martín N. Fullerene for Organic Electronics. Chem. Soc. Rev. 2009, 38, 1587–1597. 10.1039/b900402p. 19587954

[ref17] Lee C.; Seo Y.; Han J.; Hwang J.; Jeon I. Perspectives on Critical Properties of Fullerene Derivatives for Rechargeable Battery Applications. Carbon 2023, 210, 118041 10.1016/j.carbon.2023.118041.

[ref18] Collavini S.; Delgado J. L. Fullerenes: The Stars of Photovoltaics. Sustain. Energy Fuels 2018, 2, 2480–2493. 10.1039/C8SE00254A.

[ref19] Gatti T.; Menna E.; Meneghetti M.; Maggini M.; Petrozza A.; Lamberti F. The Renaissance of Fullerenes with Perovskite Solar Cells. Nano Energy 2017, 41, 84–100. 10.1016/j.nanoen.2017.09.016.

[ref20] Castro E.; Garcia A. H.; Zavala G.; Echegoyen L. Fullerenes in Biology and Medicine. J. Mater. Chem. B 2017, 5, 6523–6535. 10.1039/C7TB00855D. 29225883 PMC5716489

[ref21] Lebedeva M. A.; Chamberlain T. W.; Khlobystov A. N. Harnessing the Synergistic and Complementary Properties of Fullerene and Transition-Metal Compounds for Nanomaterial Applications. Chem. Rev. 2015, 115, 11301–11351. 10.1021/acs.chemrev.5b00005. 26421732

[ref22] Babu S. S.; Möhwald H.; Nakanishi T. Recent Progress in Morphology Control of Supramolecular Fullerene Assemblies and Its Applications. Chem. Soc. Rev. 2010, 39, 4021–4035. 10.1039/c000680g. 20865187

[ref23] Martín N.; Sánchez L.; Herranz M. Á.; Illescas B.; Guldi D. M. Electronic Communication in Tetrathiafulvalene (TTF)/C_60_ Systems: Toward Molecular Solar Energy Conversion Materials?. Acc. Chem. Res. 2007, 40, 1015–1024. 10.1021/ar700026t. 17602676

[ref24] Chen D.; Liu S.; Chen D.; Liu J.; Wu J.; Wang H.; Su Y.; Kwak G.; Zuo X.; Rao D.; Cui H.; Shu C.; Suk J. S. A Two Pronged Pulmonary Gene Delivery Strategy: A Surface Modified Fullerene Nanoparticle and a Hypotonic Vehicle. Angew. Chem., Int. Ed. 2021, 60, 15225–15229. 10.1002/anie.202101732. PMC8238871 33855792

[ref25] Izquierdo M.; Platzer B.; Stasyuk A. J.; Stasyuk O. A.; Voityuk A. A.; Cuesta S.; Solà M.; Guldi D. M.; Martín N. All Fullerene Electron Donor-Acceptor Conjugates. Angew. Chem., Int. Ed. 2019, 58, 6932–6937. 10.1002/anie.201901863. 30835927

[ref26] Barendt T. A.; Rašović I.; Lebedeva M. A.; Farrow G. A.; Auty A.; Chekulaev D.; Sazanovich I. V.; Weinstein J. A.; Porfyrakis K.; Beer P. D. Anion-Mediated Photophysical Behavior in a C_60_ Fullerene [3]Rotaxane Shuttle. J. Am. Chem. Soc. 2018, 140, 1924–1936. 10.1021/jacs.7b12819. 29337535

[ref27] Zhang Y.; Wang D.; Wang W. Beyond the σ-Hole and π-Hole: The Origin of the Very Large Electrophilic Regions of Fullerenes and Carbon Nanotubes. Comput. Theor. Chem. 2018, 1128, 56–59. 10.1016/j.comptc.2018.02.011.

[ref28] López-Andarias J.; Bauzá A.; Sakai N.; Frontera A.; Matile S. Remote Control of Anion-π Catalysis on Fullerene-Centered Catalytic Triads. Angew. Chem., Int. Ed. 2018, 57, 10883–10887. 10.1002/anie.201804092. PMC6120490 29806724

[ref29] Zhang X.; Liu L.; López-Andarias J.; Wang C.; Sakai N.; Matile S. Anion-π Catalysis: Focus on Nonadjacent Stereocenters. Helv. Chim. Acta 2018, 101, e1700288 10.1002/hlca.201700288.

[ref30] López-Andarias J.; Frontera A.; Matile S. Anion-π Catalysis on Fullerenes. J. Am. Chem. Soc. 2017, 139, 13296–13299. 10.1021/jacs.7b08113. 28902995

[ref31] Sun X.; Chen W.; Liang L.; Hu W.; Wang H.; Pang Z.; Ye Y.; Hu X.; Wang Q.; Kong X.; Jin Y.; Lei M. Construction of Electron Transfer Network by Self-Assembly of Self-n-Doped Fullerene Ammonium Iodide. Chem. Mater. 2016, 28, 8726–8731. 10.1021/acs.chemmater.6b04056.

[ref32] Sun S.; Liu Z.; Colombo F.; Gao R.; Yu Y.; Qiu Y.; Su J.; Gan L. Open Cage Fullerene as Molecular Container for F-, Cl-, Br- and I-. Angew. Chem., Int. Ed. 2022, 61, e202212090 10.1002/anie.202212090. 36316627

[ref33] Anstöter C. S.; Rogers J. P.; Verlet J. R. R. Spectroscopic Determination of an Anion-π Bond Strength. J. Am. Chem. Soc. 2019, 141, 6132–6135. 10.1021/jacs.9b01345. 30938520

[ref34] Kepler S.; Zeller M.; Rosokha S. V. Anion-π Complexes of Halides with P-Benzoquinones: Structures, Thermodynamics, and Criteria of Charge Transfer to Electron Transfer Transition. J. Am. Chem. Soc. 2019, 141, 9338–9348. 10.1021/jacs.9b03277. 31083908

[ref35] Molina P.; Zapata F.; Caballero A. Anion Recognition Strategies Based on Combined Noncovalent Interactions. Chem. Rev. 2017, 117, 9907–9972. 10.1021/acs.chemrev.6b00814. 28665114

[ref36] Giese M.; Albrecht M.; Rissanen K. Anion-π Interactions with Fluoroarenes. Chem. Rev. 2015, 115, 8867–8895. 10.1021/acs.chemrev.5b00156. 26278927

[ref37] Sabirov D. Sh. Polarizability as a Landmark Property for Fullerene Chemistry and Materials Science. RSC Adv. 2014, 4, 44996–45028. 10.1039/C4RA06116K.

[ref38] Bąk K. M.; Patrick S. C.; Li X.; Beer P. D.; Davis J. J. Engineered Binding Microenvironments in Halogen Bonding Polymers for Enhanced Anion Sensing. Angew. Chem., Int. Ed. 2023, 62, e202300867 10.1002/anie.202300867. PMC10946961 36749115

[ref39] Liu Y.; Parks F. C.; Sheetz E. G.; Chen C.-H.; Flood A. H. Polarity-Tolerant Chloride Binding in Foldamer Capsules by Programmed Solvent-Exclusion. J. Am. Chem. Soc. 2021, 143, 3191–3204. 10.1021/jacs.0c12562. 33596052

[ref40] Docker A.; Marques I.; Kuhn H.; Zhang Z.; Félix V.; Beer P. D. Selective Potassium Chloride Recognition, Sensing, Extraction, and Transport Using a Chalcogen-Bonding Heteroditopic Receptor. J. Am. Chem. Soc. 2022, 144, 14778–14789. 10.1021/jacs.2c05333. 35930460 PMC9394446

[ref41] Tay H. M.; Tse Y. C.; Docker A.; Gateley C.; Thompson A. L.; Kuhn H.; Zhang Z.; Beer P. D. Halogen Bonding Heteroditopic [2]Catenanes for Recognition of Alkali Metal/Halide Ion Pairs. Angew. Chem., Int. Ed. 2023, 62, e202214785 10.1002/anie.202214785. PMC10108176 36440816

[ref42] Grauwels G.; Valkenier H.; Davis A. P.; Jabin I.; Bartik K. Repositioning Chloride Transmembrane Transporters: Transport of Organic Ion Pairs. Angew. Chem., Int. Ed. 2019, 58, 6921–6925. 10.1002/anie.201900818. 30925004

[ref43] Hale U. A.; Madhavan N. Hydrophobic Cyclic Dipeptides as M^+^/Cl^–^ Carriers. Chem. Commun. 2023, 59, 7068–7071. 10.1039/D3CC02132G. 37218278

[ref44] Walczak W.; Zakrzewski M.; Cichowicz G.; Piątek P. Complexation of 5-Aminovaleric Acid Zwitterions in Aqueous/Methanol Solution by Heterotopic Tri-Cationic Receptors. Org. Biomol. Chem. 2020, 18, 694–699. 10.1039/C9OB02234A. 31904059

[ref45] Rubio O. H.; Taouil R.; Muñiz F. M.; Monleón L. M.; Simón L.; Sanz F.; Morán J. R. A Molecular Receptor Selective for Zwitterionic Alanine. Org. Biomol. Chem. 2017, 15, 477–485. 10.1039/C6OB02237E. 27929186

[ref46] McConnell A. J.; Docker A.; Beer P. D. From Heteroditopic to Multitopic Receptors for Ion Pair Recognition: Advances in Receptor Design and Applications. ChemPlusChem. 2020, 85, 1824–1841. 10.1002/cplu.202000484. 32833334

[ref47] McConnell A. J.; Beer P. D. Heteroditopic Receptors for Ion-Pair Recognition. Angew. Chem., Int. Ed. 2012, 51, 5052–5061. 10.1002/anie.201107244. 22419667

[ref48] Kim S. K.; Sessler J. L. Ion Pair Receptors. Chem. Soc. Rev. 2010, 39, 3784–3809. 10.1039/c002694h. 20737073 PMC3016456

[ref49] Pancholi J.; Beer P. D. Halogen Bonding Motifs for Anion Recognition. Coord. Chem. Rev. 2020, 416, 213281 10.1016/j.ccr.2020.213281.

[ref50] Lim J. Y. C.; Beer P. D. Sigma-Hole Interactions in Anion Recognition. Chem. 2018, 4, 731–783. 10.1016/j.chempr.2018.02.022.

[ref51] Brown A.; Beer P. D. Halogen Bonding Anion Recognition. Chem. Commun. 2016, 52, 8645–8658. 10.1039/C6CC03638D. 27273600

[ref52] Gilday L. C.; Robinson S. W.; Barendt T. A.; Langton M. J.; Mullaney B. R.; Beer P. D. Halogen Bonding in Supramolecular Chemistry. Chem. Rev. 2015, 115, 7118–7195. 10.1021/cr500674c. 26165273

[ref53] Meijer M. D.; van Klink G. P. M.; van Koten G. Metal-Chelating Capacities Attached to Fullerenes. Coord. Chem. Rev. 2002, 230, 141–163. 10.1016/S0010-8545(01)00456-8.

[ref54] Bourgeois J.-P.; Seiler P.; Fibbioli M.; Pretsch E.; Diederich F.; Echegoyen L. Cyclophane Type Fullerene dibenzo[18]Crown 6 Conjugates with Trans 1, Trans 2, and Trans 3 Addition Patterns: Regioselective Templated Synthesis, X Ray Crystal Structure, Ionophoric Properties, and Cation Complexation Dependent Redox Behavior. Helv. Chim. Acta 1999, 82, 1572–1595. 10.1002/(SICI)1522-2675(19991006)82:10<1572::AID-HLCA1572>3.0.CO;2-B.

[ref55] Bourgeois J.-P.; Echegoyen L.; Fibbioli M.; Pretsch E.; Diederich F. Regioselective Synthesis of Trans 1 Fullerene Bis Adducts Directed by a Crown Ether Tether: Alkali Metal Cation Modulated Redox Properties of Fullerene-Crown Ether Conjugates. Angew. Chem., Int. Ed. 1998, 37, 2118–2121. 10.1002/(SICI)1521-3773(19980817)37:15<2118::AID-ANIE2118>3.0.CO;2-9. 29711055

[ref56] Bickerton L. E.; Docker A.; Sterling A. J.; Kuhn H.; Duarte F.; Beer P. D.; Langton M. J. Highly Active Halogen Bonding and Chalcogen Bonding Chloride Transporters with Non Protonophoric Activity. Chem.—Eur. J. 2021, 27, 11738–11745. 10.1002/chem.202101681. 34014001 PMC8453555

[ref57] aPalatinus L.; Chapuis G. SUPERFLIP - a Computer Program for the solution of Crystal Structures by Charge Flipping in Arbitrary Dimensions. J. Appl. Crystallogr. 2007, 40, 786–790. 10.1107/S0021889807029238.

[ref58] Iehl J.; Osinska I.; Louis R.; Holler M.; Nierengarten J.-F. A Stable Fullerene-Azide Building Block for the Construction of a Fullerene-Porphyrin Conjugate. Tetrahedron Lett. 2009, 50, 2245–2248. 10.1016/j.tetlet.2009.02.185.

[ref59] Iehl J.; de Freitas R. P.; Nierengarten J.-F. Click Chemistry with Fullerene Derivatives. Tetrahedron Lett. 2008, 49, 4063–4066. 10.1016/j.tetlet.2008.04.064.

[ref60] Pereira de Freitas R.; Iehl J.; Delavaux-Nicot B.; Nierengarten J.-F. Synthesis of Fullerene Building Blocks Bearing Alkyne or Azide Groups and Their Subsequent Functionalization by the Copper Mediated Huisgen 1,3-Dipolar Cycloaddition. Tetrahedron 2008, 64, 11409–11419. 10.1016/j.tet.2008.09.047.

[ref74] http://supramolecular.org (accessed November 28, 2023).

[ref75] Brynn Hibbert D.; Thordarson P. Chem. Commun. 2016, 52, 12792–12805. 10.1039/C6CC03888C. 27779264

[ref61] Mahoney J. M.; Beatty A. M.; Smith B. D. Selective Recognition of an Alkali Halide Contact Ion-Pair. J. Am. Chem. Soc. 2001, 123, 5847–5848. 10.1021/ja0156082. 11403637

[ref62] Li D.-H.; Smith B. D. Shape-Selective Recognition of Quaternary Ammonium Chloride Ion Pairs. J. Org. Chem. 2019, 84, 2808–2816. 10.1021/acs.joc.8b03197. 30730144

[ref63] Frisch M. J.; Trucks G. W.; Schlegel H. B.; Scuseria G. E.; Robb M. A.; Cheeseman J. R.; Scalmani G.; Barone V.; Petersson G. A.; Nakatsuji H.; Li X.; Caricato M.; Marenich A. V.; Bloino J.; Janesko B. G.; Gomperts R.; Mennucci B.; Hratchian H. P.; Ortiz J. V.; Izmaylov A. F.; Sonnenberg J. L.; Williams-Young D.; Ding F.; Lipparini F.; Egidi F.; Goings J.; Peng B.; Petrone A.; Henderson T.; Ranasinghe D.; Zakrzewski V. G.; Gao J.; Rega N.; Zheng G.; Liang W.; Hada M.; Ehara M.; Toyota K.; Fukuda R.; Hasegawa J.; Ishida M.; Nakajima T.; Honda Y.; Kitao O.; Nakai H.; Vreven T.; Throssell K.; Montgomery J. A. Jr.; Peralta J. E.; Ogliaro F.; Bearpark M. J.; Heyd J. J.; Brothers E. N.; Kudin K. N.; Staroverov V. N.; Keith T. A.; Kobayashi R.; Normand J.; Raghavachari K.; Rendell A. P.; Burant J. C.; Iyengar S. S.; Tomasi J.; Cossi M.; Millam J. M.; Klene M.; Adamo C.; Cammi R.; Ochterski J. W.; Martin R. L.; Morokuma K.; Farkas Ö.; Foresman J. B.; Fox D. J. Gaussian 16, Rev. C01.

[ref64] Zhao Y.; Truhlar D. G. The M06 Suite of Density Functionals for Main Group Thermochemistry, Thermochemical Kinetics, Noncovalent Interactions, Excited States, and Transition Elements: Two New Functionals and Systematic Testing of Four M06-Class Functionals and 12 Other Functionals. Theor. Chem. Acc. 2008, 120, 215–241. 10.1007/s00214-007-0310-x.

[ref65] Zhao Y.; Truhlar D. G. A New Local Density Functional for Main-Group Thermochemistry, Transition Metal Bonding, Thermochemical Kinetics, and Noncovalent Interactions. J. Chem. Phys. 2006, 125, 194101 10.1063/1.2370993. 17129083

[ref66] Zhao Y.; Truhlar D. G. Density Functional for Spectroscopy: No Long-Range Self-Interaction Error, Good Performance for Rydberg and Charge-Transfer States, and Better Performance on Average than B3LYP for Ground States. J. Phys. Chem. A 2006, 110, 13126–13130. 10.1021/jp066479k. 17149824

[ref67] Weigend F.; Ahlrichs R. Balanced Basis Sets of Split Valence, Triple Zeta Valence and Quadruple Zeta Valence Quality for H to Rn: Design and Assessment of Accuracy. Phys. Chem. Chem. Phys. 2005, 7, 3297 10.1039/b508541a. 16240044

[ref68] Peterson K. A.; Figgen D.; Goll E.; Stoll H.; Dolg M. Systematically Convergent Basis Sets with Relativistic Pseudopotentials. II. Small-Core Pseudopotentials and Correlation Consistent Basis Sets for the Post- *d* Group 16–18 Elements. J. Chem. Phys. 2003, 119, 11113–11123. 10.1063/1.1622924.

[ref69] Feller D. The Role of Databases in Support of Computational Chemistry Calculations. J. Comput. Chem. 1996, 17, 1571–1586. 10.1002/(SICI)1096-987X(199610)17:13<1571::AID-JCC9>3.0.CO;2-P.

[ref70] Schuchardt K. L.; Didier B. T.; Elsethagen T.; Sun L.; Gurumoorthi V.; Chase J.; Li J.; Windus T. L. Basis Set Exchange: A Community Database for Computational Sciences. J. Chem. Inf. Model. 2007, 47, 1045–1052. 10.1021/ci600510j. 17428029

[ref71] Pritchard B. P.; Altarawy D.; Didier B.; Gibson T. D.; Windus T. L. New Basis Set Exchange: An Open, Up-to-Date Resource for the Molecular Sciences Community. J. Chem. Inf. Model. 2019, 59, 4814–4820. 10.1021/acs.jcim.9b00725. 31600445

[ref72] Wang H.; Wang W.; Jin W. J. σ-Hole Bond vs π-Hole Bond: A Comparison Based on Halogen Bond. Chem. Rev. 2016, 116, 5072–5104. 10.1021/acs.chemrev.5b00527. 26886515

[ref73] The energy differences (Δ*E*_298_) were calculated from the electronic energies (*ε*_0_) corrected with the Zero-Point Energies (*ε*_ZPE_) and the Thermal Energy corrections (*E*_tot_, with contributions from translation, vibrational motion, rotational motion, and electronic motion).

