# Supplementary material for: Fullerene-Functionalized Halogen-Bonding Heteroditopic Hosts for Ion-Pair Recognition
Source: J Am Chem Soc. 2023 Dec 7;145(50):27367–79. doi: 10.1021/jacs.3c07774 (PMC10739994; doi:10.1021/jacs.3c07774)
Supplement: Supplementary file 1 — ja3c07774_si_001.pdf [file ja3c07774_si_001.pdf]

Supporting Information

Fullerene functionalized halogen bonding heteroditopic hosts for ion-pair recognition

Krzysztof M. Bąk,[a,†] Igor Marques,[b] Heike Kuhn,[a] Kirsten E. Christensen,[a] Vítor Félix,[b,*] and Paul D. Beer[a,*]

[a]Chemistry Research Laboratory, Department of Chemistry, University of Oxford, Oxford OX1 3TA, U.K.
[b]CICECO - Aveiro Institute of Materials, Department of Chemistry, University of Aveiro, 810-193 Aveiro, Portugal
Present address: [†]K.M.B. EaStChem, University of Edinburgh, Joseph Black Building, David Brewster Road, Edinburgh EH9 3FJ, U.K.

*Email: vitor.felix@ua.pt
*Email: paul.beer@chem.ox.ac.uk

Table of Contents

S1 Synthesis and Characterization 2
S1.1 General Information 2
S1.2 Synthetic Procedures and Characterization 2
S2 Anion Binding Studies by ^{1}H NMR 27
S2.1 Procedure 27
S2.2 Titrations of Receptor **1** 27
S2.3 Titrations of Receptor **2** 35
S2.4 Titrations of Receptor **9** 42
S2.5 Titrations of receptor **16** 45
S3 Deconvolution of Binding Modes 51
S3.1 Two Binding Modes in 1:1 Model 51
S3.2 Two Binding Modes in 1:2 Model 53
S3.3 Analysis of Anion Binding by Receptors **1** and **2** in Presence of K^+ 54
S4 Crystal Structure Determination 55
S5 Computational Analysis 57
S5.1 Methods 57
S5.2 Additional Figures 58
S5.3 Additional Tables 60
S6 References 62

S1 Synthesis and Characterization

S1.1 General Information

All reagents were purchased from TCI, Fluorochem, or Sigma-Aldrich and used without further purification. TLC was carried out on Merck silica gel 60 F254 plates. Preparative chromatography was done using Merck Silica Gel 60 (230-400 mesh). Dry solvents were using MBraun MPSP-800 column (SPS). TBA salts were stored in a vacuum desiccator containing phosphorus pentoxide prior to use.

NMR spectra were recorded using a Bruker AVIII 400 or a Bruker AVIII 500 spectrometer with 1H NMR titrations recorded on a Bruker AVIII 500 spectrometer at ambient temperature. Chemical shifts are reported in parts per million (ppm) and coupling constants J are given in hertz (Hz). NMR data are reported as follows: chemical shift, multiplicity (s-singlet, bs – broad signal, d – doublet, t – triplet etc.), coupling constant, integration, and assignment (in some cases). The residual signal of $CDCl_3$ solvent was used as an internal reference standard (δH = 7.2600 ppm and δC = 77.16 ppm). The HR-ESI mass spectra were obtained using a Quattro TOF mass spectrometer with methanol as a spray solvent.

S1.2 Synthetic Procedures and Characterization

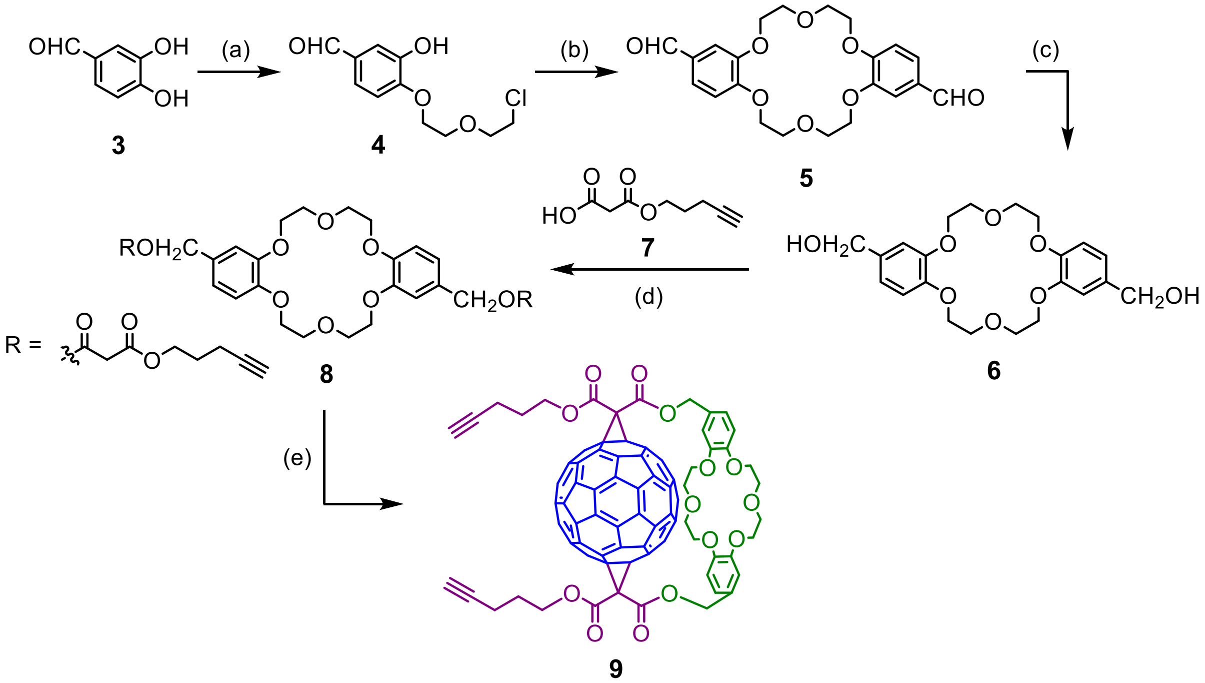

Scheme S1. Synthesis of compound **9**. (a) $(ClCH_2)_2O$, K_2CO_3, DMF, 80°C, 36h, 21%; (b) K_2CO_3, DMF, 80°C, 24h, 31%; (c) $NaBH_4$, THF/MeOH 9:1, 0°C, 2h, 62%; (d) EDC·HCl, DMAP, KPF_6, CH_2Cl_2/CH_3CN 4:1, 0°C, 48h, 76%; (e) C_{60}, I_2, DBU, KPF_6, Toluene, RT, 6h, 25%.

4-(2-(2-chloroethoxy)ethoxy)-3-hydroxybenzaldehyde **4**

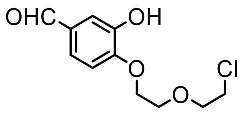

4

Based on the previously published procedure.[1] A mixture of 3,4-dihydroxybenzaldehyde **3** (20.0 g, 144.8 mmol), bis(2-chloroethyl) ether (42.4 ml, 362 mmol) and powdered K_2CO_3 in DMF (120 mL) was stirred under N_2 at 80°C for 36 h. The solid residue was filtered out and solvent was removed from

the filtrate in vacuo. 100 ml of DCM was added to the crude and the insoluble product (mixture of cis/trans isomers of crown ether **5**) was filtered out. The DCM filtrate was extracted with NaOH solution (1M, 3x50 mL). The aqueous layers were combined, acidified with HCl solution (1 M) and extracted with DCM (3x50 ml). The organic layers were combined, dried over $MgSO_4$, and solvents were removed in vacuo. Purification by flash chromatography (SiO_2; DCM/AcOEt 95:5) afforded compound **4** as a white solid (7.356 g, 21%).
^{1}H NMR (400 MHz, $CDCl_3$) δ: 9.84 (s, 1H); 7.44 (d, J = 1.9 Hz, 1H); 7.40 (dd, J = 8.2; 1.9 Hz, 1H); 7.00 (d, J = 8.2 Hz, 1H); 6.17 (bs, 1H); 4.29 (m, 2H); 3.91 (m, 2H); 3.82 (m, 2H); 3.66 (m, 2H). In accordance with previously published data.

<u>DB18C6-trans-dicarbaldehyde **5**</u>

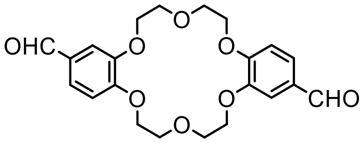

5

Based on the previously published procedure.[1] Aldehyde **4** (3.0 g, 12.3 mmol) was added to a suspension of powdered K_2CO_3 (3.40 g, 24.6 mmol) in DMF (300 mL) and stirred under N_2 at 80°C for 24 h. Next, the mixture was filtered through a short plug (SiO_2; DCM/EtOH 9:1) and solvents were removed in vacuo. Crude material was then suspended in DCM and filtered out. The collected solid was washed with water, MeOH and DCM to obtain compound **5** as a white powder (0.80 g; 31%).
^{1}H NMR (400 MHz, DMSO-d_6) δ: 9.83 (s, 2H); 7.55 (dd, J = 8.2; 2.0 Hz, 2H); 7.38 (d, J = 2 Hz, 2H); 7.17 (d, J = 8.2 Hz, 2H); 4.23-4.11 (m, 8H); 3.90-3.80 (m, 8H). In accordance with previously published data.

<u>DB18C6-trans-diol **6**</u>

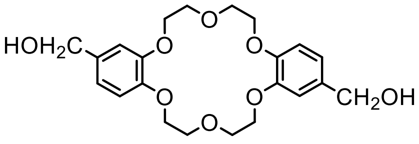

6

Based on the previously published procedure.[1] $NaBH_4$ (0.363 g, 9.61 mmol) was added to a suspension of **5** (0.500 g, 1.20 mmol) in THF/MeOH (9:1 v/v, 300 mL) at 0°C. After stirring for 2 h at room temperature, water was added and the mixture was stirred for additional 15 min. Aqueous solution was extracted with AcOEt, and then with DCM. Organic layers were combined and dried over $MgSO_4$. Solvents were removed on a rotavap to obtain compound **6** as a white solid (0.311 g, 62%).
^{1}H NMR (400 MHz, DMSO-d_6) δ: 6.95-6.75(m, 6H); 5.07 (t, J = 5.6 Hz, 2H); 4.40 (d, J = 5.6 Hz, 4H); 4.09-4.01 (m, 8H); 3.88-3.78 (m, 8H). In accordance with previously published data.

<u>Mono-4-pentynyl-malonate **7**</u>

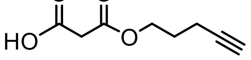

7

Based on the previously published procedure.[2] To a thoroughly dried reaction vessel, equipped with a reflux condenser, Meldrum's acid (0.856 g, 5.94 mmol) and 4-pentyl-1-ol (0.500 g, 5.94 mmol) were added followed by dry toluene (25 mL). The solution was refluxed for 2 h. Then, it was left to cool down and washed with saturated $NaHCO_3$ solution (3x30 mL). Aqueous layer was then acidified using

HCl (1 M) to a pH < 3 (release of CO_2) and extracted with DCM (3x30 mL). Organic layers were combined and solvent was removed in vacuo to obtain **7** as a colourless liquid which solidified upon standing (0.683 g, 68%).

The alternative approach using di(pent-4-yn-1-yl) malonate

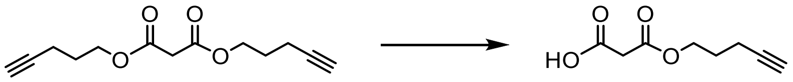

Di(pent-4-yn-1-yl) malonate was prepared according to the previously published procedure.[3] Di(pent-4-yn-1-yl) malonate (0.250 g, 1.06 mmol) was added to a reaction vessel followed by THF (10 ml) and H_2O (10 ml). The mixture was cooled down to 0°C. A solution of NaOH (0.25 M, 4.2 ml) was added dropwise, and the mixture was stirred for 1 h. Next, it was washed with EtOAc (3x20 mL). Aqueous solution was acidified using HCl (1 M) to a pH < 3 and extracted with AcOEt (3x20 mL). Organic layers were combined and dried over $MgSO_4$. Solvents were removed on a rotavap to obtain **7** as a colourless liquid which solidified upon standing (0.154 g, 86%).

^{1}H NMR (500 MHz, $CDCl_3$) δ: 9.51 (bs, 1H), 4.29 (t, J = 6.4 Hz, 2H), 3.44 (s, 2H), 2.29 (td, J = 6.4 Hz, J = 2.8 Hz, 2H), 1.97 (t, J = 2.8 Hz, 2H), 1.89 (p, J = 6.4 Hz, 2H).

^{13}C NMR (126 MHz, $CDCl_3$) δ 171.58, 166.85, 82.81, 69.38, 64.54, 40.88, 27.36, 15.18.

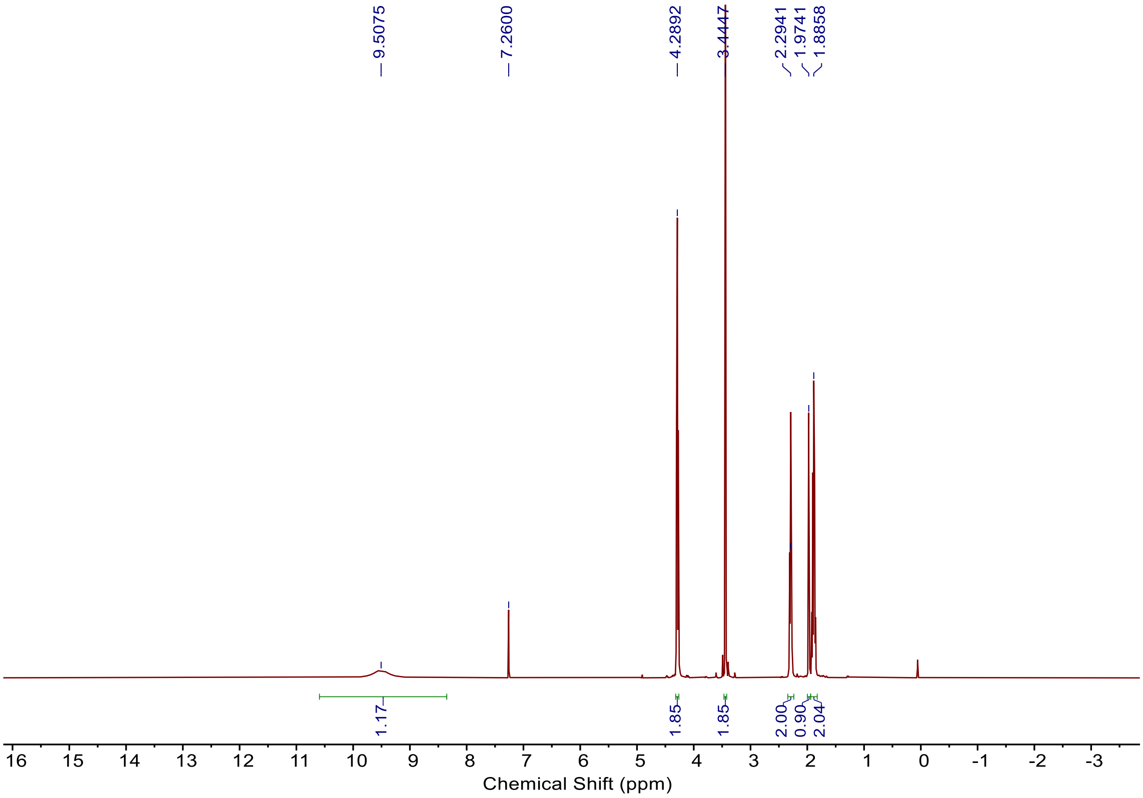

Figure S1. ^{1}H NMR spectrum of **1** in $CDCl_3$, 500 MHz, 298K.

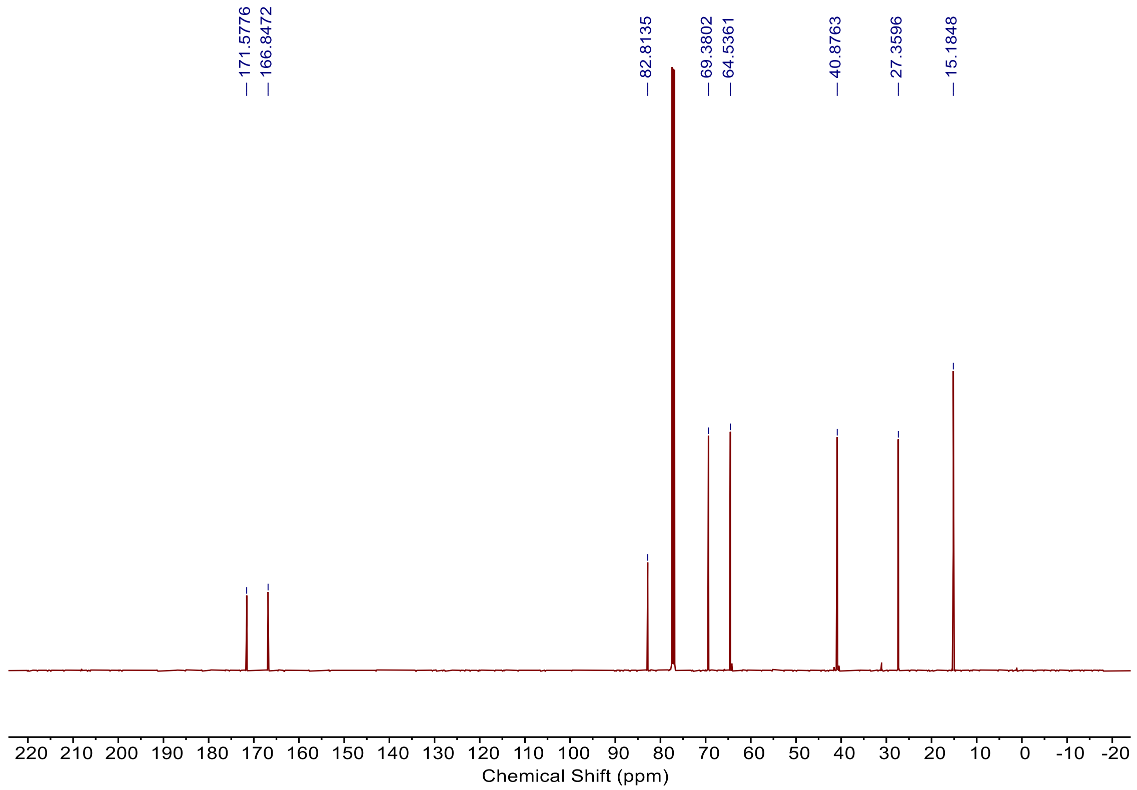

Figure S2. ^{13}C NMR spectrum of **1** in $CDCl_3$, 126 MHz, 298K.

<u>DB18C6-trans-bis(malonate)</u> **8**

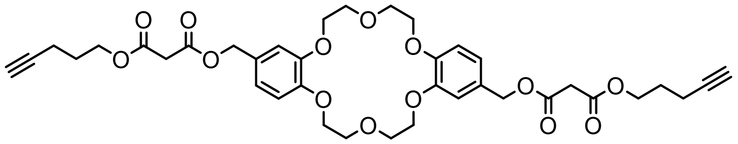

8

To a thoroughly dried reaction vessel diol **6** (200 mg, 0.476 mmol) and KPF_6 (88 mg, 0.476 mmol) were added followed by CH_3CN (4 ml). The mixture was sonicated for 5 min to solubilize substrates. Next, DCM (16 ml) was added followed by mono-malonate **7** (324 mg, 1.903 mmol) and DMAP (256 mg, 2.093 mmol). The reaction vessel was cooled down to 0°C and EDC·HCl (401 mg, 2.093 mmol) was added. The mixture was stirred in room temperature for 2 days under N_2. Then it was washed with a solution of citric acid (10%), solution of $NaHCO_3$ (saturated), H_2O, and brine. The organic layer was dried over $MgSO_4$ and solvents were removed in vacuo. The crude was purified by flash chromatography (SiO_2; DCM/MeOH 1%-3%). Final product was then dissolved in DCM and washed with water to remove K^+ salts. Solvents removal in vacuo afforded compound **8** as a white solid (262 mg; 76%).

^{1}H NMR (500 MHz, $CDCl_3$) δ: 6.92-6.85 (m, 4H), 6.82 (d, J = 8.2 Hz, 2H), 5.08 (s, 4H), 4.23 (t, J = 6.3 Hz, 4H), 4.18-4.14 (m, 8H), 4.04-4.00 (m, 8H), 3.39 (s, 4H), 2.23 (td, J = 6.9; 2.6 Hz, 4H) 1.96 (t, J = 2.6 Hz, 4H), 1.90 (p, J = 6.6 Hz; 4H).

^{13}C NMR (126 MHz, $CDCl_3$) δ: 166.51, 148.99, 148.69, 128.25, 121.98, 113.94, 113.07, 82.93, 69.85, 69.32, 68.83, 68.73, 67.42, 64.12, 41.69, 27.45, 15.17.

HR MS (ESI+ve) m/z: 763.2352 found, ($[M+K]^+$, $C_{38}H_{44}O_{14}K$ calculated 763.2363)

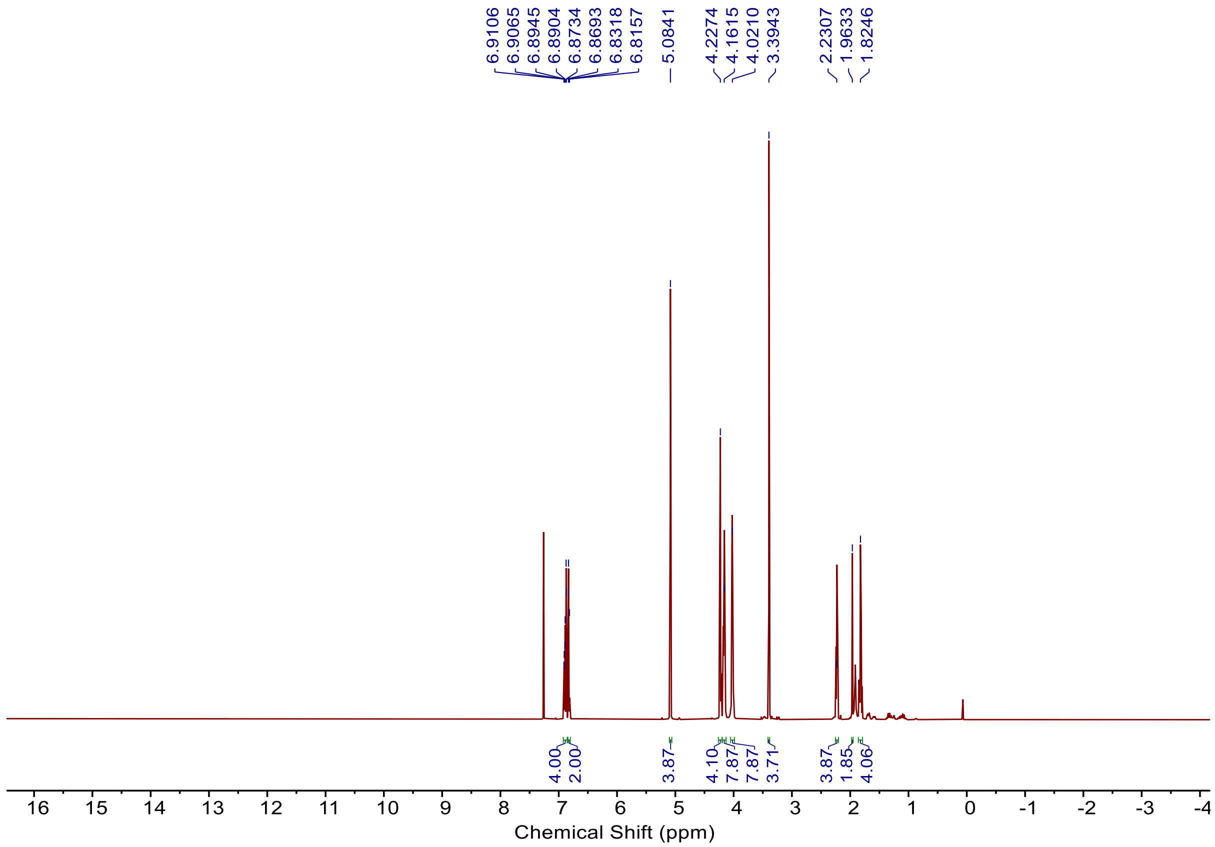

Figure S3. ^{1}H NMR spectrum of **8** in $CDCl_3$, 500 MHz, 298K.

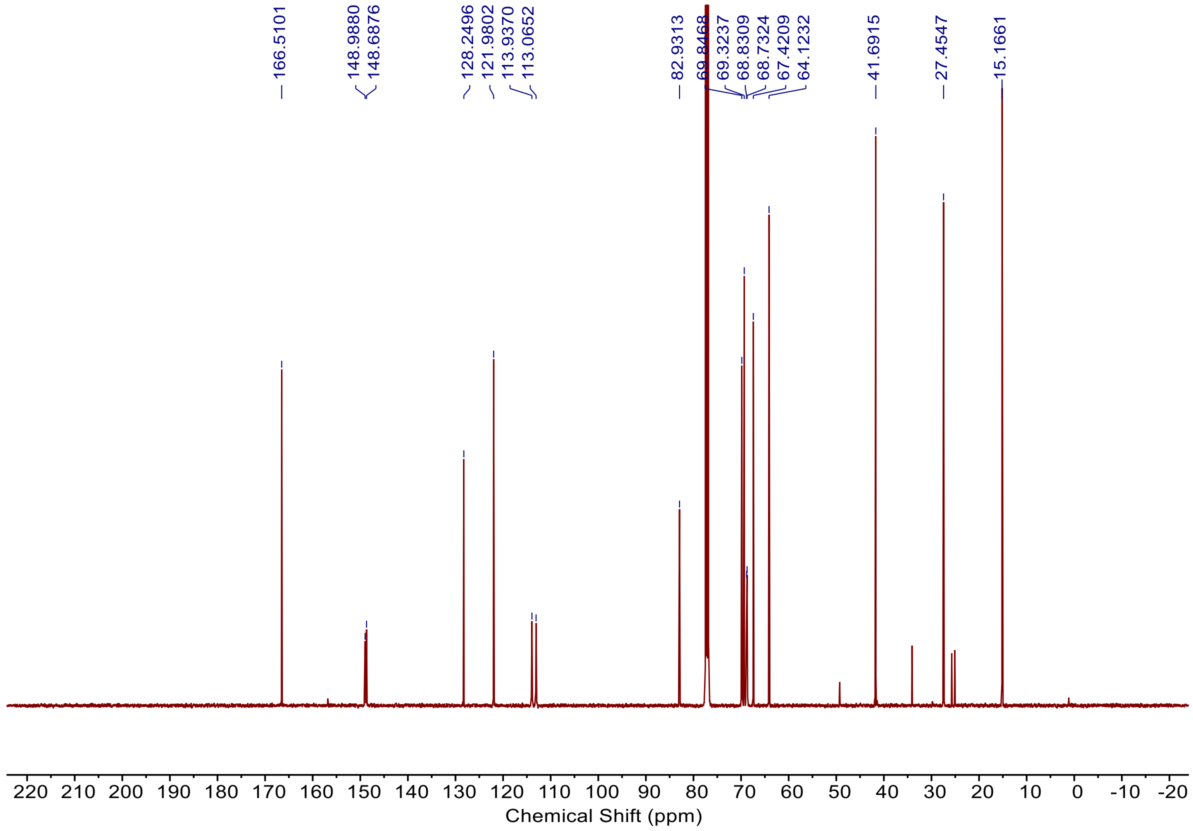

Figure S4. ^{13}C NMR spectrum of **8** in $CDCl_3$, 126 MHz, 298K.

C$_{60}$-DB18C6-bis-alkyne adduct **9**

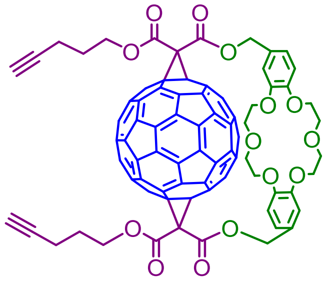

9

To a thoroughly dried reaction vessel C$_{60}$ (56 mg, 0.077 mmol) and toluene (110 mL) were added. The solution was sonicated for 10 min and stirred for at least 30 min to solubilise fullerene. In a separate dried vessel DB18C6-bis(malonate) **8** (50 mg, 0.077 mmol) and KPF_6 (142 mg, 0.77 mmol) were dissolved in acetonitrile (5 ml). To a solution of C$_{60}$, solid I_2 (39 mg, 0.154 mmol) was added followed by the solution of **8** and KPF_6 in acetonitrile. The mixture was stirred for 15 minutes and DBU (69 µL, 0.462) in DCM (1 mL) was added dropwise. After stirring for 6 h under N_2, the solution was washed with water, brine, and dried over $MgSO_4$. The crude was purified by flash chromatography (SiO_2; toluene to recover unreacted C$_{60}$, then toluene/AcOEt 1:1 to obtain the product). The final product was dissolved in DCM and washed with water to remove K^+ salts. Solvents removal in vacuo afforded compound **9** as a dark brown solid (28 mg, 25%).

^{1}H NMR (600 MHz, $CDCl_3$) δ 7.09 (dd, J = 8.2; 2.0 Hz, 2H), 6.93 (d, J = 2.0 Hz, 2H), 6.65 (d, J = 8.2 Hz, 2H), 5.78 (d, J = 10.7 Hz, 2H), 5.42 (d, J = 10.8 Hz, 2H), 4.72 (m, 4H), 4.19 (m, 2H), 4.10-3.96 (m, 8H), 3.90-3.85 (m, 2H), 3.83-3.78 (m, 2H), 3.78-3.73 (m. 2H), 2.52 (td, J = 7.0, 2.7 Hz, 4H), 2.22 – 2.14 (m, 4H), 2.09 (t, J = 2.7 Hz, 2H).

^{13}C NMR (151 MHz, $CDCl_3$) δ: 164.58, 164.43, 149.49, 148.25, 145.43, 145.38, 145.15, 144.92, 144.80, 144.58, 144.54, 144.46, 143.85, 143.81, 143.79, 143.49, 143.40, 143.29, 142.68, 142.42, 142.17, 141.27, 141.25, 141.10, 140.74, 140.65, 140.59, 137.96, 137.18, 127.95, 124.53, 114.31, 112.26, 82.83, 70.22, 70.02, 69.95, 69.78, 69.77, 69.61, 68.86, 68.07, 66.01, 45.28, 27.69, 15.49.

HR MS (ESI+ve) m/z: 1463.2302 found, ([M+Na]$^+$, $C_{98}H_{40}O_{14}Na$ calculated 1463.2310)

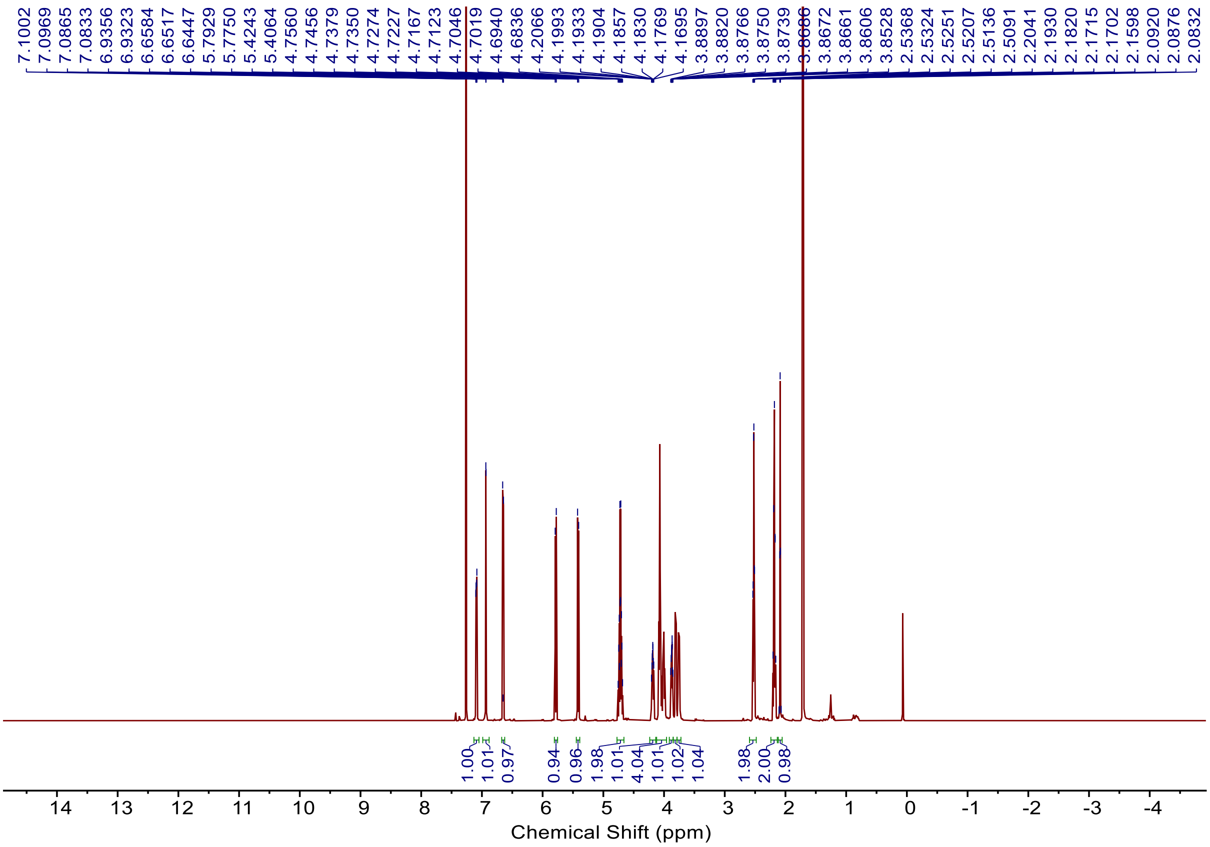

Figure S5. ^{1}H NMR spectrum of **9** in $CDCl_3$, 500 MHz, 298K.

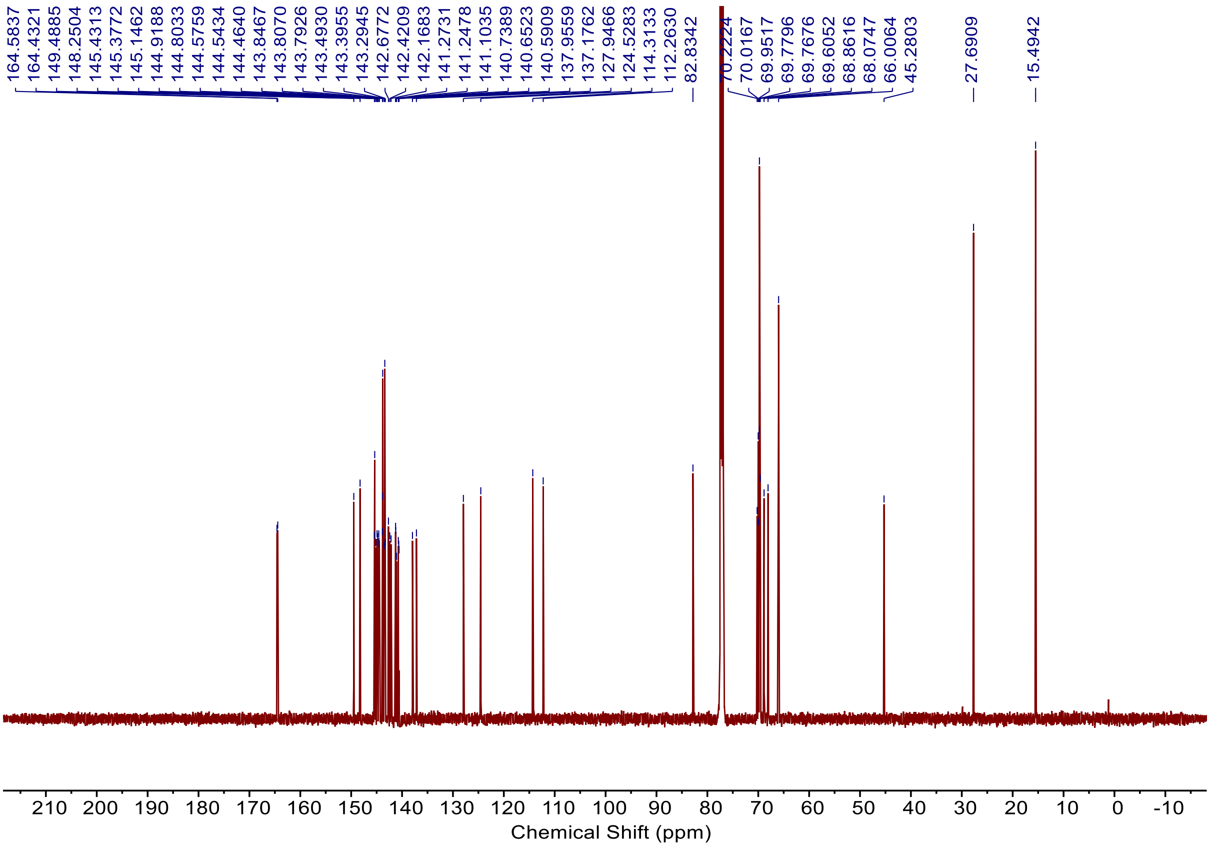

Figure S6. ^{13}C NMR spectrum of **9** in $CDCl_3$, 126 MHz, 298K.

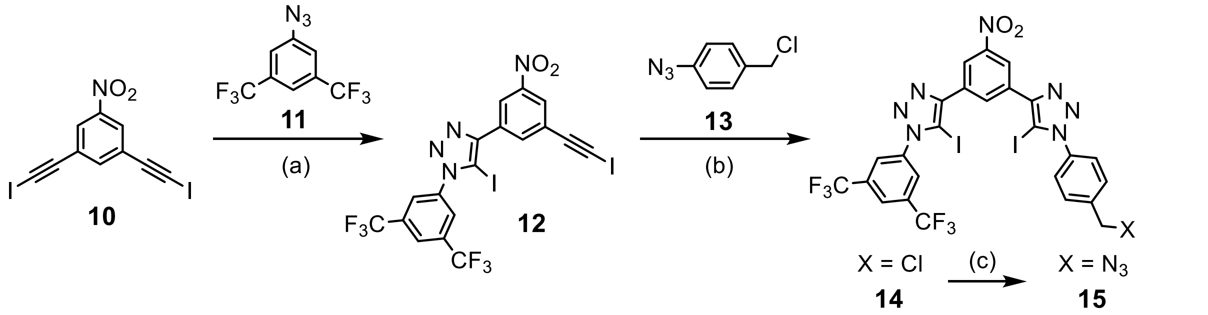

Scheme S2. Synthesis of compound **15**. (a) $[Cu(CH_3CN)_4]PF_6$, TBTA, CH_2Cl_2, RT, 16h, 65%; (b) $[Cu(CH_3CN)_4]PF_6$, TBTA, CH_2Cl_2, RT, 16h 72%; (c) NaN_3, DMF, RT, 24h, 60%; (d) $[Cu(CH_3CN)_4]PF_6$, TBTA, CH_2Cl_2, RT, 48h, 36%.

1,3-bis(iodoethynyl)-5-nitrobenzene **10**

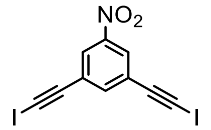

10

1,3-Bis(iodoethynyl)-5-nitrobenzene **10** was prepared according to the previously published procedure.[4] 1,3-Diethynyl-5-nitrobenzene (846 mg, 4.94 mmol) was dissolved in THF (anhydrous, 50 mL) to which CuI (94 mg, 0.49 mmol) and N-iodomorpholine hydroiodide (5.56 g, 16.3 mmol) were added. Mixture was stirred for 4 hours in the dark. Next, the reaction mixture was diluted with DCM and filtered through a pad of SiO_2. The filtrate was concentrated on a rotavap, washed with a solution of $Na_2S_2O_3$ (5%) and dried over $MgSO_4$. Solvents were removed in vacuo and crude was adsorbed onto silica. The dry loaded compound was eluted with 10% EtOAC/hexane. Solvents were removed in vacuo. Further purification by flash chromatography (SiO_2, 20% DCM/Hexane) afforded compound **10** as a pale-yellow solid (1.87 g, 90%). The compound was stored in a freezer with a minor sign of decomposition.
^{1}H NMR (400 MHz, $CDCl_3$) δ 8.19 (d, J = 1.5 Hz, 2H), 7.75 (t, J = 1.5 Hz, 1H). In accordance with previously published data.

1-azido-3,5-bis(trifluoromethyl)benzene **11**

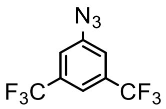

11

1-Azido-3,5-bis(trifluoromethyl)benzene **11** was prepared according to the previously published procedure.[5]

1-(3,5-bis(trifluoromethyl)phenyl)-5-iodo-4-(3-(iodoethynyl)-5-nitrophenyl)-1H-1,2,3-triazole **12**

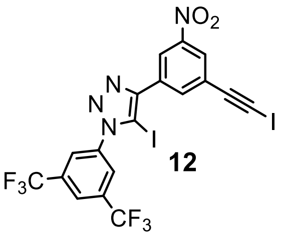

12

$[Cu(CH_3CN)_4]PF_6$ (149 mg, 0.39 mmol) and TBTA (209 mg, 0.39 mmol) were dissolved in DCM (anhydrous, 80 mL). The mixture was stirred for 30 min under N_2. Then bis-iodoalkyne **10** (1.00 g, 2.36 mmol) and azide **11** (201 mg, 0.79 mmol) were added. The reaction mixture was protected from the light and stirred overnight under N_2. Next, it was washed with aqueous EDTA (10%)/NH_3 (2%) solution (3x20 mL), followed by brine (20 mL). Organic layers were combined and dried over $MgSO_4$. The solvent was removed in vacuo to give the crude product. Purification by flash chromatography (SiO2, DCM/Hexane 2:8 to recover unreacted substrate, then DCM/Hexane 8:2) afforded product **12** as a white solid (348 mg, 65%). Used immediately in the next step.
^{1}H NMR (500 MHz, $CDCl_3$) δ 8.88 (t, J = 1.6 Hz, 1H), 8.42 (t, J = 1.6 Hz, 1H), 8.35 (t, J = 1.6 Hz, 1H), 8.14 (bs, 3H).

1-azido-4-(chloromethyl)benzene **13**

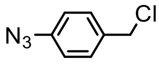

13

1-Azido-4-(chloromethyl)benzene **13** was prepared according to the previously published procedure starting from 4-aminobenzylalcohol.[6]

Bis-iodotriazole chloride **14**

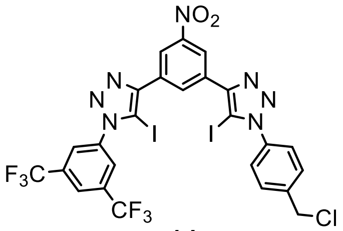

14

$[Cu(CH_3CN)_4]PF_6$ (57 mg, 0.15 mmol) and TBTA (79 mg, 0.15 mmol) were dissolved in DCM (anhydrous, 3 mL). The mixture was stirred for 30 min under N_2. Then iodoalkyne **12** (203 mg, 0.30 mmol) and azide **13** (60 mg, 0.36 mmol) were added. The reaction mixture was protected from the light and stirred overnight under N_2. Then, it was diluted with DCM (15 mL) and washed with aqueous EDTA (10%)/NH_3 (2%) solution (3x10 mL), followed by brine (10 mL). Organic layers were combined and dried over $MgSO_4$. The solvent was removed in vacuo to give the crude product. Purification by flash chromatography (SiO_2, DCM) afforded product **14** as a white solid (182 mg, 72%).
^{1}H NMR (600 MHz, $CDCl_3$) δ 9.13 (t, J = 1.6 Hz, 1H), 9.06 (t, J = 1.6 Hz, 1H), 8.99 (t, J = 1.6 Hz, 1H), 8.17 (bs, 2H), 8.14 (bs, 1H), 7.67-7.63 (m, 2H), 7.62-7.58 (m, 2H), 4.71 (s, 2H).
^{13}C NMR (151 MHz, $CDCl_3$) δ: 149.10, 148.95, 147.98, 140.27, 137.85, 136.56, 133.58 (q), 132.68, 131.94, 131.83, 129.80, 126.92, 124.39, 122.57, 122.50 (q), 122.34, 78.89, 78.36, 45.16.
^{19}F NMR (565 MHz, $CDCl_3$) δ -62.92.
HR MS (ESI+ve) m/z: 867.8645 found, ($[M+Na]^+$, $C_{25}H_{12}ClF_6I_2N_7O_2Na$ calculated 867.8627)

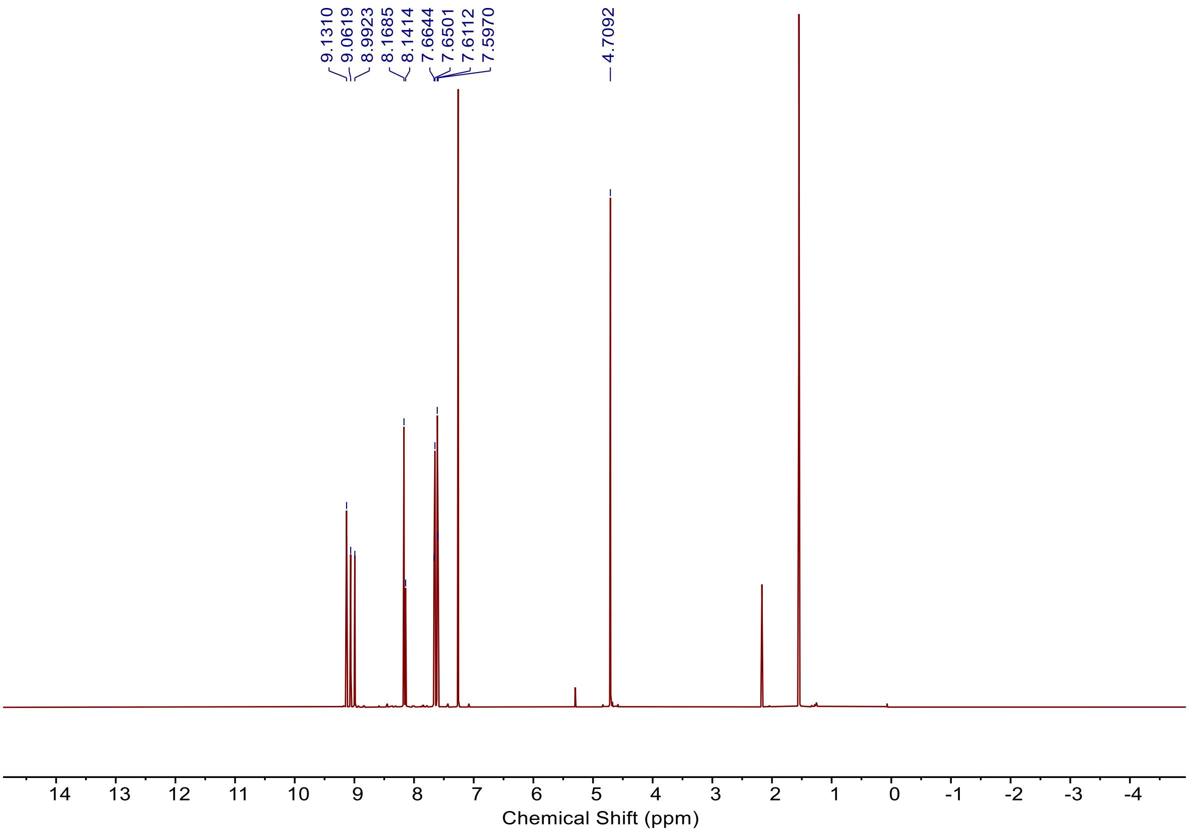

Figure S7. ^{1}H NMR spectrum of **14** in $CDCl_3$, 500 MHz, 298K.

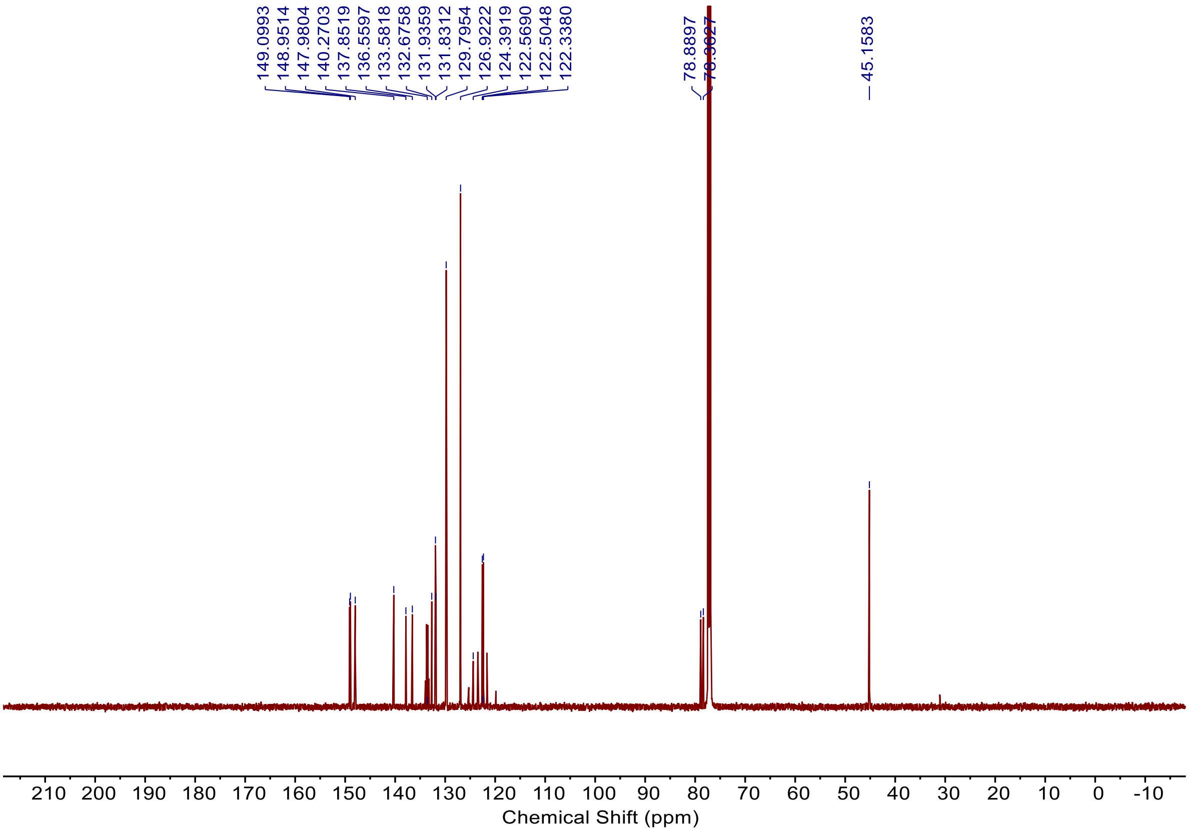

Figure S8. ^{13}C NMR spectrum of **14** in $CDCl_3$, 126 MHz, 298K.

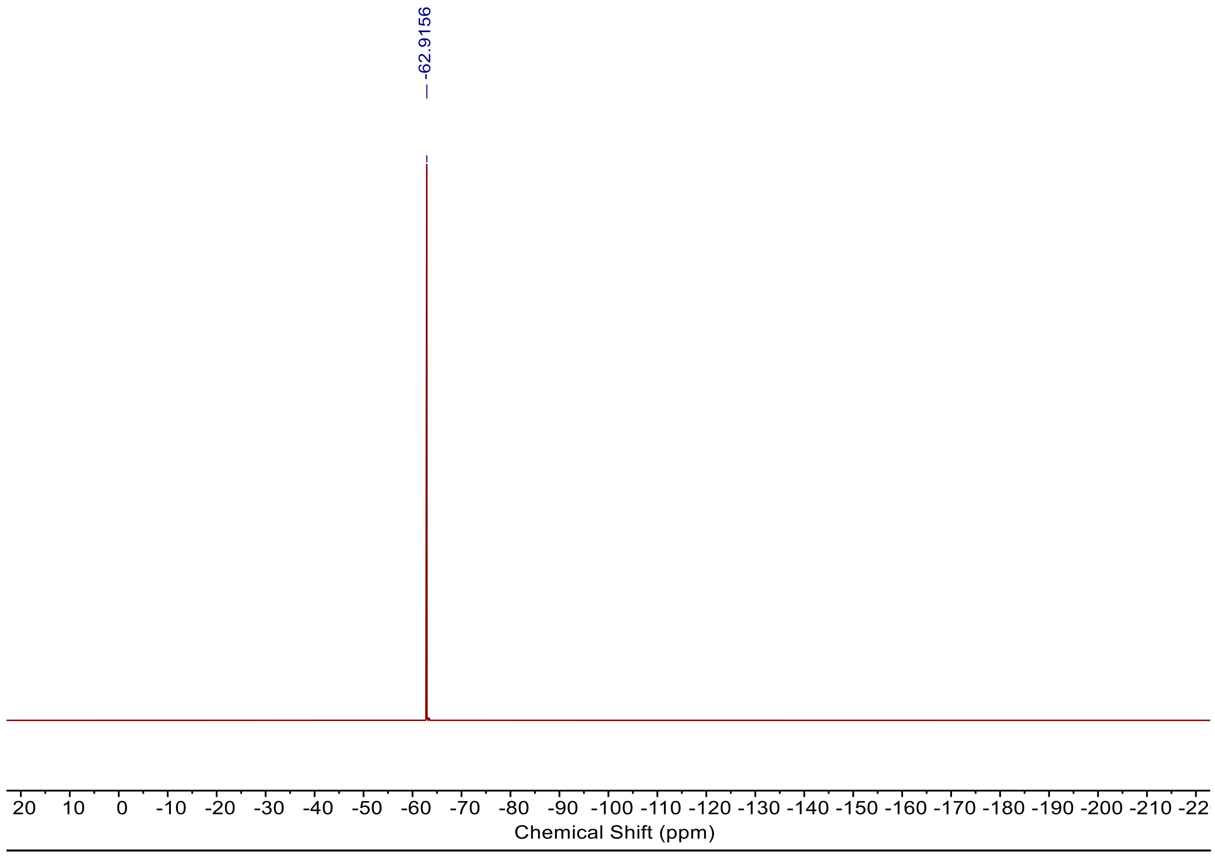

Figure S9. ^{13}F NMR spectrum of **14** in $CDCl_3$, 565 MHz, 298K.

<u>Bis-iodotriazole azide **15**</u>

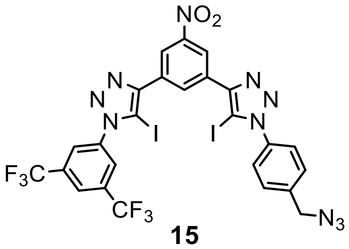

15

Chloride **14** (150 mg, 0.18 mmol) was dissolved in DMF (1 ml). Sodium azide (17 mg, 0.27 mmol) was added to the mixture, which was then left stirring for 24h in room temperature. Next, it was diluted with AcOEt (10 mL) and washed with water. The organic layer was washed with brine and dried over $MgSO_4$. Solvent was removed in vacuo to obtain the crude product. Purification by flash chromatography (SiO_2, DCM) afforded azide **15** as a solid (91 mg, 60%).

^{1}H NMR (600 MHz, $CDCl_3$) δ 9.13 (t, J = 1.6 Hz,1H), 9.06 (t, J = 1.6 Hz, 1H), 8.99 (t, J = 1.6 Hz, 1H), 8.17 (bs, 2H), 8.14 (bs, 1H), 7.65-7.61 (m, 2H), 7.61-7.57 (m, 2H), 4.54 (s, 2H).

^{13}C NMR (151 MHz, $CDCl_3$) δ: 149.09, 148.95, 147.96, 138.53, 137.85, 136.52, 133.59 (q), 132.68, 131.92, 131.83, 129.17, 127.05, 126.93, 124.39, 122.56, 122.49 (q), 122.33, 78.97, 78.38, 54.20.

^{19}F NMR (565 MHz, $CDCl_3$) δ -62.92.

HR MS (ESI+ve) m/z: 852.9213 found, ($[M+H]^+$, $C_{25}H_{13}F_6I_2N_{10}O_2$ calculated 852.9211)

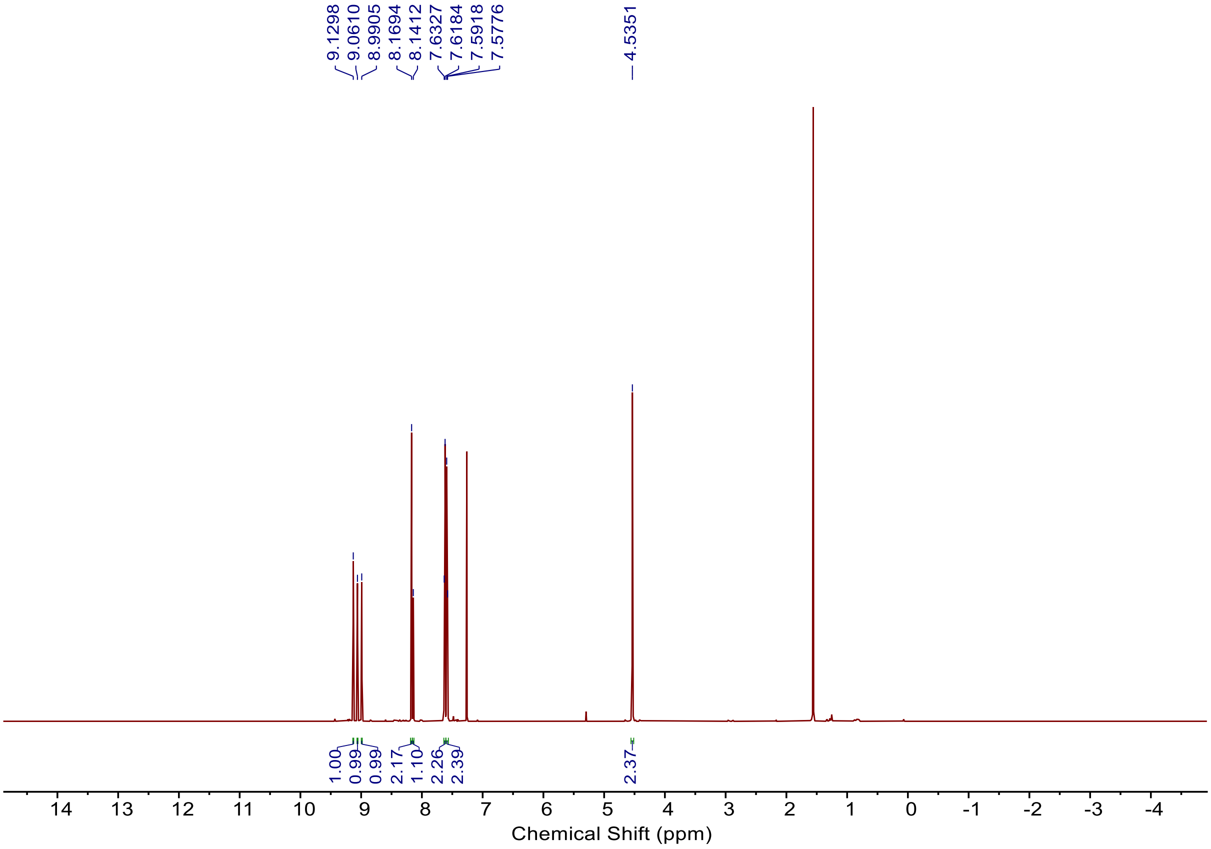

Figure S10. ^{1}H NMR spectrum of **15** in $CDCl_3$, 500 MHz, 298K.

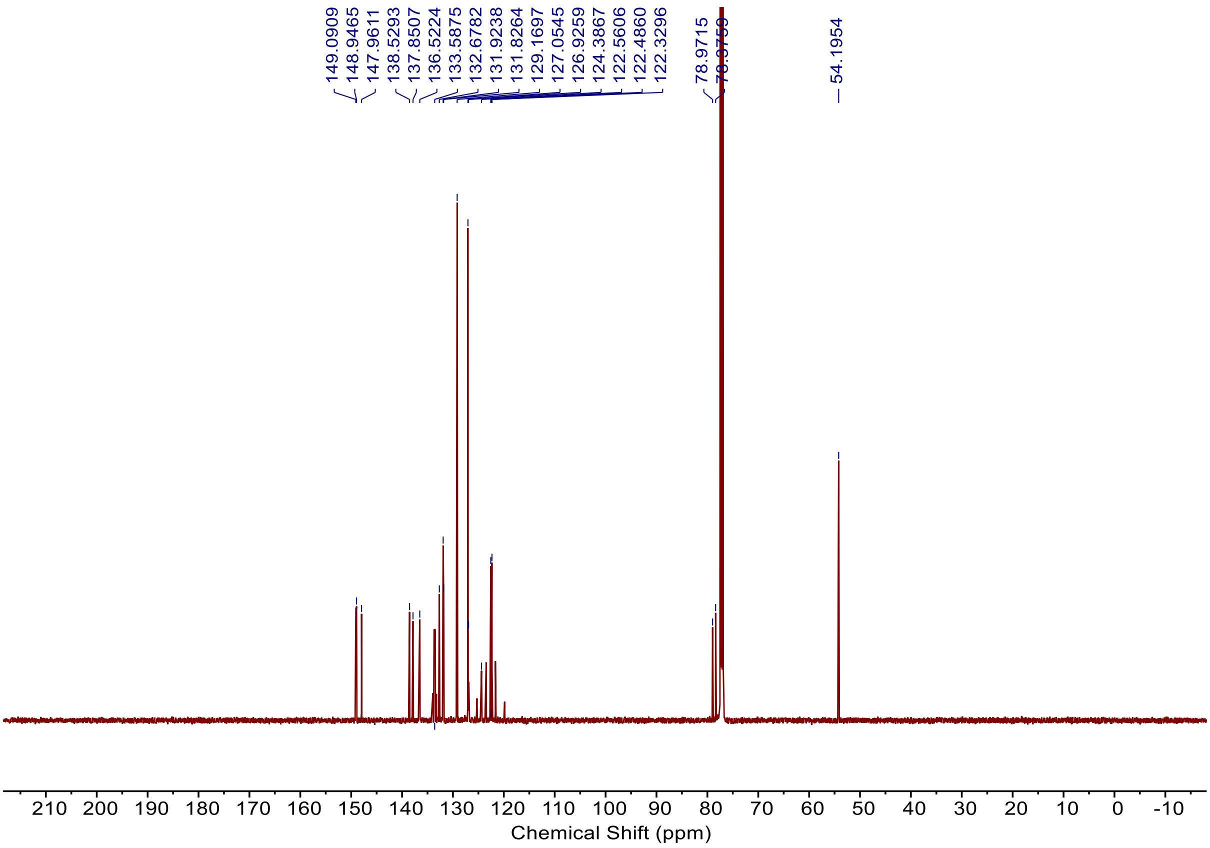

Figure S1. ^{13}C NMR spectrum of **15** in $CDCl_3$, 126 MHz, 298K.

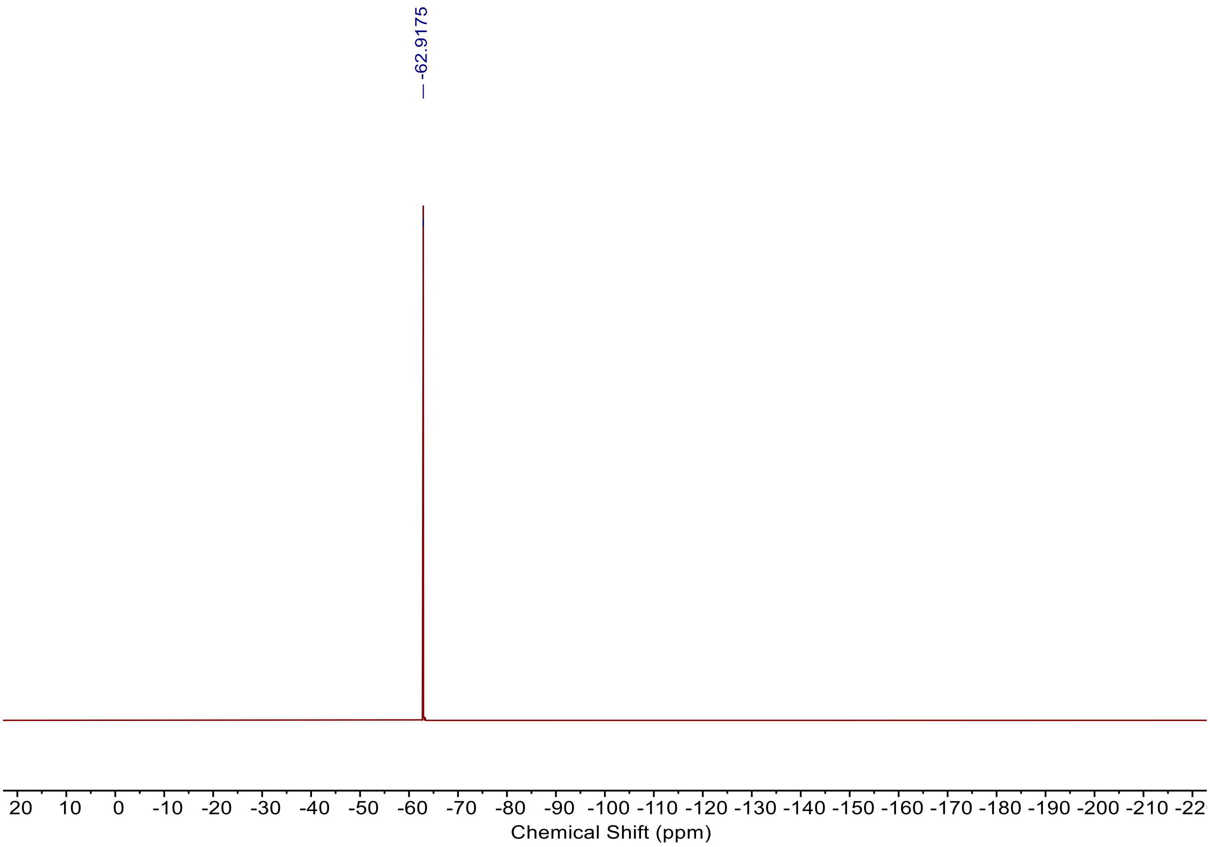

Figure S2. ^{13}F NMR spectrum of **15** in $CDCl_3$, 565 MHz, 298K.

XB-C_{60}-DB18C6 adduct **1**

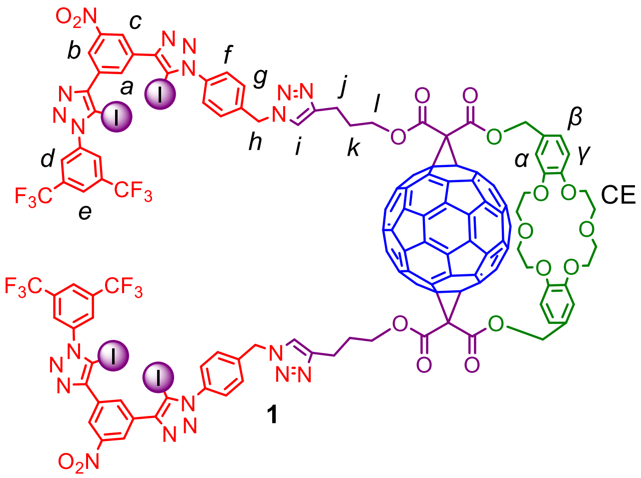

[$Cu(CH_3CN)_4$]PF_6 (5.2 mg, 14 µmol) and TBTA (7.9 mg, 14 µmol) were dissolved in DCM (anhydrous, 2 mL) inside a thoroughly dried reaction vessel. The mixture was stirred for 30 min under N_2 atmosphere. Next, azide **15** (26 mg, 31 µmol) and fullerene adduct **9** (20 mg, 14 µmol) were added. The reaction mixture was left stirring under N_2 atmosphere for 2 days in room temperature. Then, it was diluted with DCM (15 mL), washed with aqueous EDTA (10%)/NH_3 (2%) solution (3x10 mL), and brine (10 mL). Organic layers were combined and dried over $MgSO_4$. The solvent was removed in vacuo to give the crude product. Purification by flash chromatography (SiO_2, 3% MeOH/DCM) yielded product **1** contaminated with TBTA. Purification by size exclusion chromatography (Bio-Beads, $CHCl_3$) afforded **1** which was then dissolved in DCM and washed with water to remove potential contamination of K^+ salts. In vacuo drying resulted in **1** as a dark brown solid (15.6 mg, 36%).

^{1}H NMR (600 MHz, $CDCl_3$) δ: 9.07 (bt, 2H, *a*), 8.99 (bt, 2H, *b/c*), 8.96 (bt, 2H, *b/c*), 8.16 (bs, 4H *d*), 8.13 (bs. 2H, *e*), 7.63-7.59 (m, 4H, *f*), 7.52 (s, 2H, *i*), 7.52-7.48 (m, 4H, *g*), 7.05 (bd, J = 7.5 Hz, 2H), 6.91 (bd, 2H), 6.63 (bd, J = 7.5 Hz, 2H), 5.75 (d, J = 10.8 Hz, 2H), 5.71 (s, 4H. *h*), 5.41 (d, J = 10.8 Hz, 2H), 4.62 (m, 4H), 4.18-4.11 (m, 2H), 4.10-3.95 (8H), 3.87- 3.70 (6H), 3.04 (t, J = 7.5 Hz, 4H), 2.38 (t, J = 7.5 Hz, 4H).

^{13}C NMR (151 MHz, $CDCl_3$) δ: 164.49, 164.44, 149.49, 149.02, 148.87, 148.24, 148.00, 147.49, 145.32, 145.21, 145.17, 144.79, 144.66, 144.54, 144.46, 144.39, 143.84, 143.75, 143.67, 143.60, 143.40, 143.29, 143.26, 143.16, 142.84, 142.53, 142.01, 141.09, 140.65, 140.46, 140.30, 138.06, 137.87, 137.83, 137.24, 136.86, 133.53 (q), 132.57, 131.83, 128.94, 127.81, 127.25, 126.95, 126.93, 124.48, 122.56, 122.55 (q), 122.31, 121.73, 121.67, 114.32, 112.24, 79.12, 78.72, 70.73, 70.18, 69.94, 69.91, 69.77, 69.73, 68.76, 68.11, 66.36, 53.46, 28.21, 22.16.

^{19}F NMR (565 MHz, $CDCl_3$) δ -62.87.

HR MS (ESI+ve) m/z: 3146.0816 found, ([M+]$^+$, $C_{148}H_{64}F_{12}I_4N_{20}O_{18}$ calculated 3146.0799)

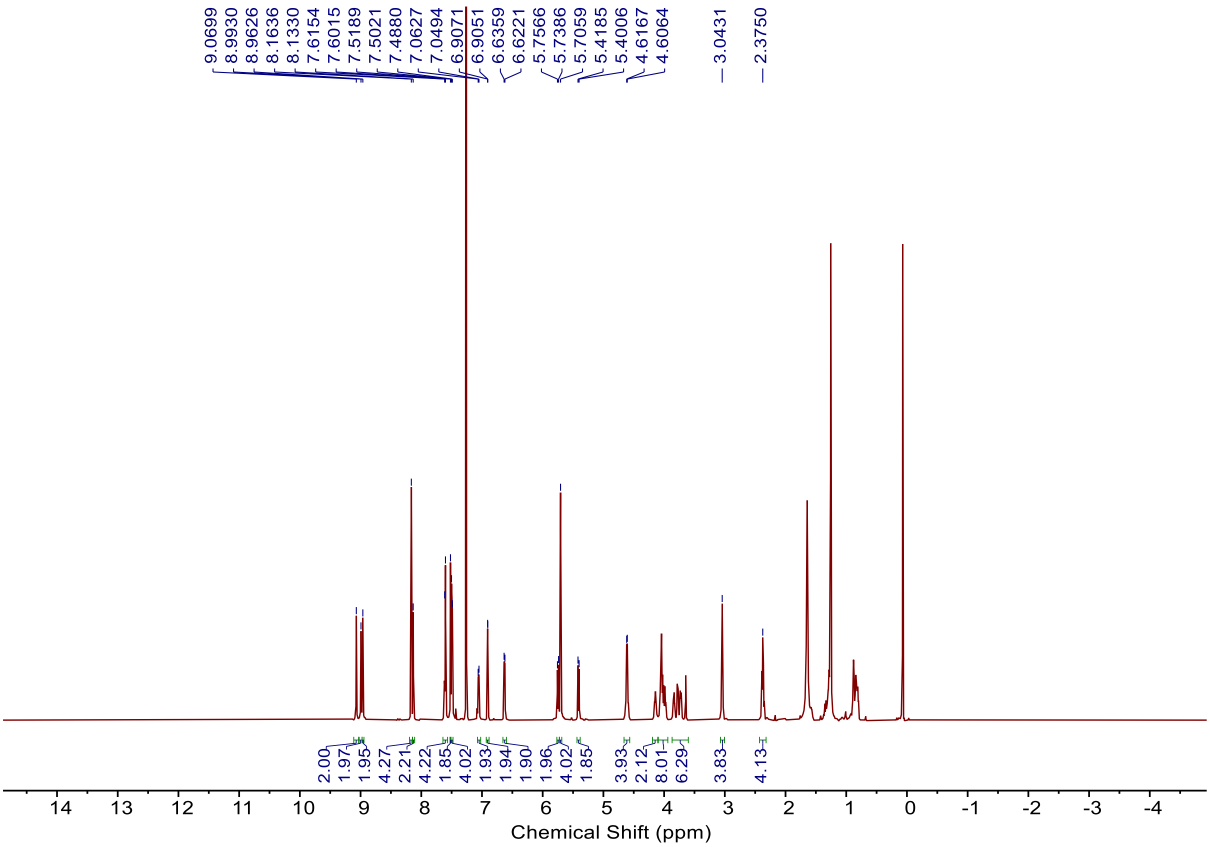

Figure S3. ^{1}H NMR spectrum of **1** in $CDCl_3$, 500 MHz, 298K.

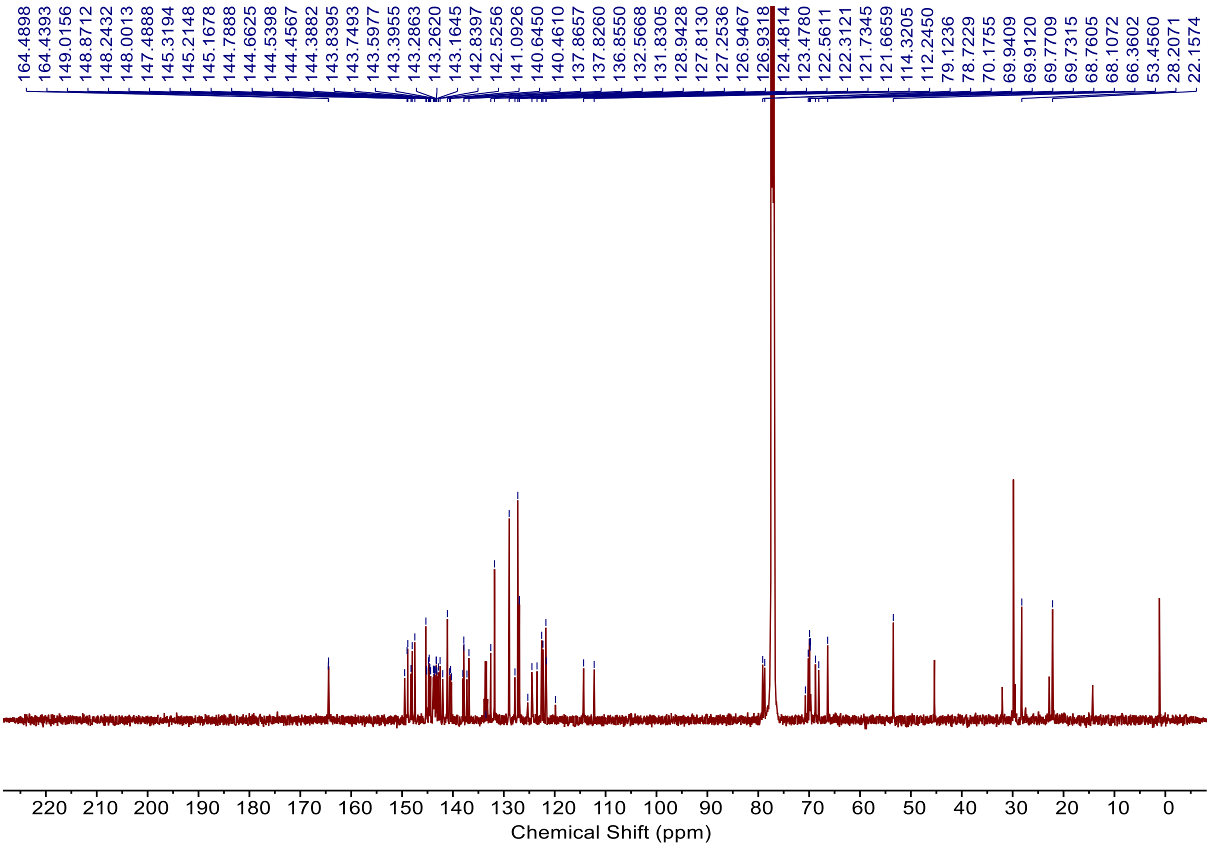

Figure S4. ^{13}C NMR spectrum of **1** in $CDCl_3$, 126 MHz, 298K.

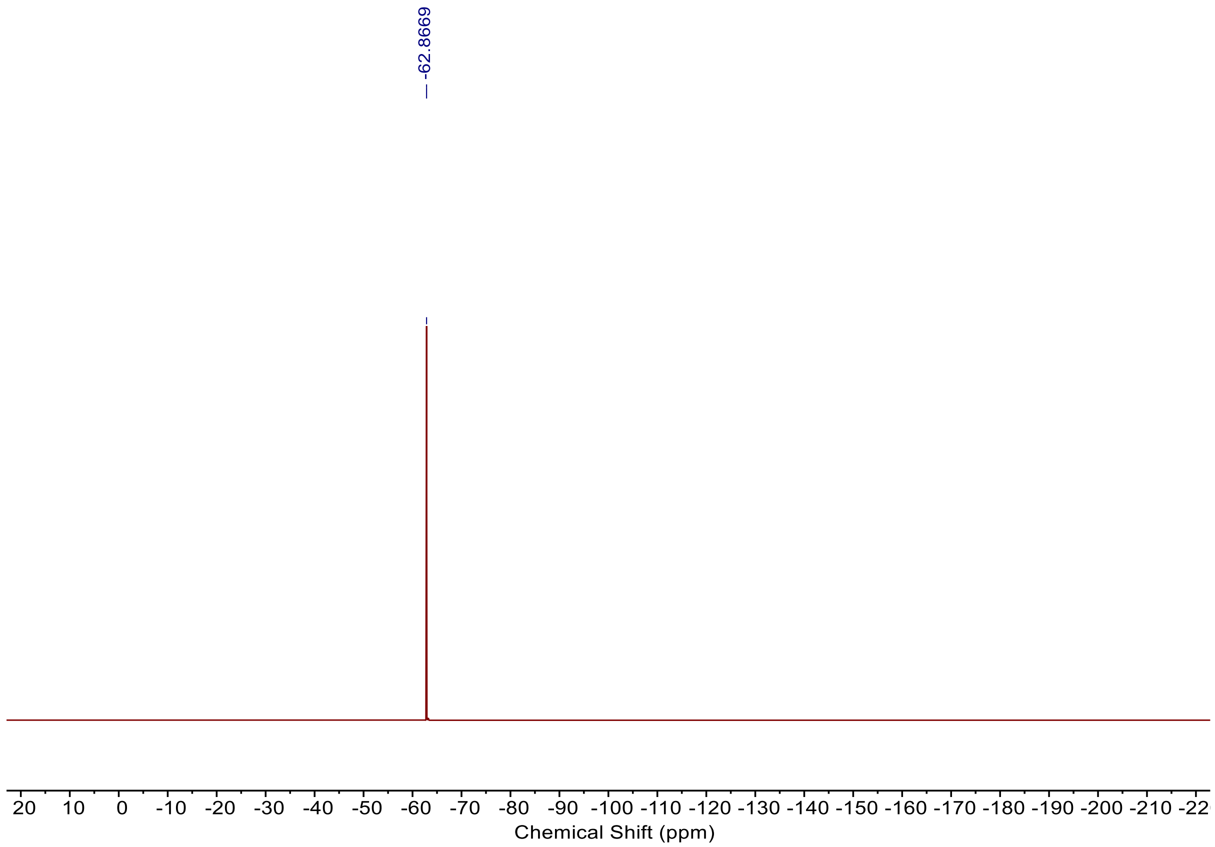

Figure S5. ^{13}F NMR spectrum of **1** in $CDCl_3$, 565 MHz, 298K.

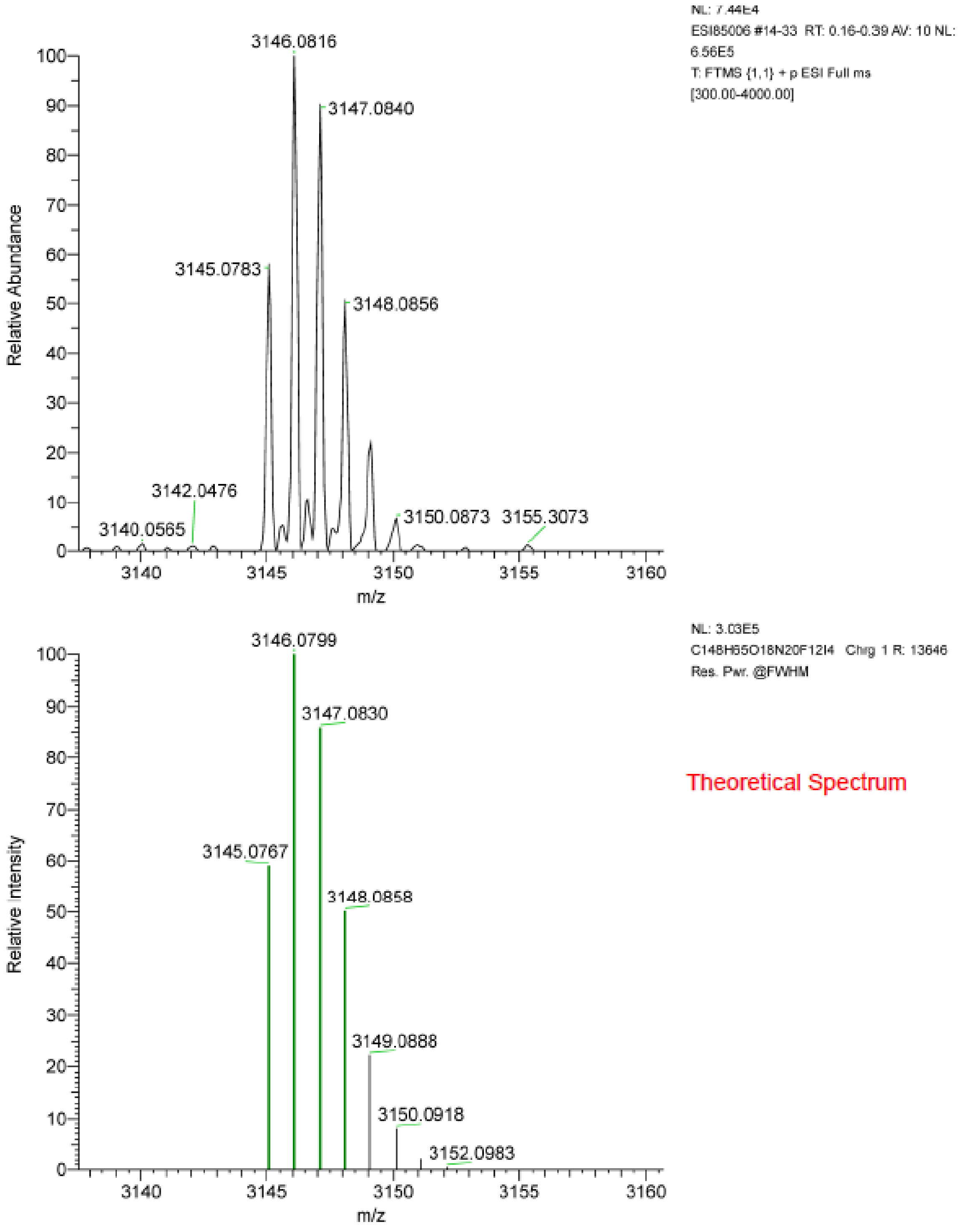

Figure S6. Results of HR-ESI-MS of **1**

Mono malonate **19**

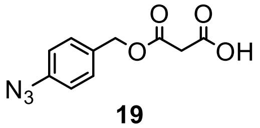

19

To a thoroughly dried reaction vessel, equipped with a reflux condenser, Meldrum's acid (0.387 g, 2.68 mmol) and 4-azidobenzylalcohol (0.400 g, 2.68 mmol) were added followed by 10 mL of toluene (SPS). The solution was refluxed for 2 h. The mixture was left to cool down and then was washed with saturated $NaHCO_3$ solution (3x30 mL). Aqueous layer was then acidified using HCl (1 M) to a pH < 3 (release of CO_2) and extracted with DCM (3x30 mL). Organic layers were combined and solvent was removed on a rotavap to obtain **19** as a colourless liquid (0.535 g, 84%).
^{1}H NMR (500 MHz, $CDCl_3$) 7.38-7.34 (m, 2H), 7.05-7.01 (m, 2H), 5.19 (s. 2H), 3.48 (s, 2H).

Bis azide **17**

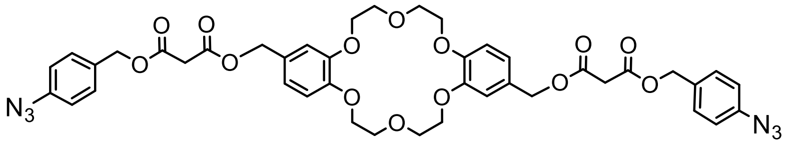

17

To a thoroughly dried reaction vessel diol **6** (134 mg, 0.32 mmol) and KPF_6 (59 mg, 0.319 mmol) were added followed by CH_3CN (7 ml). The mixture was sonicated for 5 min. DCM (27 ml) was added to the mixture followed by mono-malonate **19** (300 mg, 1.28 mmol) dissolved in DCM (1 mL) and DMAP (171 mg, 1.40 mmol). The reaction vessel was cooled down to 0°C and EDC·HCl (269 mg, 1.40 mmol) was added. The mixture was stirred in room temperature for 2 days under N_2. Then it was washed with a solution of citric acid (10%), solution of $NaHCO_3$ (saturated), H_2O, and brine. The organic layer was dried over $MgSO_4$ and solvents were removed on a rotavap. Purified by flash chromatography (SiO_2; DCM/MeOH 1%-3%). Final product was then dissolved in DCM and washed with water to remove K^+ salts. Solvents removal in vacuo afforded compound **8** as a white solid (149 mg; 55%).
^{1}H NMR (600 MHz, $CDCl_3$) δ: 7.31-7.28 (m, 4H), 6.99-6.95 (m, 4H), 6.89 – 6.83 (m, 4H), 6.80 (d, J = 8.1 Hz, 2H), 5.11 (s, 4H), 5.07 (s, 4H), 4.18 – 4.12 (m, 8H), 4.06 – 4.00 (m, 8H), 3.43 (s, 4H).
^{13}C NMR (151MHz, $CDCl_3$) δ: 166.37, 148.97, 148.68, 140.37, 132.08, 130.09, 128.25, 121.90, 119.28, 113.88, 113.01, 69.84, 68.82, 68.76, 67.43, 66.72, 41.70.
HR MS (ESI+ve) m/z: 855.2828 found, ($[M+H]^+$, $C_{42}H_{43}N_6O_{14}$ calculated 855.2832)

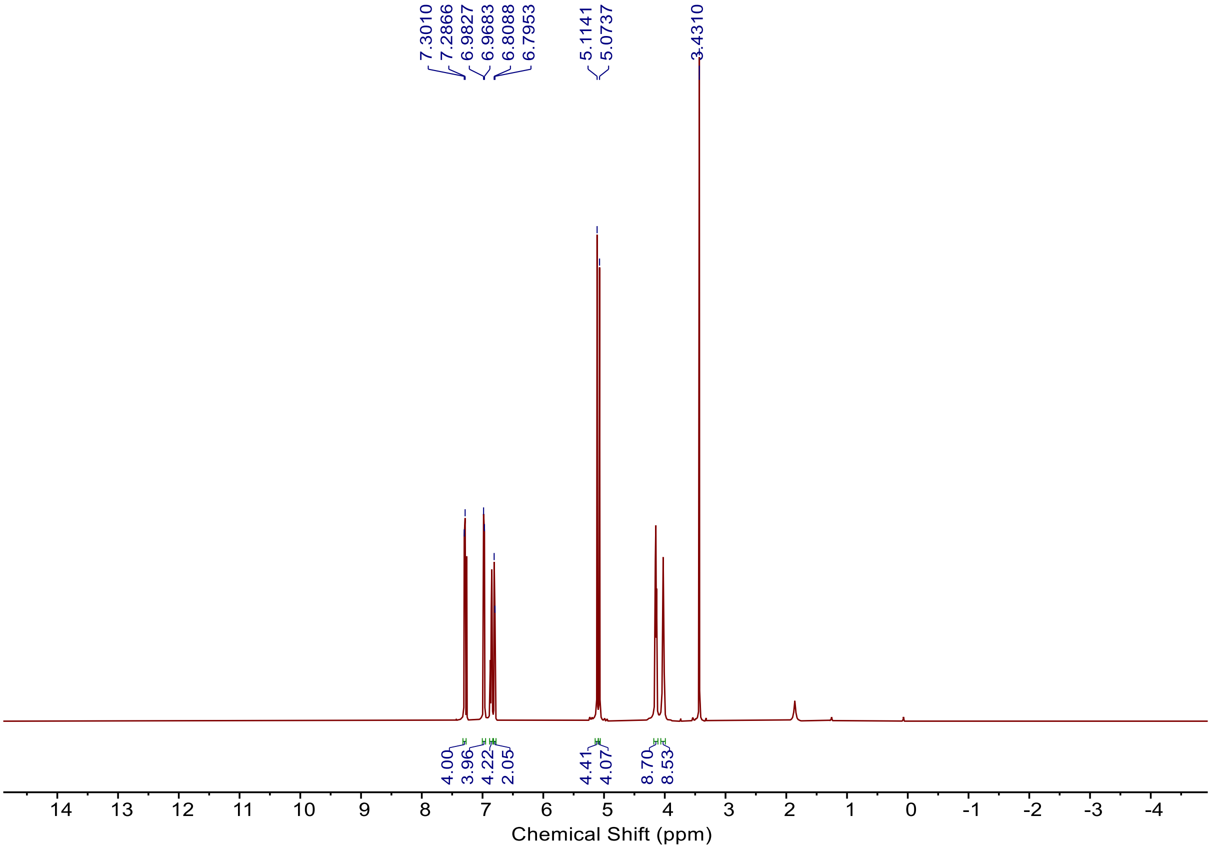

Figure S7. ^{1}H NMR spectrum of **17** in $CDCl_3$, 500 MHz, 298K.

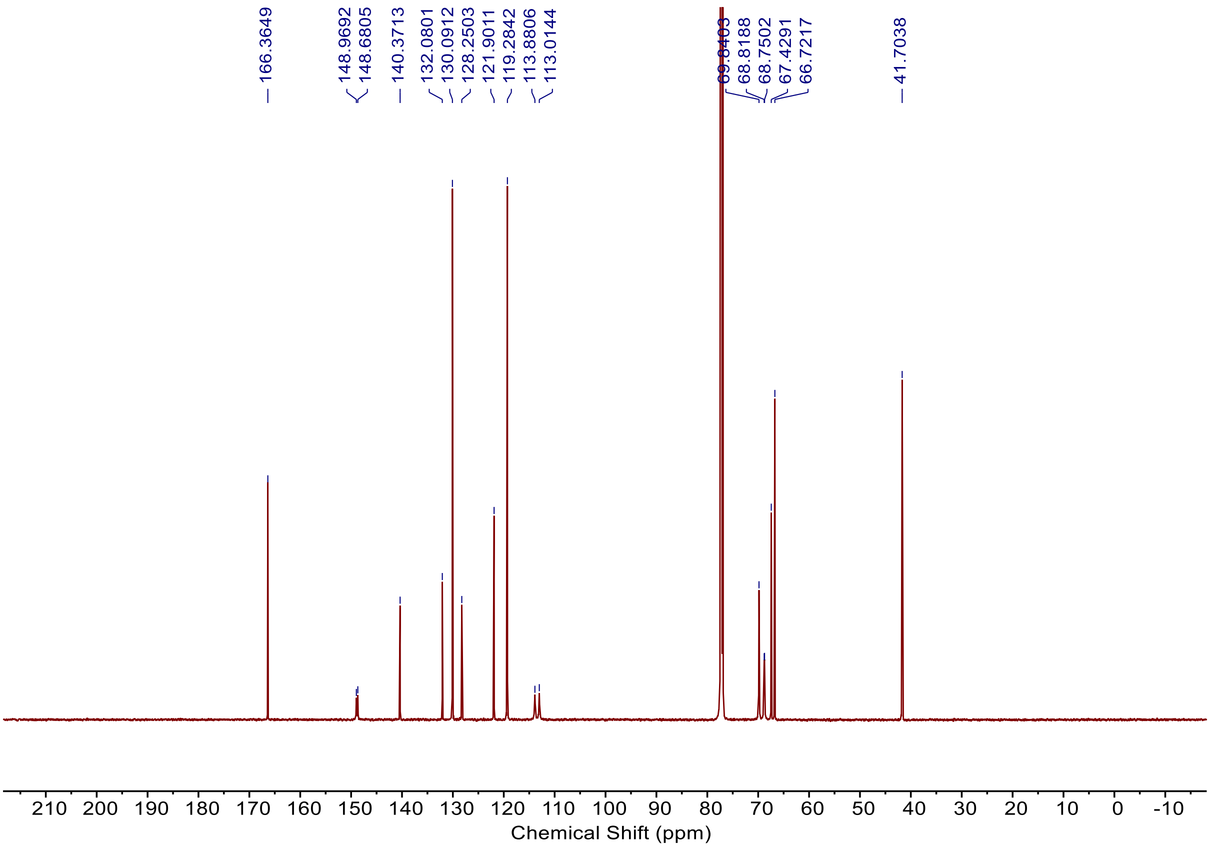

Figure S8. ^{13}C NMR spectrum of **17** in $CDCl_3$, 126 MHz, 298K.

XB-DB18C6 adduct **16**

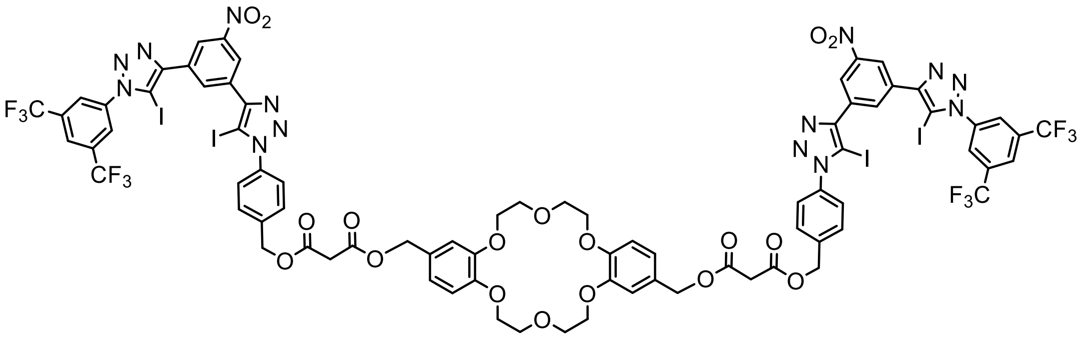

16

$[Cu(CH_3CN)_4]PF_6$ (27 mg, 0.07 mmol) and TBTA (37 mg, 0.07 mmol) were dissolved in DCM (anhydrous, 2 mL). The mixture was stirred for 30 min under N_2. Then iodoalkyne **12** (100 mg, 0.15 mmol) and bis-azide **17** (60 mg, 0.07 mmol) were added. The reaction mixture was protected from the light and stirred overnight under N_2. Then, it was diluted with DCM (15 mL) and washed with aqueous EDTA (10%)/NH_3 (2%) solution (3x10 mL), followed by brine (10 mL). Organic layers were combined and dried over $MgSO_4$. The solvent was removed in vacuo to give the crude product. Purification by flash chromatography (SiO_2, 2% EtOH/DCM) afforded product **16** as a white solid (57 mg, 37%).

^{1}H NMR (600 MHz, $CDCl_3$) δ 9.11 (t, J = 1.5 Hz, 2H, *a*), 9.03 (bt, J = 1.6 Hz, 2H, *b*), 8.98 (t, J = 1.6 Hz, 2H, *c*), 8.17 (bs, 4H, *d*), 8.13 (bs, 2H, *e*), 7.52 (m, 8H), 6.89 – 6.82 (m, 4H), 6.79 (d, J = 8.1 Hz, 2H), 5.28 (s, 4H), 5.10 (s, 4H), 4.16 – 4.08 (m, 8H), 4.03-3.96 (m, 8H), 3.51 (s, 4H).

^{13}C NMR (151 MHz, $CDCl_3$) δ: 166.29, 166.24, 149.06, 148.93, 147.91, 138.18, 137.90, 136.54, 133.54 (q), 132.71, 131.88, 131.74, 130.10, 128.88, 128.21, 126.94, 126.76, 124.34, 122.50, 122.46 (q), 122.29, 121.83, 119.86, 119.29, 69.81, 68.72, 67.47, 66.14, 53.57, 41.74.

^{19}F NMR (565 MHz, $CDCl_3$) δ -62.89.

HR MS (ESI+ve) m/z: 2232.9629 found, ($[M+Na]^+$, $C_{78}H_{54}F_{12}I_4N_{14}O_{18}Na$ calculated 2232.9620)

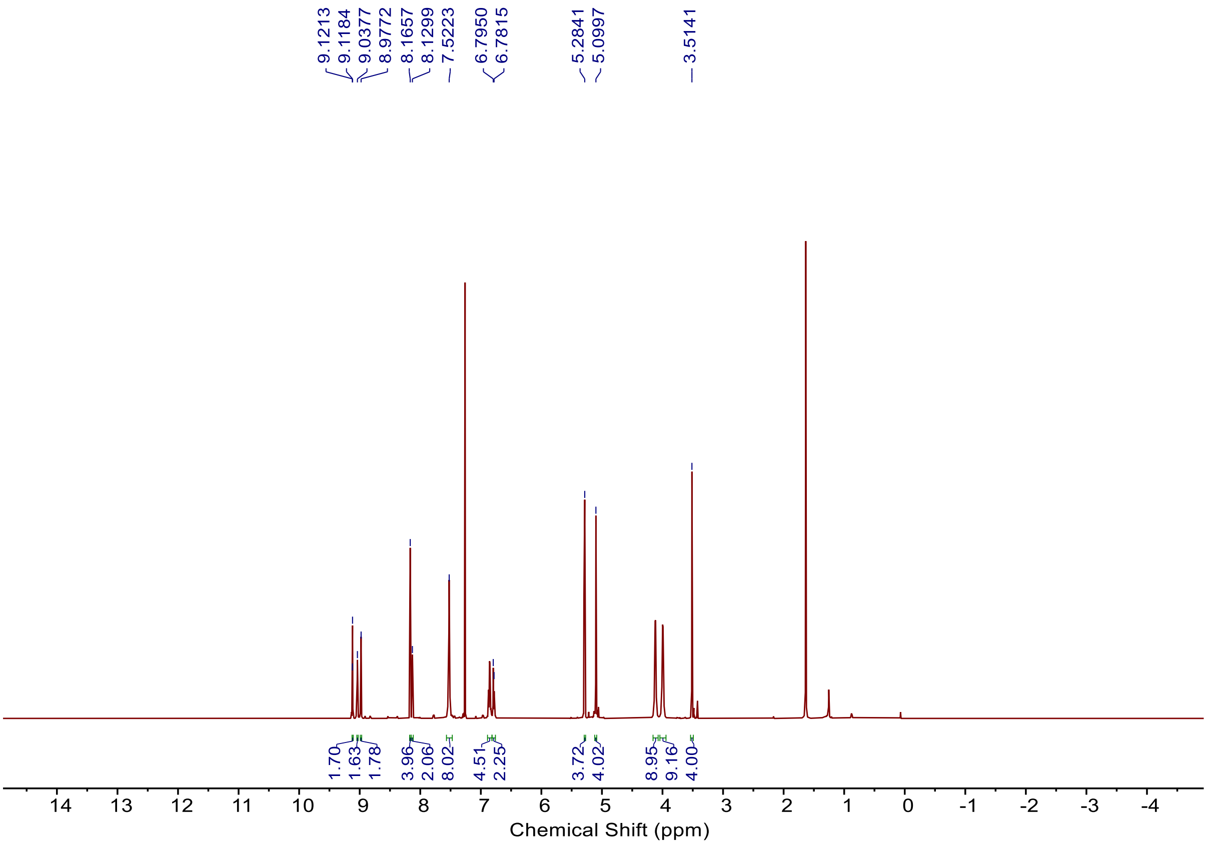

Figure S9. ^{1}H NMR spectrum of **17** in $CDCl_3$, 500 MHz, 298K.

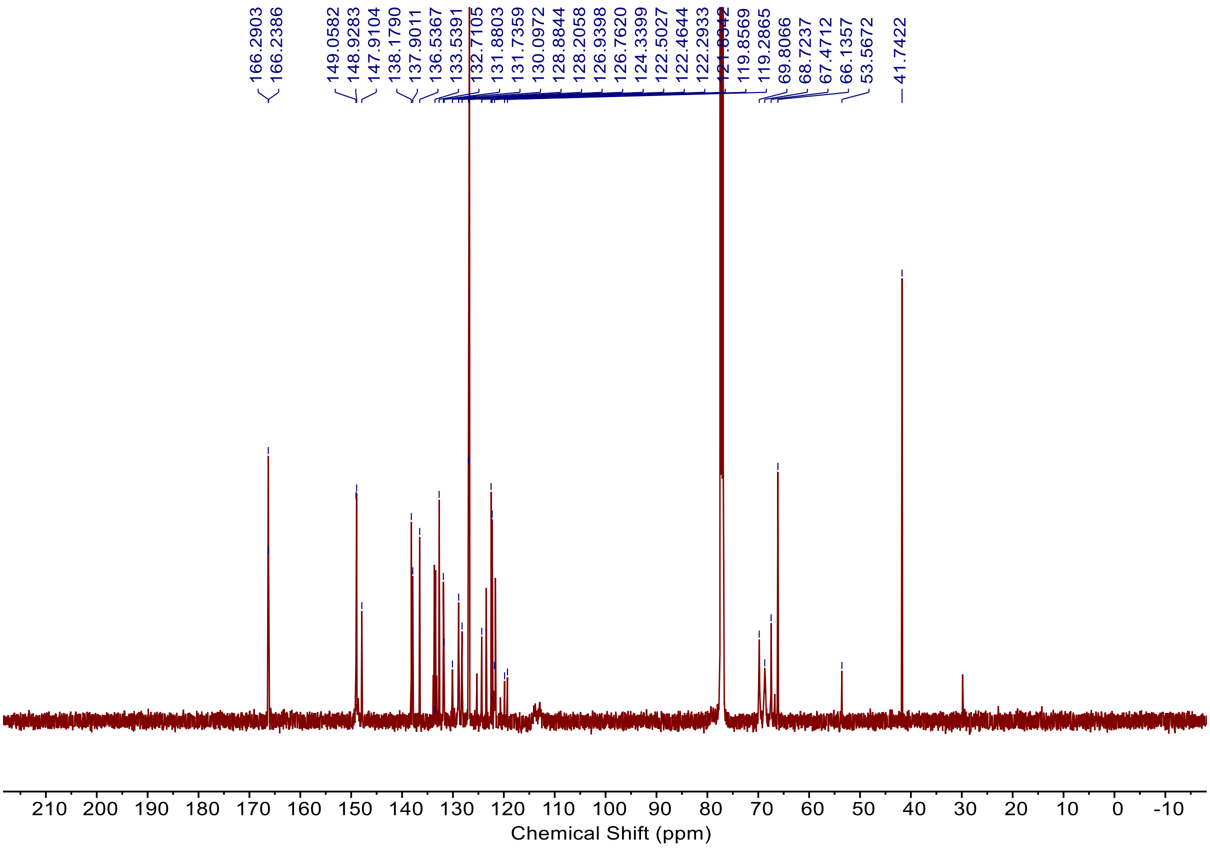

Figure S20. ^{13}C NMR spectrum of **17** in $CDCl_3$, 126 MHz, 298K.

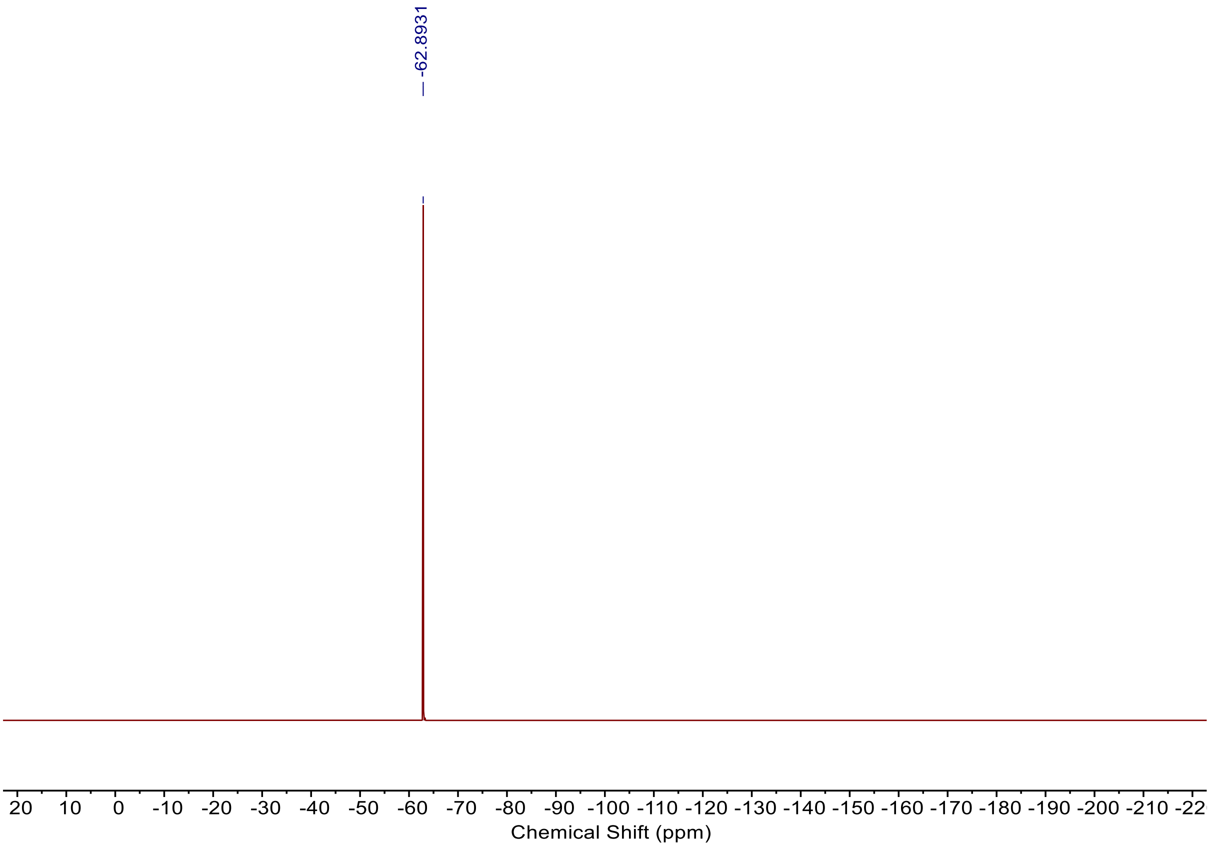

Figure S10. ^{13}F NMR spectrum of **1** in $CDCl_3$, 565 MHz, 298K.

<u>XB-C_{60}-DB18C6 adduct **2**</u>

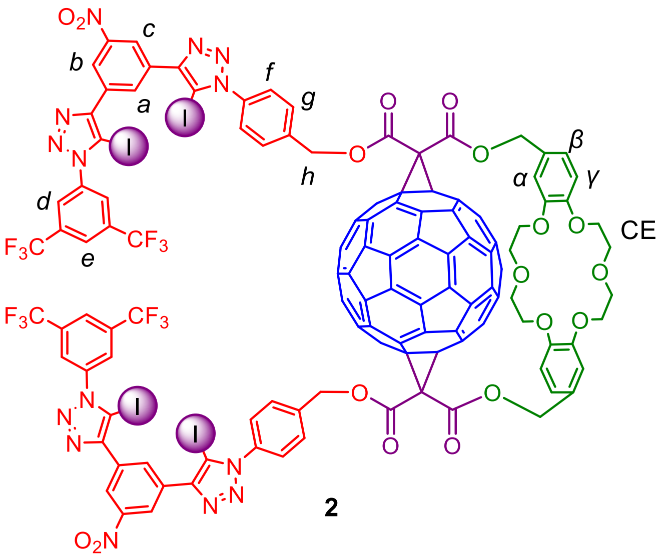

2

To a thoroughly dried reaction vessel C_{60} (60 mg, 0.083 mmol) and toluene (120 mL) were added. The solution was sonicated for 10 min and stirred for at least 30 min to solubilise fullerene. Bis-azide **17** (71 mg, 0.083 mmol) was added followed by KPF_6 (153 mg, 0.83 mmol) dissolved in acetonitrile (2 ml), and solid I_2 (42 mg, 0.167 mmol). Next, DBU (76 mg, 0.50 mmol) in acetonitrile (1 mL) was added dropwise and the mixture was left stirring for 1.5 h in room temperature. The mixture was then washed with water. Organic layers were combined and dried over $MgSO_4$. Solvents were removed and crude

was adsorbed on silica. Purification by flash chromatography (5 g of silica gel, toluene to obtain unreacted C_{60}, then AcOEt/toluene 1:1) afforded the product which was concentrated to ca. 0.5 mL. This solution was used immediately in the next step. Drying the crude and product was avoided at each step.

$[Cu(CH_3CN)_4]PF_6$ (31 mg, 0.083 mmol) and TBTA (44 mg, 0.083 mmol) were dissolved in DCM (anhydrous, 2 mL). The mixture was stirred for 30 min under N_2. Then iodoalkyne **12** (113 mg, 0.17 mmol) and bis-azide C_{60} solution obtained in a first step were added. The reaction mixture was protected from the light and stirred overnight under N_2. Then, it was diluted with DCM (15 mL) and washed with aqueous EDTA (10%)/NH_3 (2%) solution (3x10 mL), followed by brine (10 mL). Organic layers were combined and dried over $MgSO_4$. The solvent was removed in vacuo to give the crude product. Purification by flash chromatography (SiO_2, 2% MeOH/DCM), followed by size exclusion chromatography (Bio-Beads, $CHCl_3$) afforded **2** which was then dissolved in DCM and washed with water to remove potential contamination of K^+ salts. In vacuo drying resulted in **2** as a dark brown solid (33 mg, 14%).

^{1}H NMR (600 MHz, $CDCl_3$) δ 9.10 (t, J = 1.7 Hz, 2H, *a*), 9.02 (t, J = 1.7, 2H, *b*/*c*), 8.97 (t, J =1.7, 2H, *b*/*c*), 8.17 (bs, 4H, *d*), 8.14 (bs, 2H, *e*), 7.85 (d, J = 8.2 Hz, 4H, *g*), 7.70 (d, J = 8.2 Hz, 4H, *f*), 7.09 (dd, J = 8.3, 2.0 Hz, 2H, *β*), 6.94 (d, J = 2.0 Hz, 2H, α), 6.64 (d, J = 8.3 Hz, 2H, *γ*), 5.81 (d, J = 10.8 Hz, 4H), 5.75 (m, 4H, *h*), 5.45 (d, J = 10.8 Hz, 4H), 4.16 (td, J = 9.4, 3.4 Hz, 2H), 4.11 – 3.95 (m, 8H), 3.87 (td, J = 9.2, 3.6 Hz, 2H), 3.80 – 3.75 (m, 2H), 3.74 – 3.67 (m, 2H).

^{13}C NMR (151 MHz, $CDCl_3$) δ: 164.47, 164.34, 149.47, 148.95, 148.88, 148.24, 147.84, 145.34, 145.25, 144.90, 144.78, 144.66, 144.48, 144.41, 143.92, 143.79, 143.71, 143.63, 143.45, 143.39, 143.34, 143.29, 142.68, 142.37, 141.88, 141.20, 141.19, 141.16, 140.70, 140.56, 140.40, 138.21, 137.86, 137.62, 137.36, 137.00, 133.55 (q), 132.59, 131.80, 131.76, 129.83, 127.75, 127.07, 126.98, 126.96, 124.47, 124.38, 122.44, 122.43 (q), 122.18, 114.24, 112.03, 79.07, 78.48, 70.14, 69.92, 69.87, 69.76, 69.70, 68.54, 68.12, 67.96, 45.09.

^{19}F NMR (565 MHz, $CDCl_3$) δ -62.94.

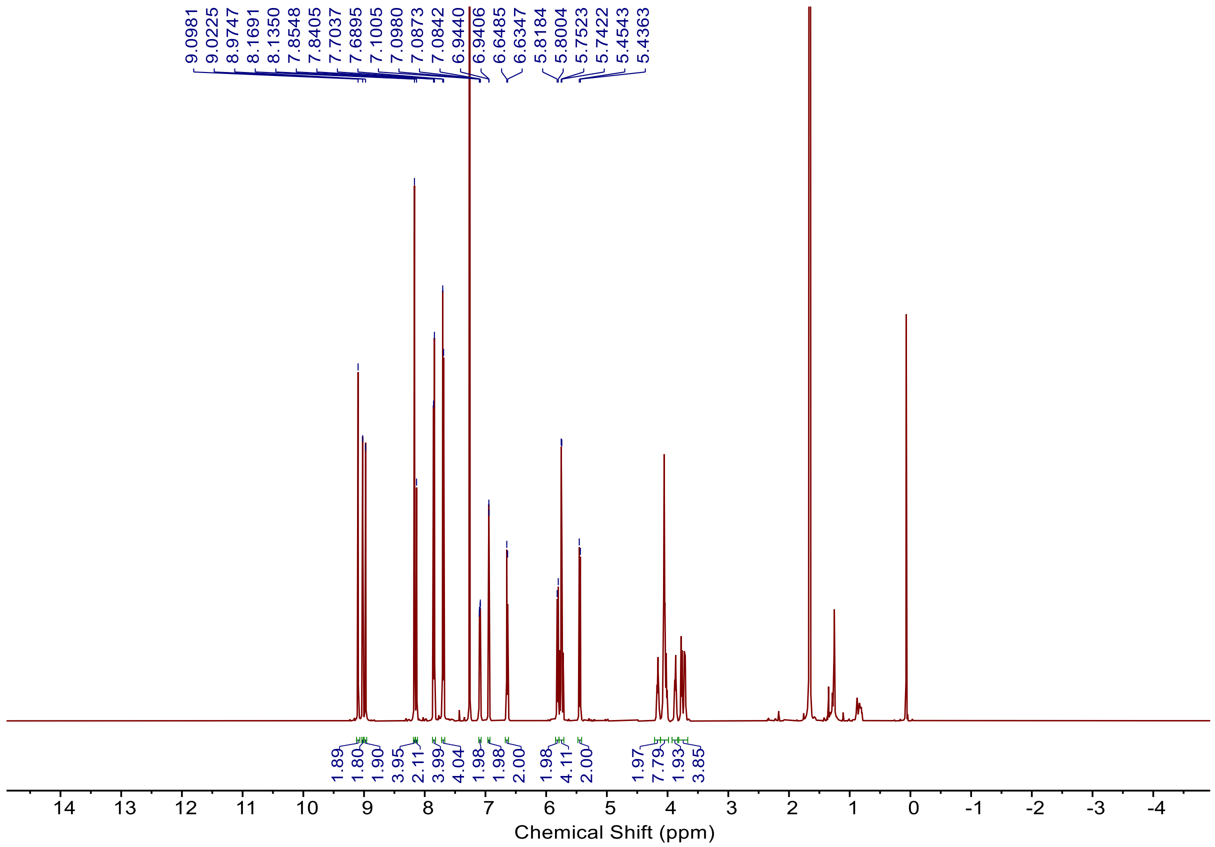

Figure S11. ^{1}H NMR spectrum of **2** in $CDCl_3$, 500 MHz, 298K.

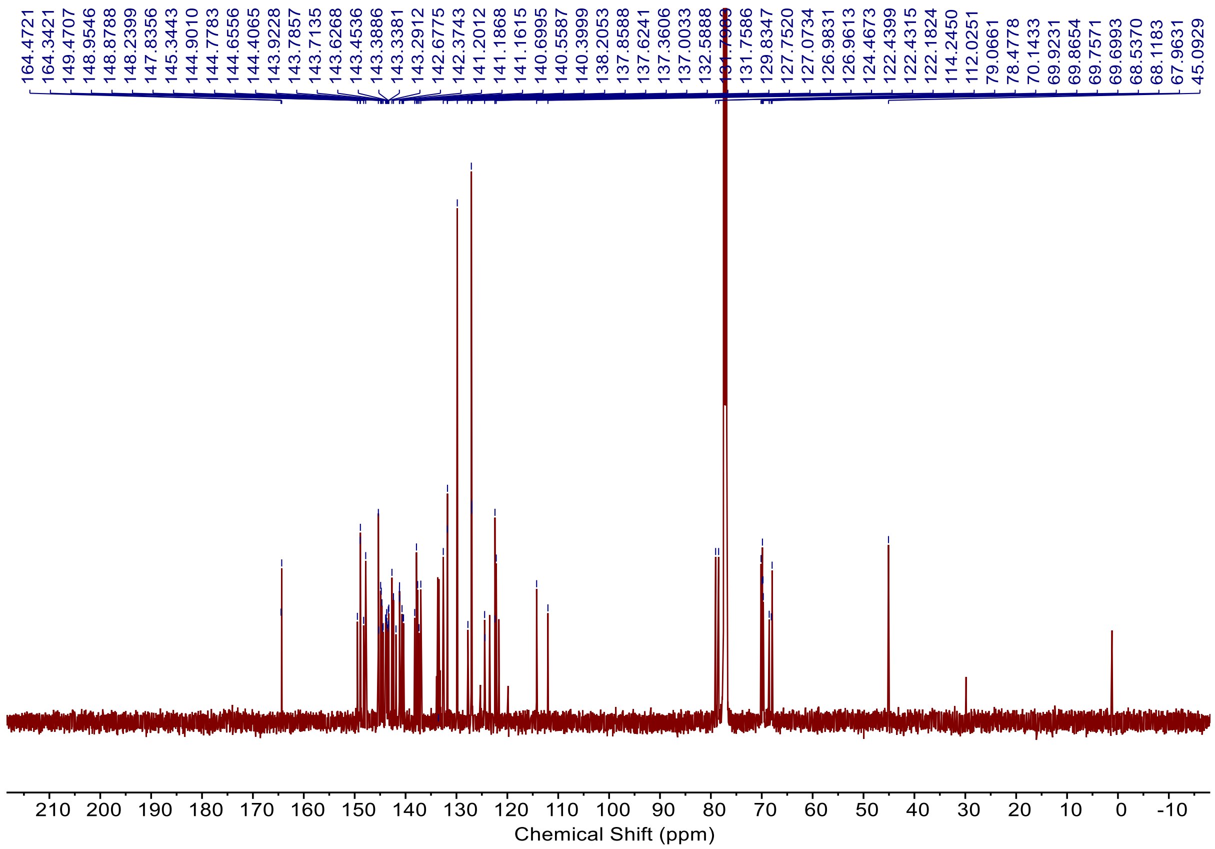

Figure S12. ^{13}C NMR spectrum of **2** in $CDCl_3$, 126 MHz, 298K.

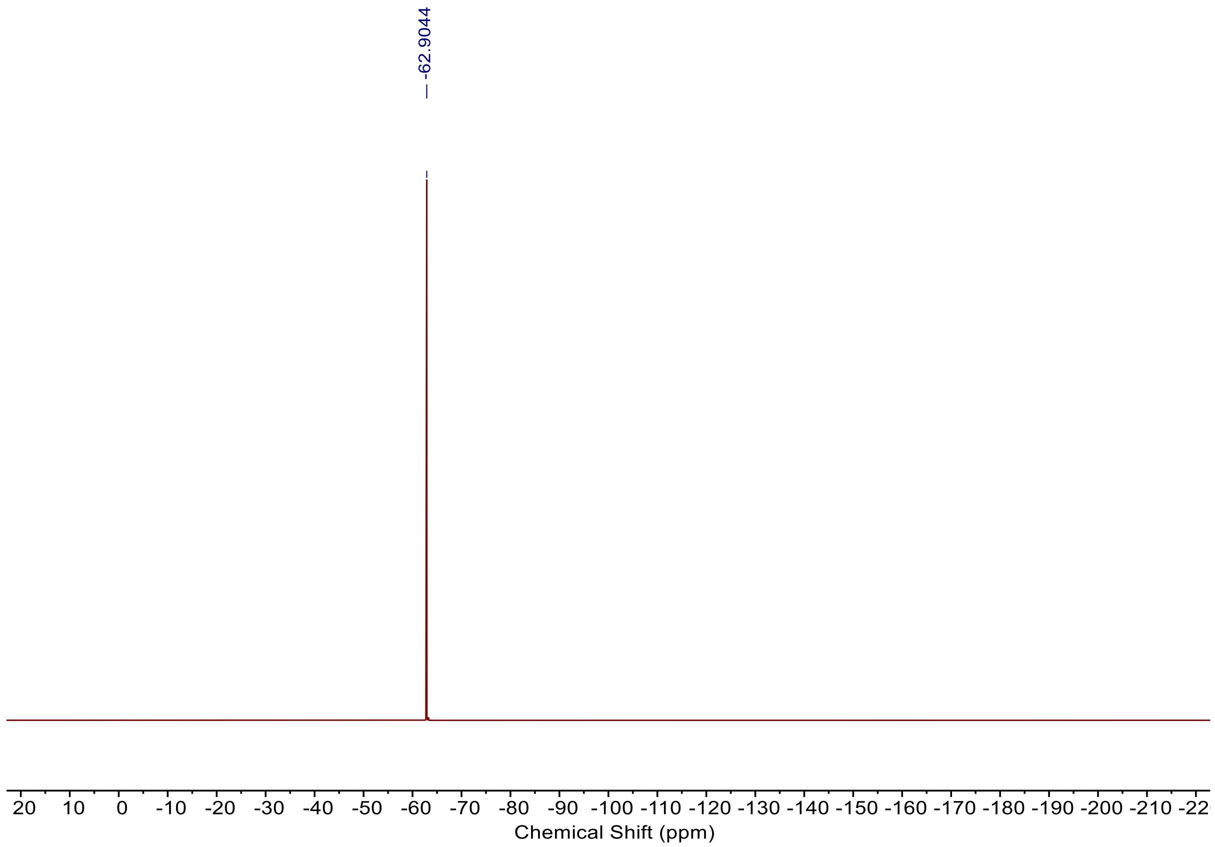

Figure S24. ^{13}F NMR spectrum of **2** in $CDCl_3$, 565 MHz, 298K.

S2 Anion Binding Studies by ^{1}H NMR

S2.1 Procedure

In a typical experiment a 25 mM solution of the chosen TBA salt of an anion (TBAX, X = I^-, Br^-, Cl^- or NO_3^-) was added to 0.5 mL solution containing 0.5 mM of a studied receptor in $CDCl_3$:CD_3CN 3:1 v/v. If a titration was conducted in presence of K^+: $KBAr^F_4$ was dissolved in $CDCl_3$:CD_3CN 1:1 v/v to concentration 1 mM and then diluted (1:1 v/v) with 1 mM of the receptor solution in $CDCl_3$ to obtain final concentrations of receptor (0.5 mM) and $KBAr^F_4$ (0.5 mM) in $CDCl_3$:CD_3CN 3:1 v/v

Typical titration isotherm comprises 17 data points, corresponding to 0.0, 0.2, 0.4, 0.6, 0.8, 1.0, 1.2, 1.4, 1.6, 1.8, 2, 2.5, 3.0, 4.0, 5.0, 7.0, 10.0 equivalents of added guest anion (100 µl of guest solution added by the last point). Addition of the solution containing the guest caused dilution of the host, which was accounted for in calculations. Binding constants were determined using BindFit, applying a 2:1 host-guest binding model in all cases.[7,8] At least two signals were simultaneously used to determine binding constants (global fitting approach – binding constant used a shared parameter).

S2.2 Titrations of Receptor 1

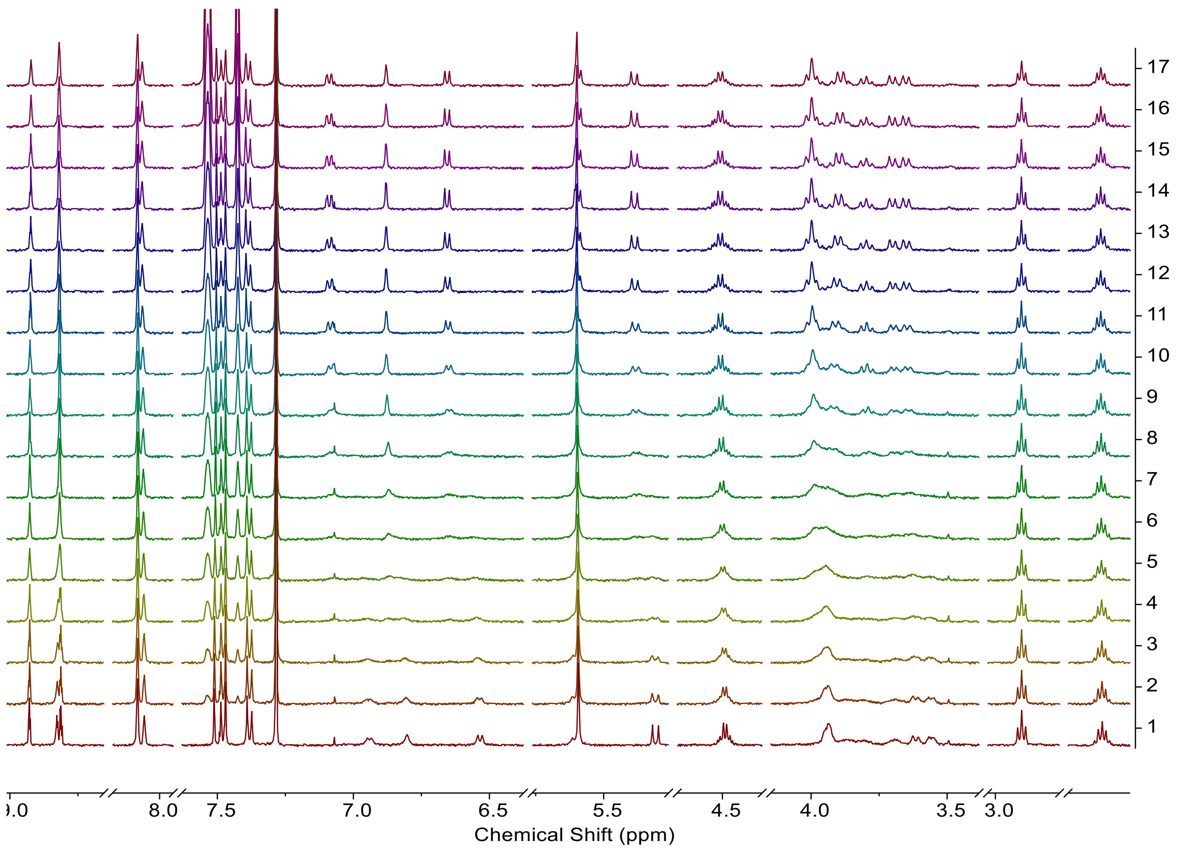

Figure S25. Truncated ^{1}H NMR titration spectra of **1** (0.5 mM) with KBArF in $CDCl_3$/CD_3CN (3:1 v/v), 500 MHz, 298 K. Spectrum no. 1 – 0 equivalents of the guest (KBArF); spectrum no. 17 – 10 equivalents of the guest (KBArF).

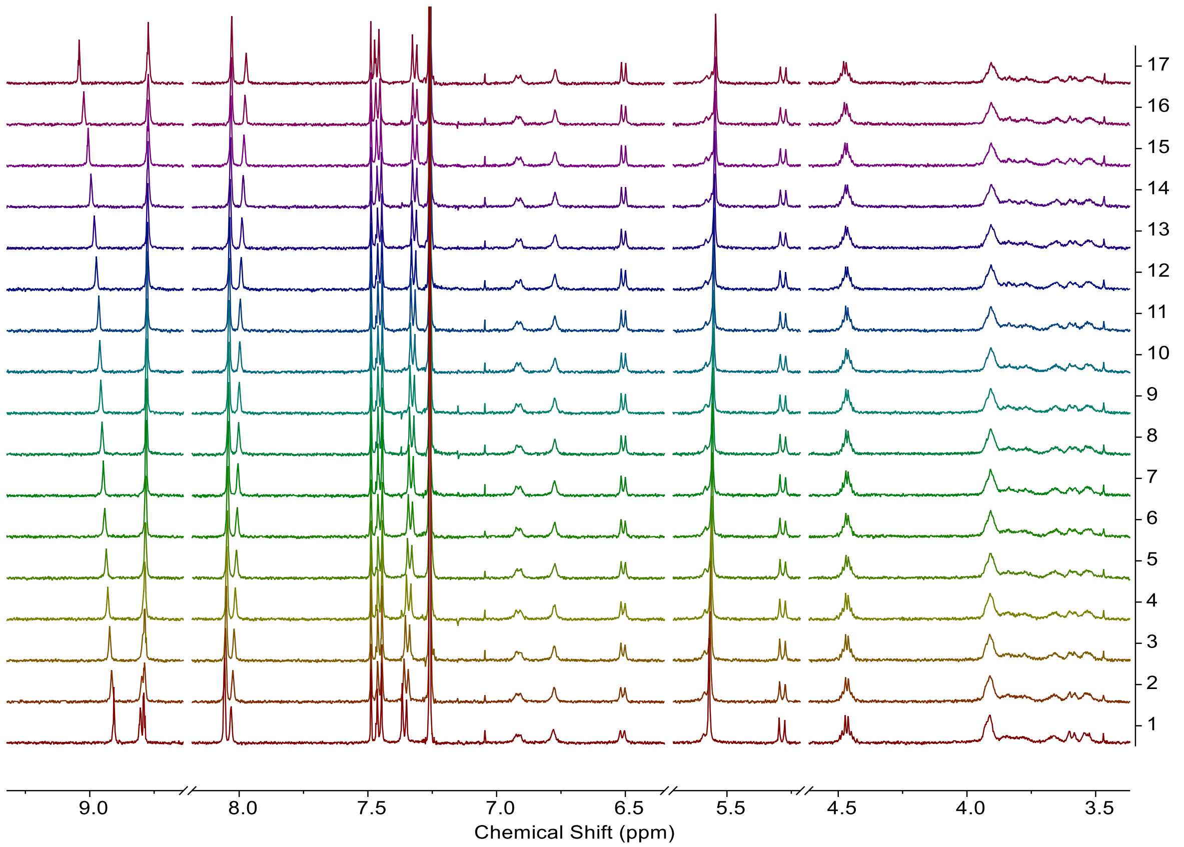

Figure S26. Truncated ^{1}H NMR titration spectra of **1** (0.5 mM) with TBACl in $CDCl_3/CD_3CN$ (3:1 v/v), 500 MHz, 298 K. Spectrum no. 1 – 0 equivalents of the guest (TBACl); spectrum no. 17 – 10 equivalents of the guest (TBACl).

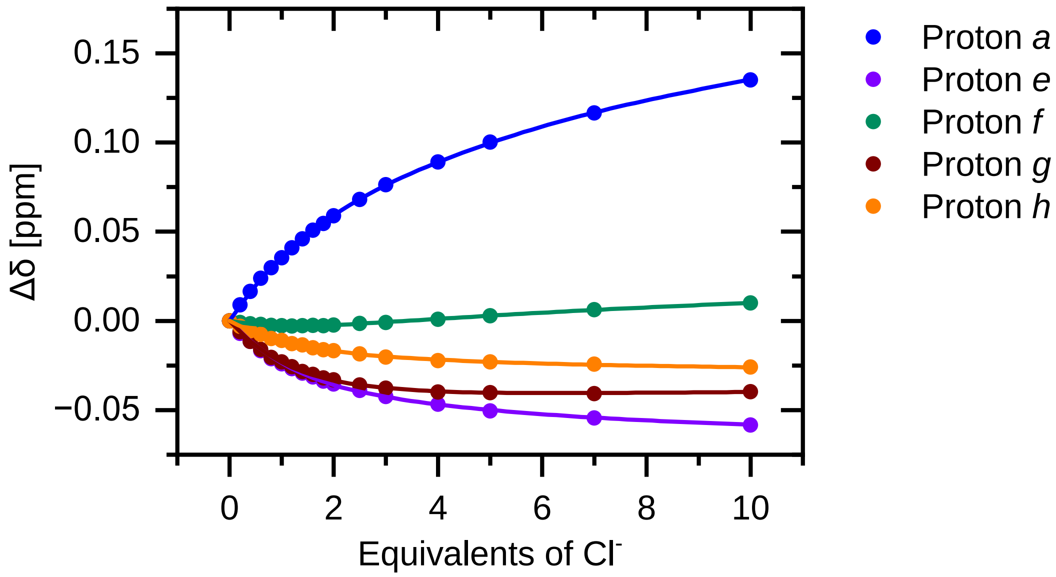

Figure S27. Examples of anion binding isotherms obtained during ^{1}H NMR titration of **1** (0.5 mM) with TBACl in $CDCl_3/CD_3CN$ (3:1 v/v). Circles represent experimental data and the lines represent the fitted isotherms obtained using Bindfit (Model 1:2).

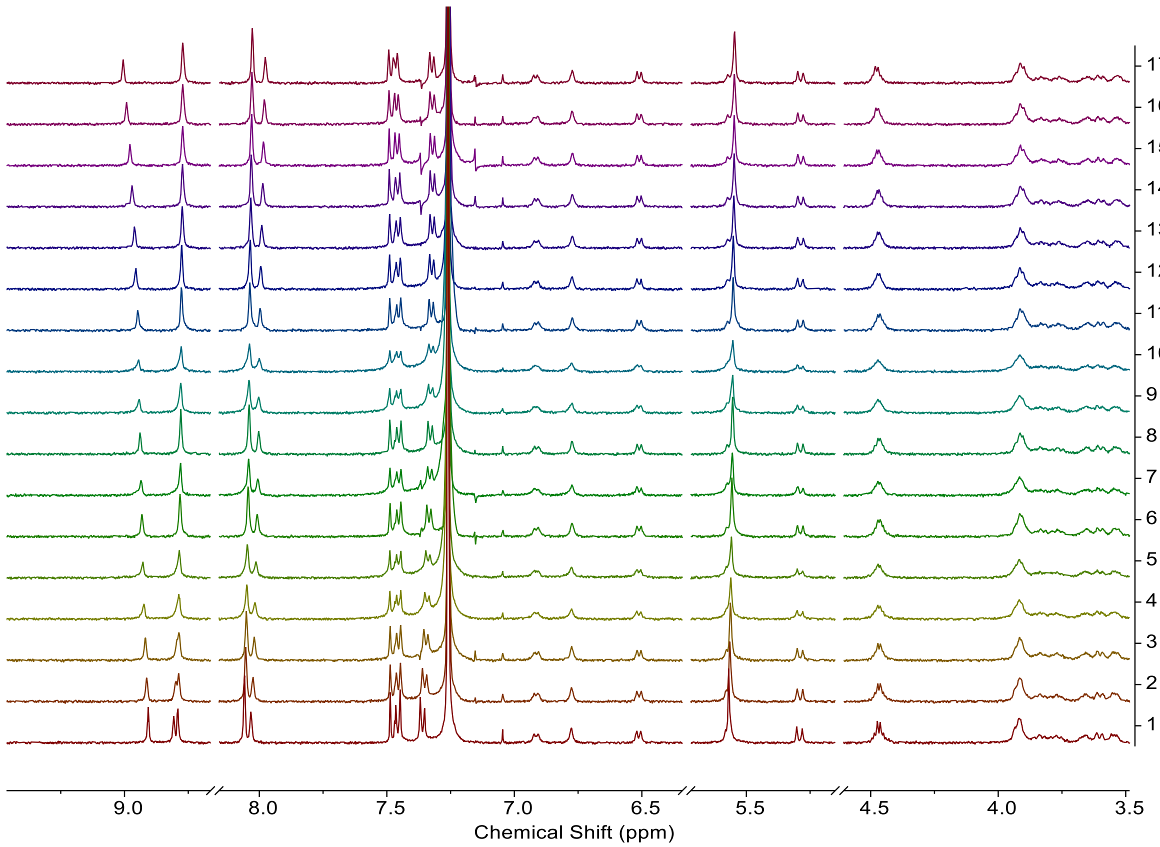

Figure S28. Truncated ^{1}H NMR titration spectra of **1** (0.5 mM) with TBABr in $CDCl_3/CD_3CN$ (3:1 v/v), 500 MHz, 298 K. Spectrum no. 1 – 0 equivalents of the guest (TBABr); spectrum no. 17 – 10 equivalents of the guest (TBABr).

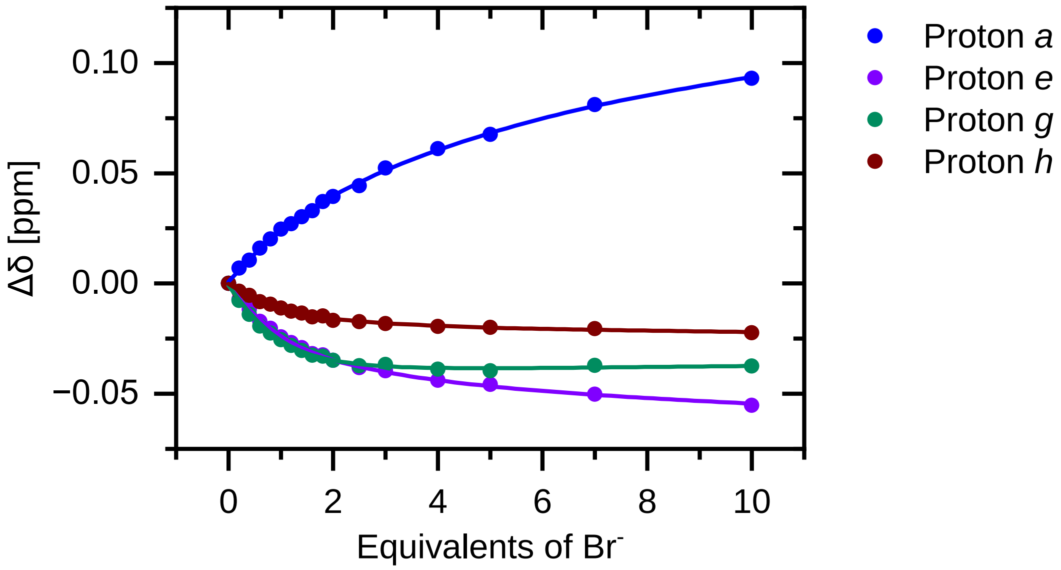

Figure S29. Examples of anion binding isotherms obtained during ^{1}H NMR titration of **1** (0.5 mM) with TBABr in $CDCl_3/CD_3CN$ (3:1 v/v). Circles represent experimental data and the lines represent the fitted isotherms obtained using Bindfit (Model 1:2).

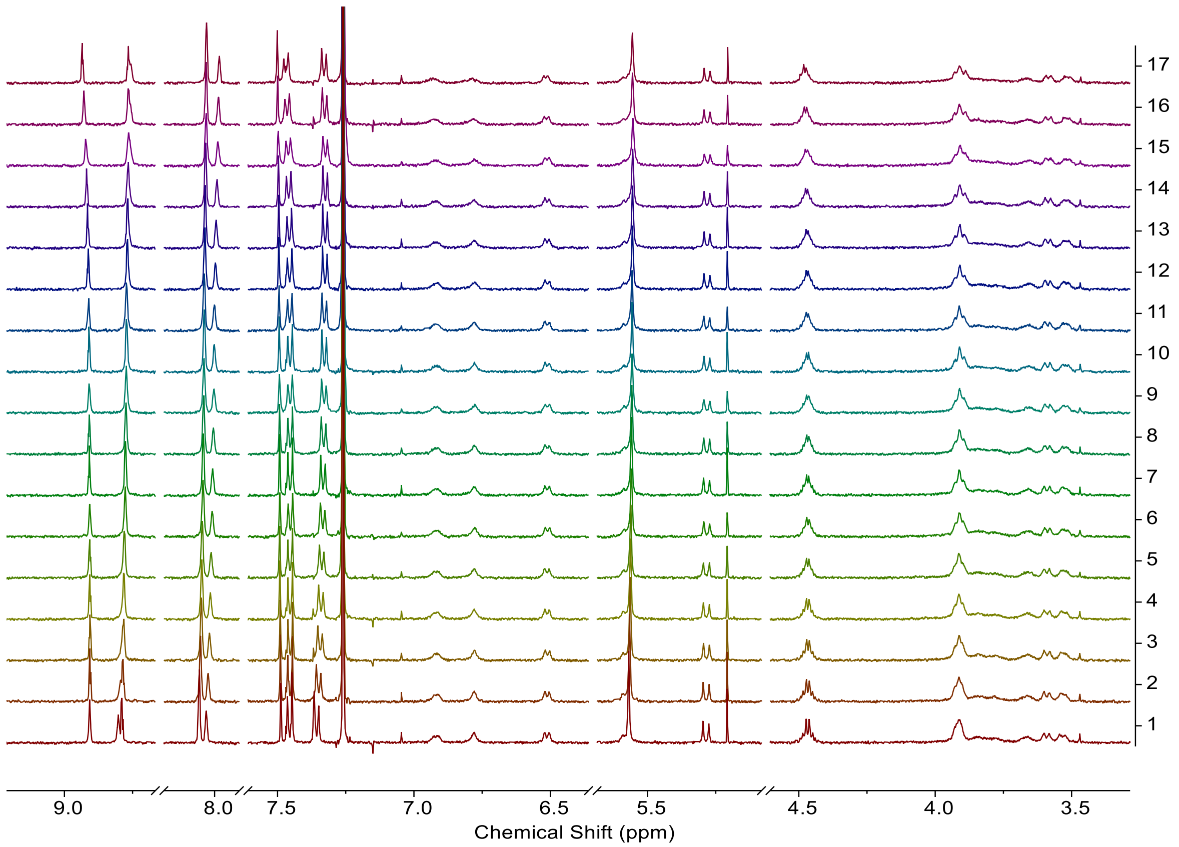

Figure S30. Truncated ^{1}H NMR titration spectra of **1** (0.5 mM) with TBAI in $CDCl_3/CD_3CN$ (3:1 v/v), 500 MHz, 298 K. Spectrum no. 1 – 0 equivalents of the guest (TBAI); spectrum no. 17 – 10 equivalents of the guest (TBAI).

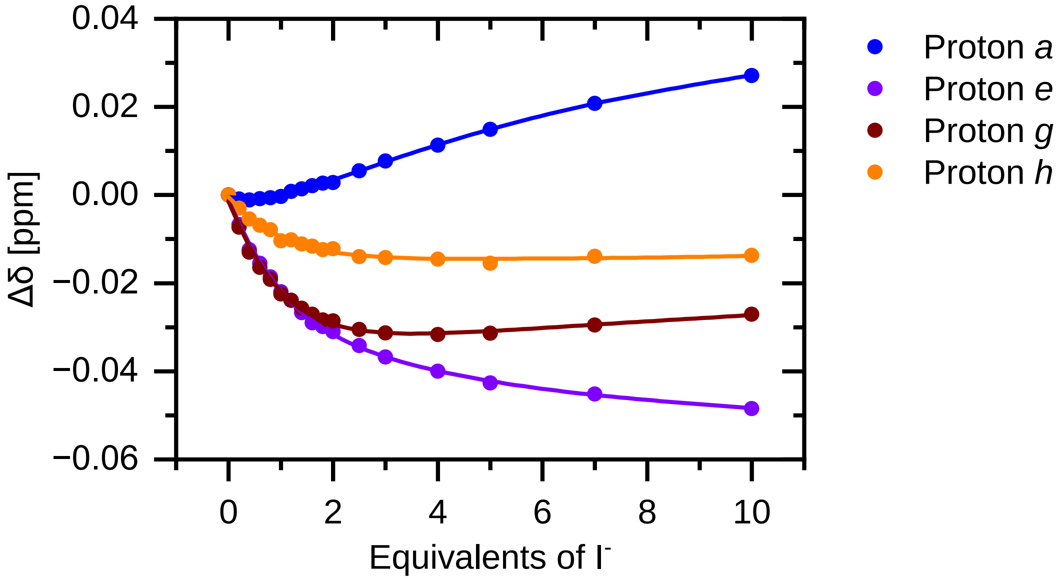

Figure S31. Examples of anion binding isotherms obtained during ^{1}H NMR titration of **1** (0.5 mM) with TBAI in $CDCl_3/CD_3CN$ (3:1 v/v). Circles represent experimental data and the lines represent the fitted isotherms obtained using Bindfit (Model 1:2).

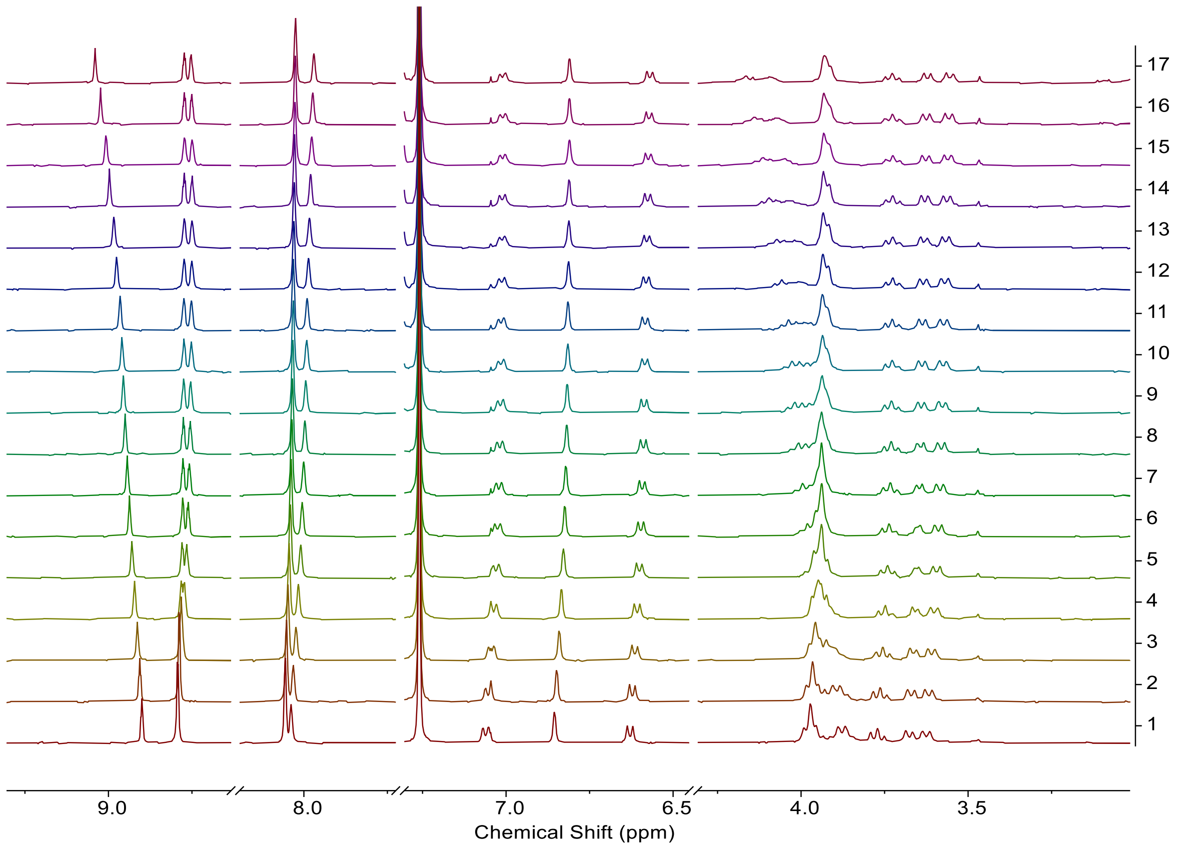

Figure S32. Truncated ^{1}H NMR titration spectra of **1** (0.5 mM) in presence of 1 equiv. KBArF (0.5 mM) with TBACl in $CDCl_3/CD_3CN$ (3:1 v/v), 500 MHz, 298 K. Spectrum no. 1 – 0 equivalents of the guest (TBACl); spectrum no. 17 – 10 equivalents of the guest (TBACl).

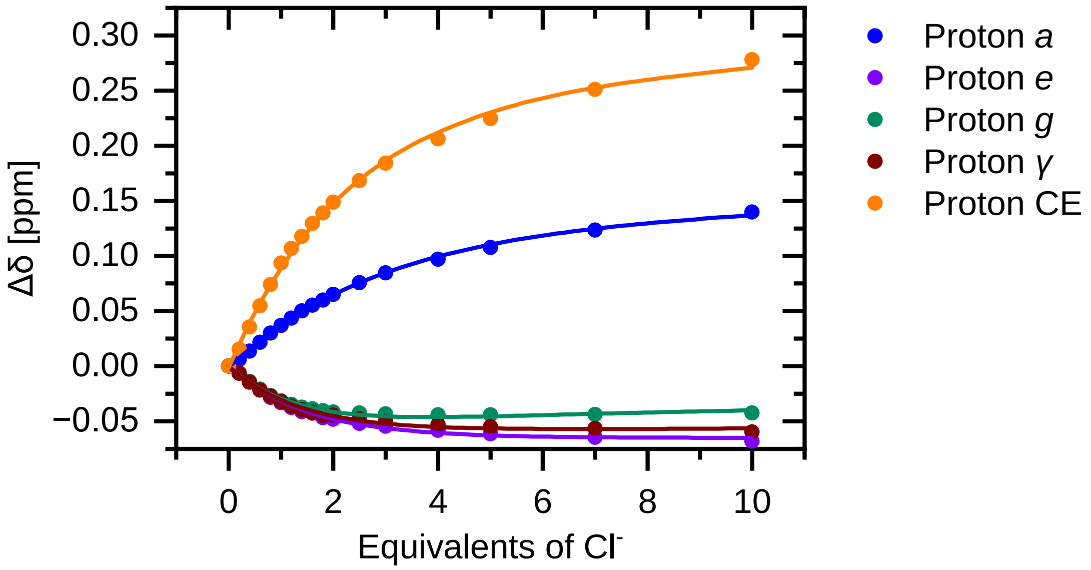

Figure S33. Examples of anion binding isotherms obtained during ^{1}H NMR titration of **1** (0.5 mM) in presence of 1 equiv. KBArF (0.5 mM) with TBACl in $CDCl_3/CD_3CN$ (3:1 v/v). Circles represent experimental data and the lines represent the fitted isotherms obtained using Bindfit (Model 1:2).

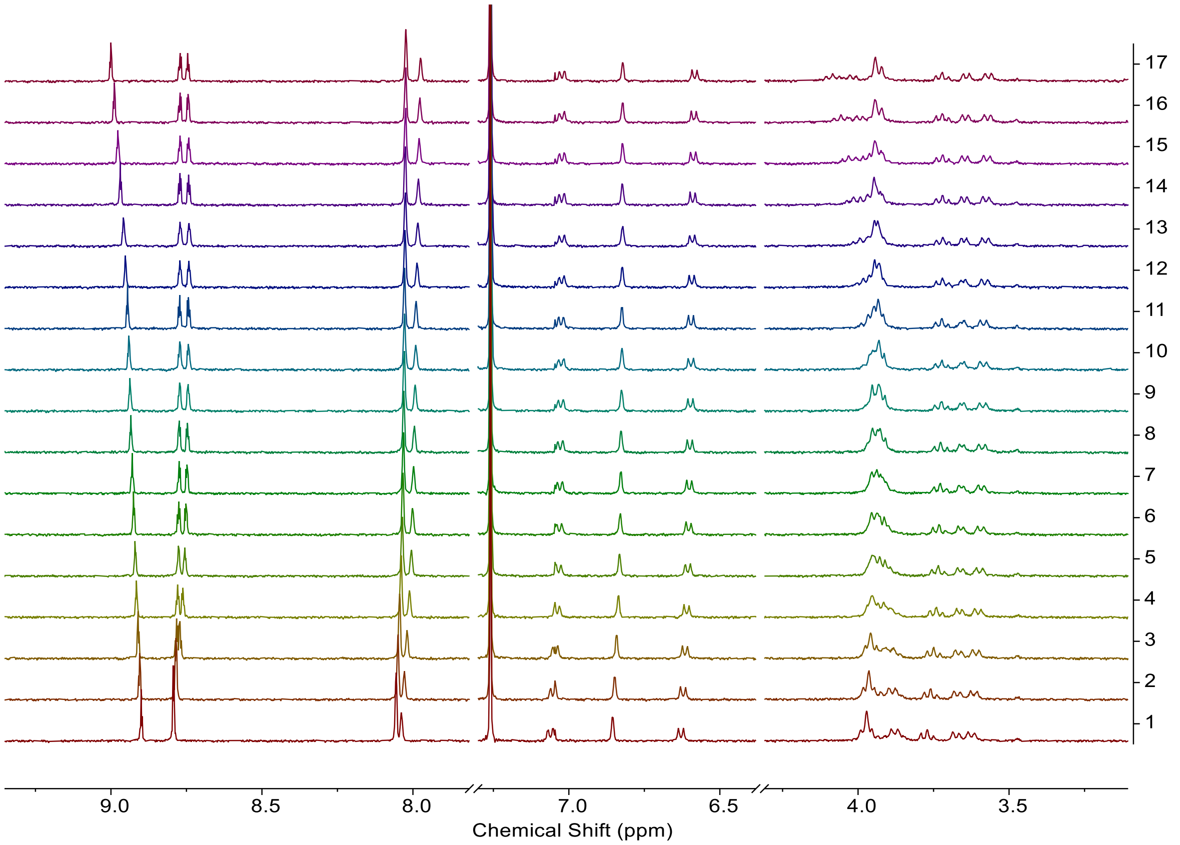

Figure S34. Truncated ^{1}H NMR titration spectra of **1** (0.5 mM) in presence of 1 equiv. KBArF (0.5 mM) with TBABr in $CDCl_3/CD_3CN$ (3:1 v/v), 500 MHz, 298 K. Spectrum no. 1 – 0 equivalents of the guest (TBABr); spectrum no. 17 – 10 equivalents of the guest (TBABr).

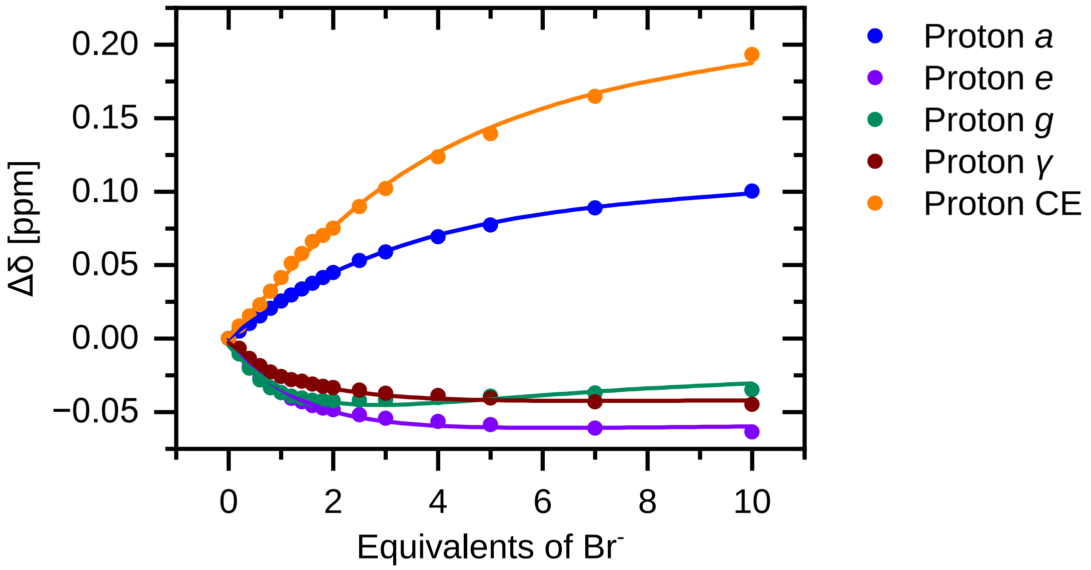

Figure S35. Examples of anion binding isotherms obtained during ^{1}H NMR titration of **1** (0.5 mM) in presence of 1 equiv. KBArF (0.5 mM) with TBABr in $CDCl_3/CD_3CN$ (3:1 v/v). Circles represent experimental data and the lines represent the fitted isotherms obtained using Bindfit (Model 1:2).

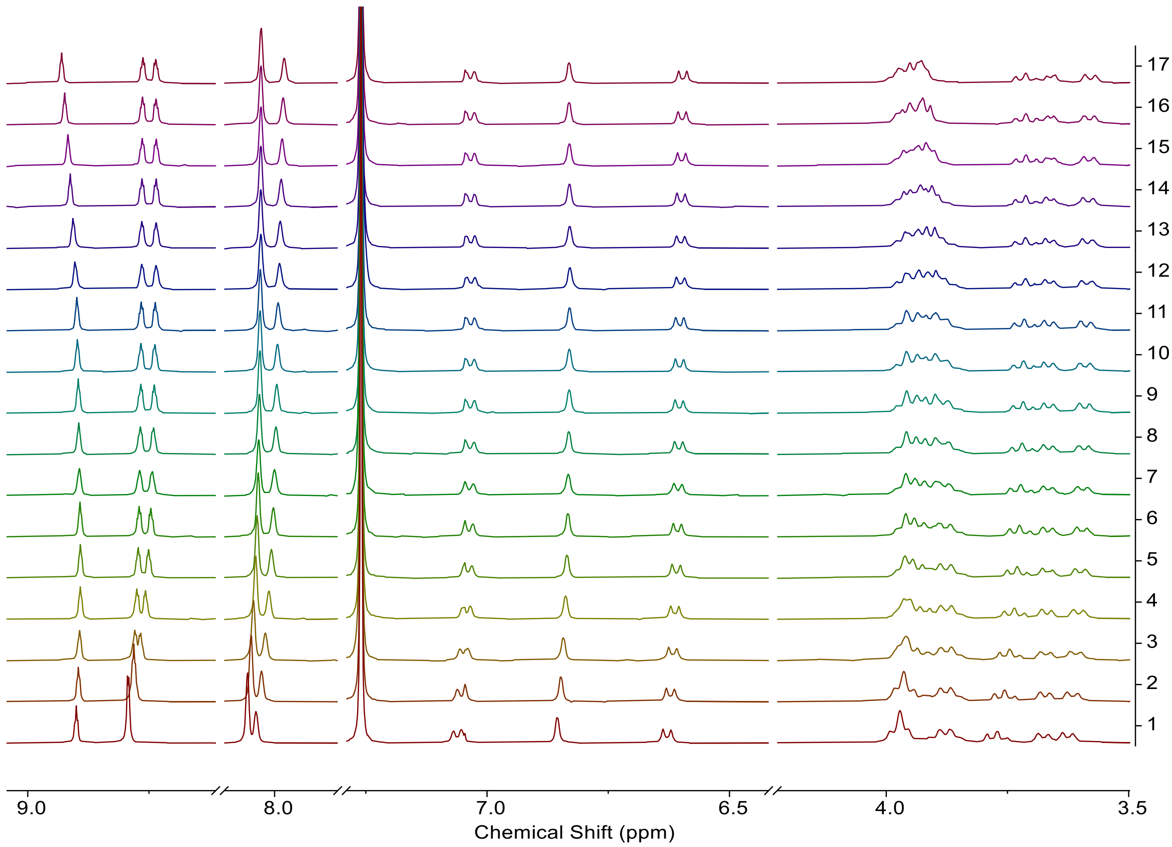

Figure S36. Truncated ^{1}H NMR titration spectra of **1** (0.5 mM) in presence of 1 equiv. KBArF (0.5 mM) with TBAI in $CDCl_3/CD_3CN$ (3:1 v/v), 500 MHz, 298 K. Spectrum no. 1 – 0 equivalents of the guest (TBAI); spectrum no. 17 – 10 equivalents of the guest (TBAI).

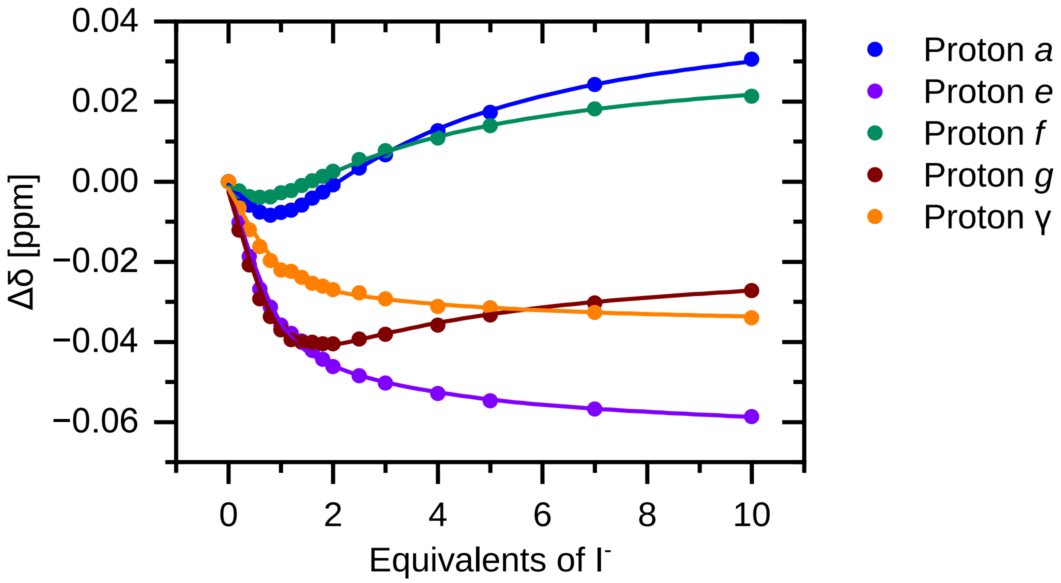

Figure S37. Examples of anion binding isotherms obtained during ^{1}H NMR titration of **1** (0.5 mM) in presence of 1 equiv. KBArF (0.5 mM) with TBAI in $CDCl_3/CD_3CN$ (3:1 v/v). Circles represent experimental data and the lines represent the fitted isotherms obtained using Bindfit (Model 1:2).

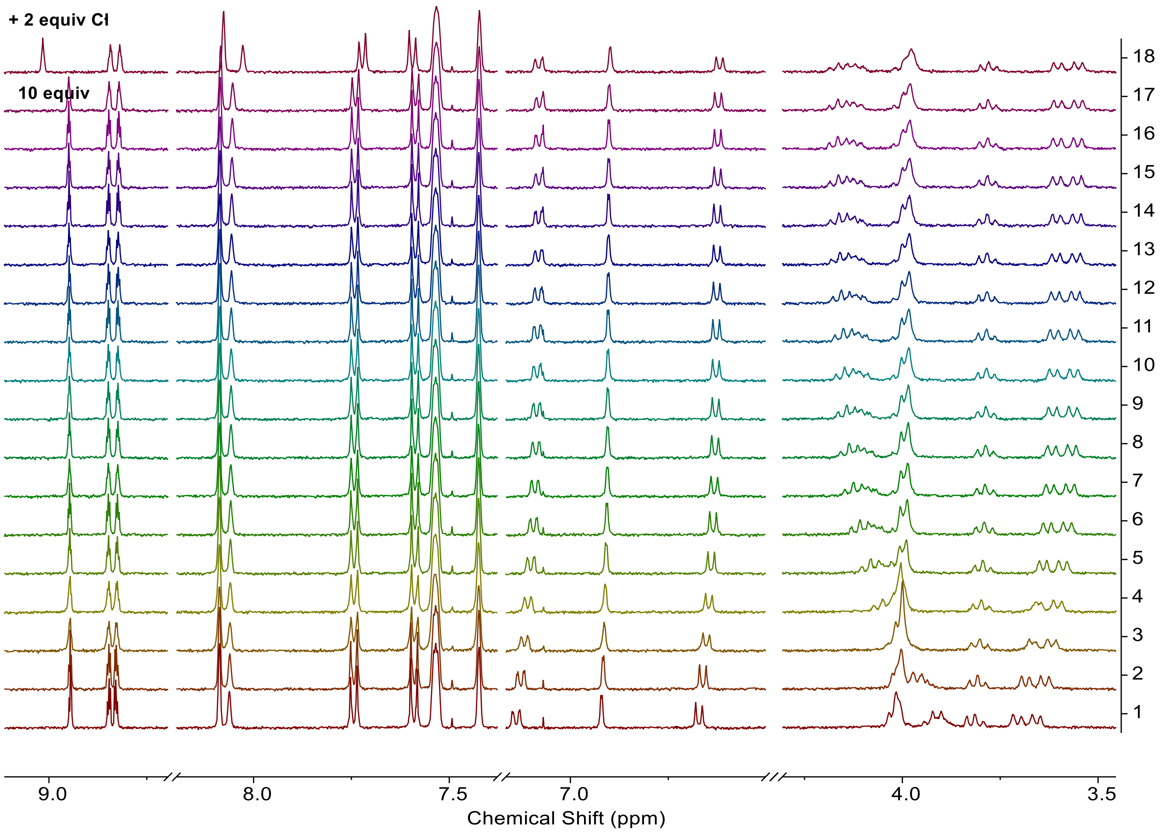

Figure S38. Truncated ^{1}H NMR titration spectra of **1** (0.5 mM) in presence of 1 equiv. KBArF (0.5 mM) with $TBANO_3$ in $CDCl_3/CD_3CN$ (3:1 v/v), 500 MHz, 298 K. Spectrum no. 1 – 0 equivalents of the guest ($TBANO_3$); spectrum no. 17 – 10 equivalents of the guest ($TBANO_3$); spectrum no.18 – 10 equivalents of $TBANO_3$ + 2 equivalents of TBACl.

S2.3 Titrations of Receptor 2

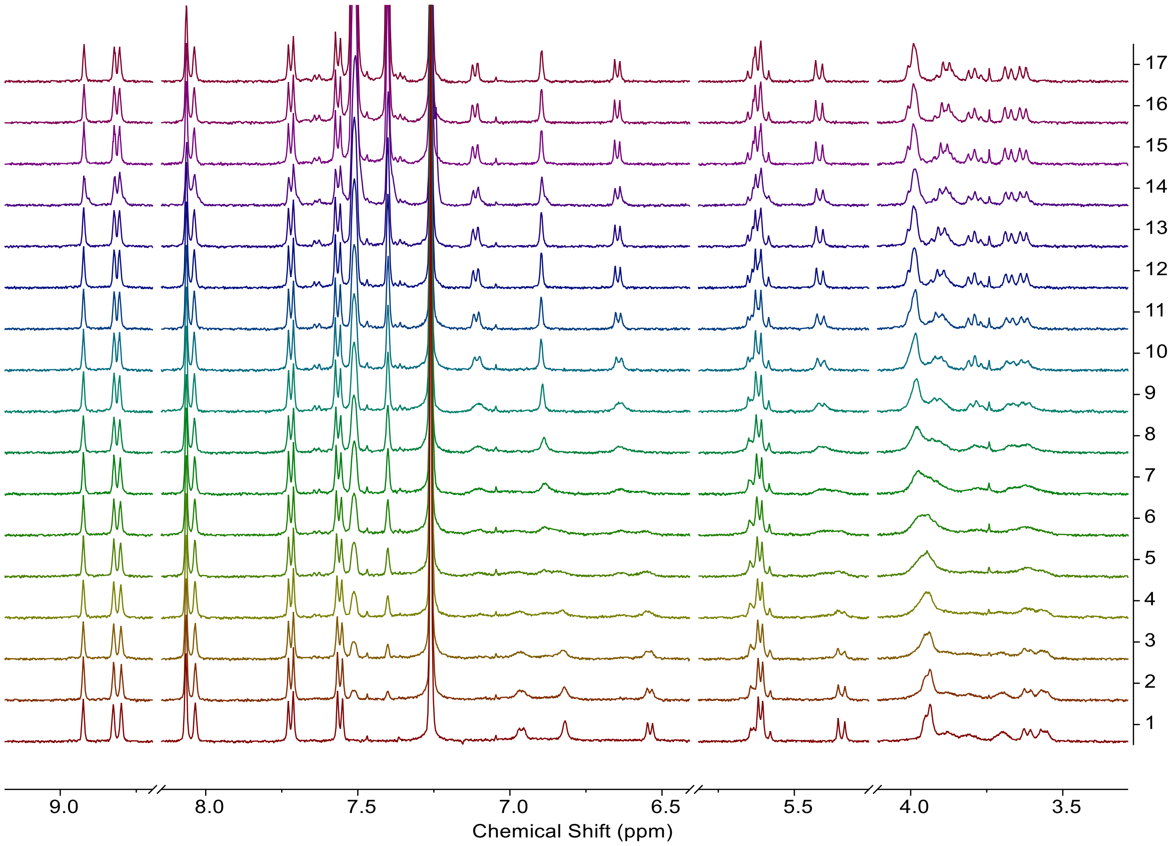

Figure S39. Truncated ^{1}H NMR titration spectra of **2** (0.5 mM) with KBArF in $CDCl_3/CD_3CN$ (3:1 v/v), 500 MHz, 298 K. Spectrum no. 1 – 0 equivalents of the guest (KBArF); spectrum no. 17 – 10 equivalents of the guest (KBArF).

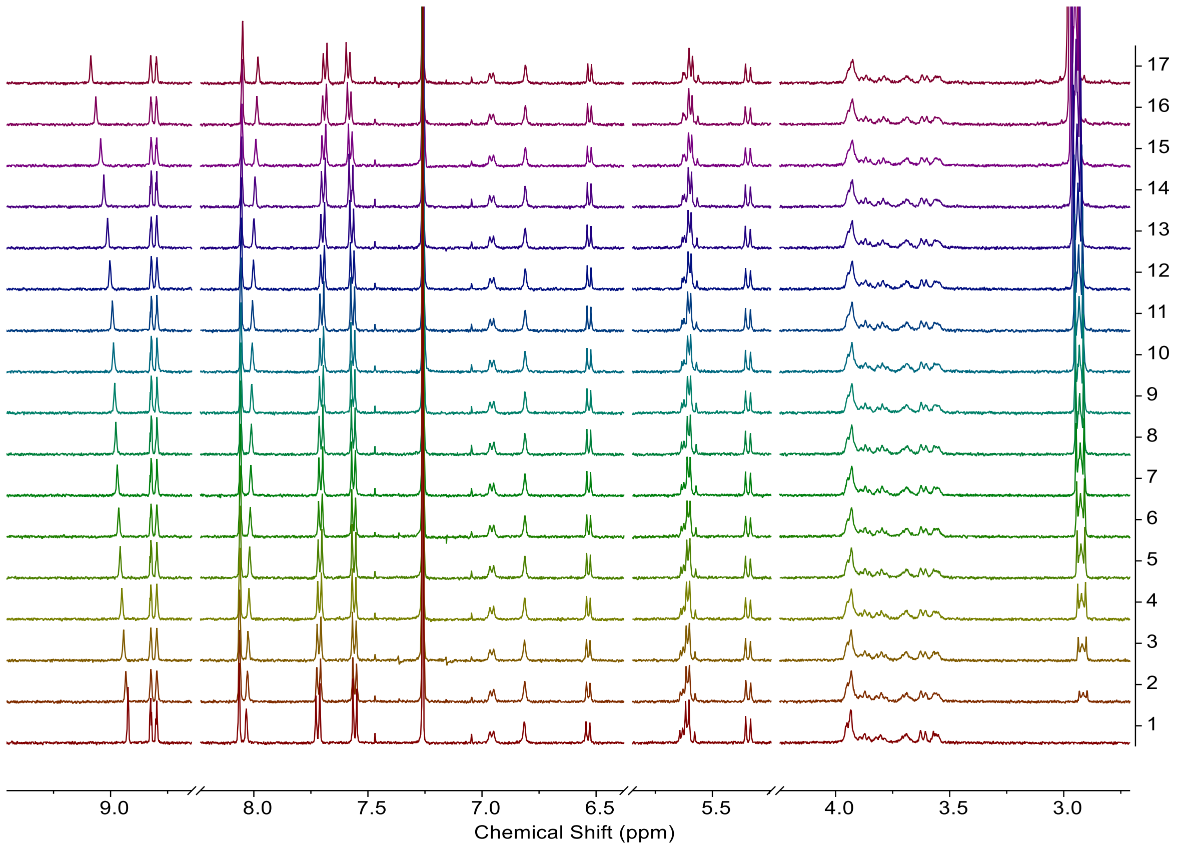

Figure S40. Truncated ^{1}H NMR titration spectra of **2** (0.5 mM) with TBACl in $CDCl_3/CD_3CN$ (3:1 v/v), 500 MHz, 298 K. Spectrum no. 1 – 0 equivalents of the guest (TBACl); spectrum no. 17 – 10 equivalents of the guest (TBACl).

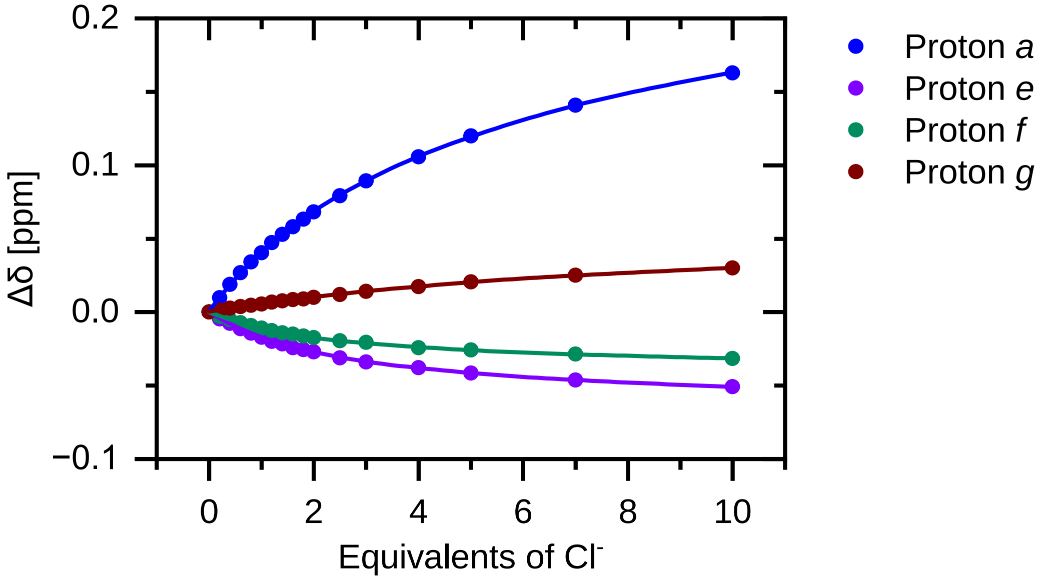

Figure S41. Examples of anion binding isotherms obtained during ^{1}H NMR titration of **2** (0.5 mM) with TBACl in $CDCl_3/CD_3CN$ (3:1 v/v). Circles represent experimental data and the lines represent the fitted isotherms obtained using Bindfit (Model 1:2).

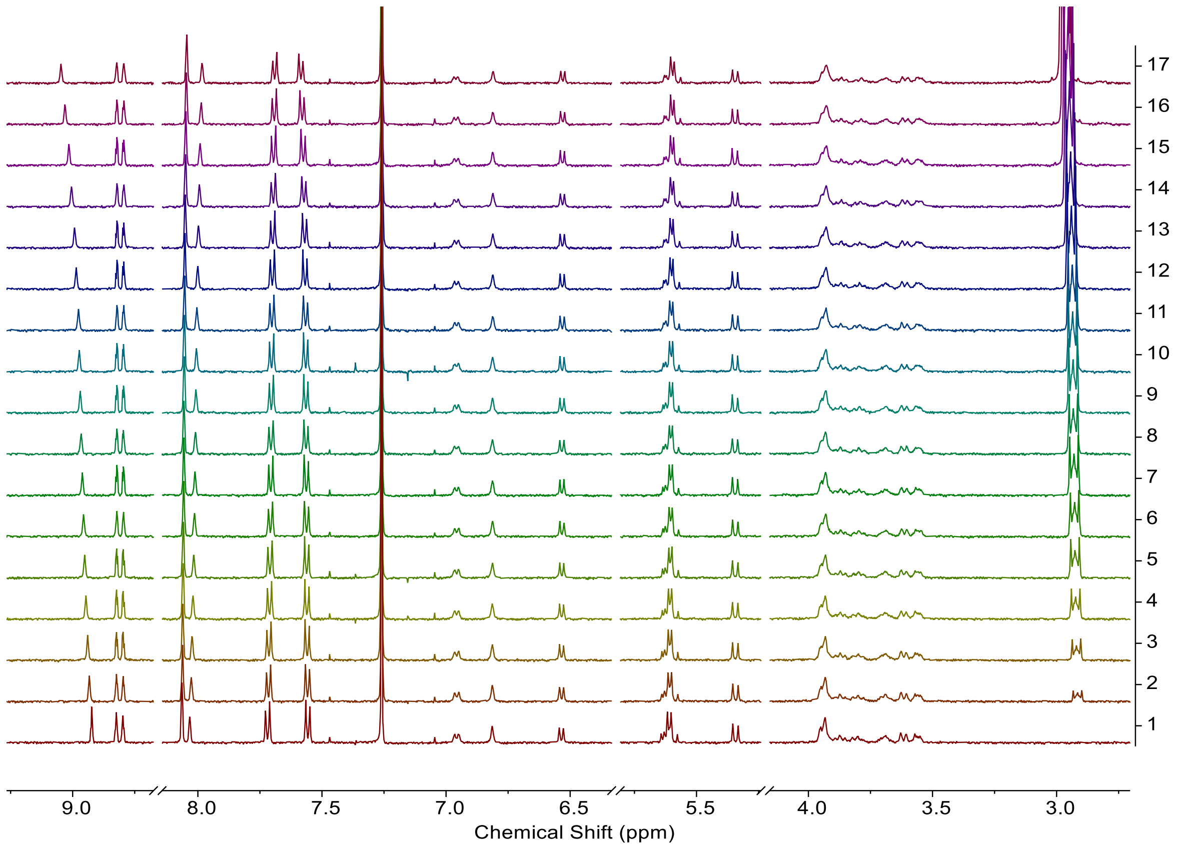

Figure S42. Truncated ^{1}H NMR titration spectra of **2** (0.5 mM) with TBABr in $CDCl_3/CD_3CN$ (3:1 v/v), 500 MHz, 298 K. Spectrum no. 1 – 0 equivalents of the guest (TBABr); spectrum no. 17 – 10 equivalents of the guest (TBABr).

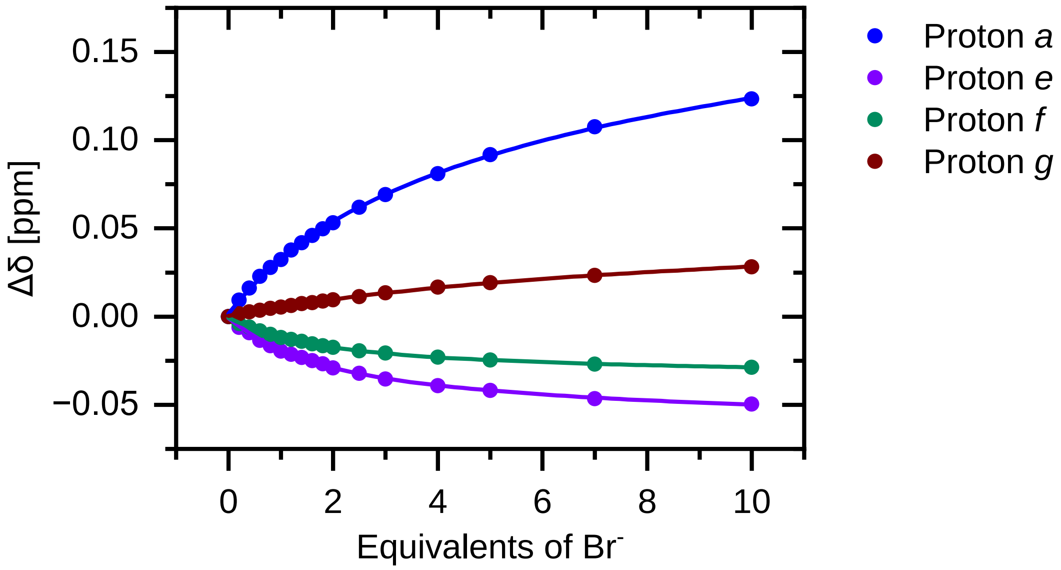

Figure S43. Examples of anion binding isotherms obtained during ^{1}H NMR titration of **2** (0.5 mM) with TBABr in $CDCl_3/CD_3CN$ (3:1 v/v). Circles represent experimental data and the lines represent the fitted isotherms obtained using Bindfit (Model 1:2).

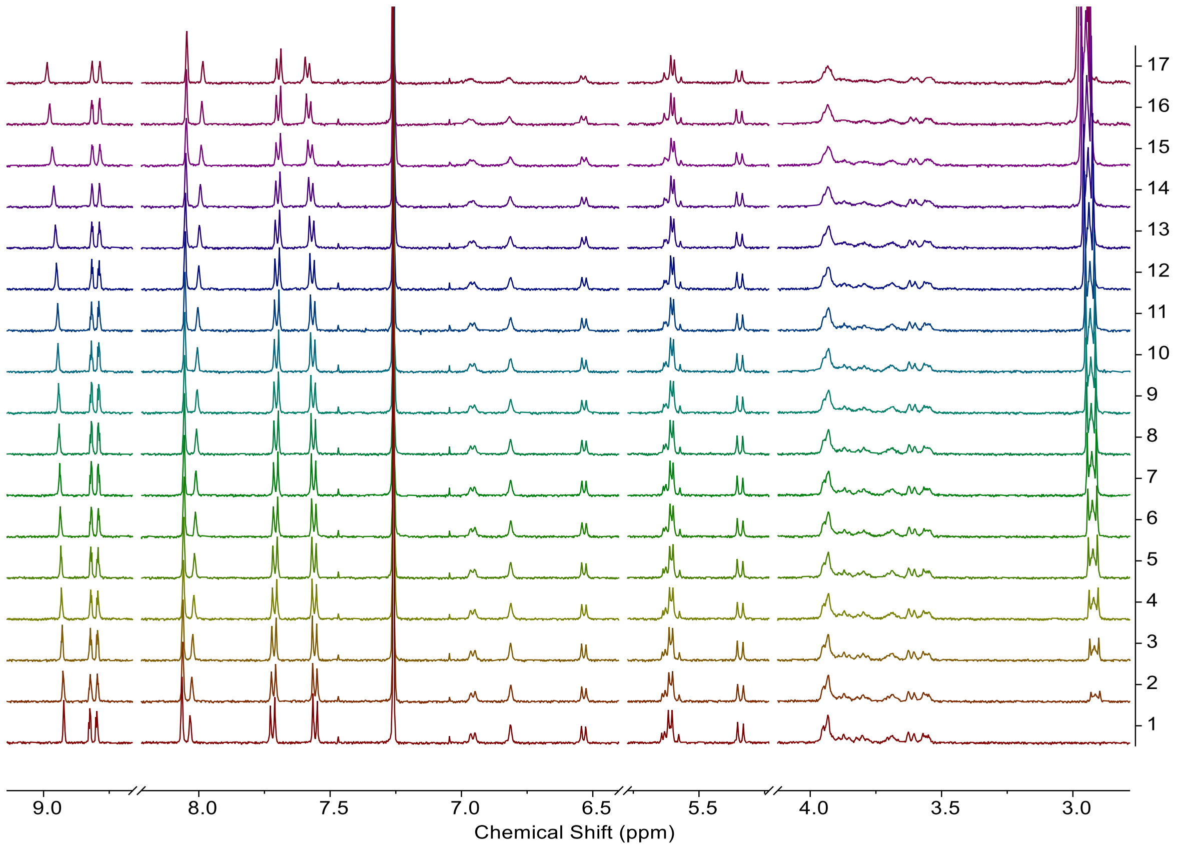

Figure S44. Truncated ^{1}H NMR titration spectra of **2** (0.5 mM) with TBAI in $CDCl_3/CD_3CN$ (3:1 v/v), 500 MHz, 298 K. Spectrum no. 1 – 0 equivalents of the guest (TBAI); spectrum no. 17 – 10 equivalents of the guest (TBAI).

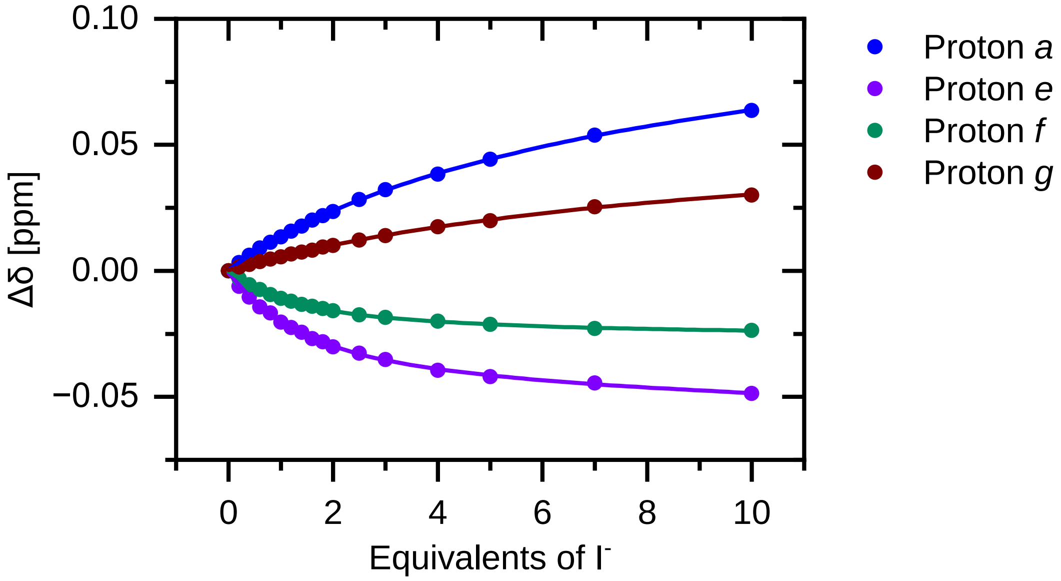

Figure S45. Examples of anion binding isotherms obtained during ^{1}H NMR titration of **2** (0.5 mM) with TBAI in $CDCl_3/CD_3CN$ (3:1 v/v). Circles represent experimental data and the lines represent the fitted isotherms obtained using Bindfit (Model 1:2).

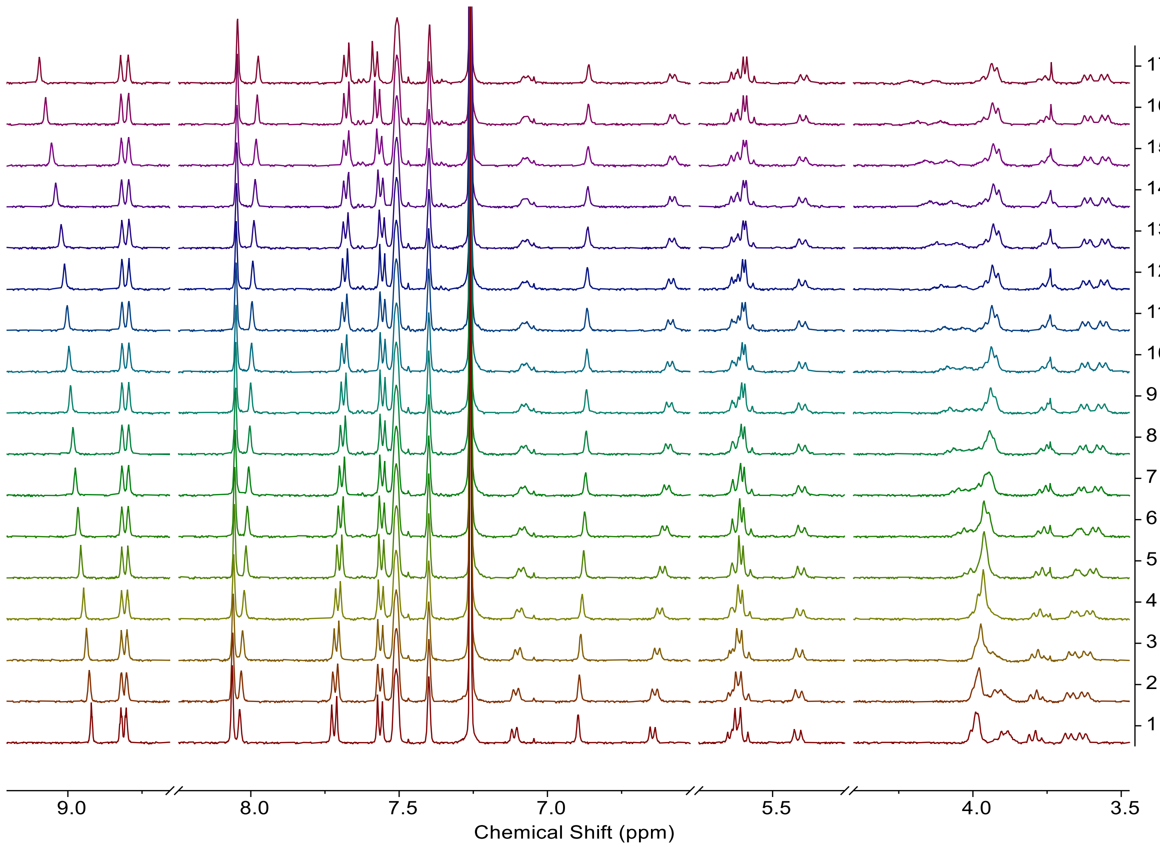

Figure S46. Truncated ^{1}H NMR titration spectra of **2** (0.5 mM) in presence of 1 equiv. KBArF (0.5 mM) with TBACl in $CDCl_3/CD_3CN$ (3:1 v/v), 500 MHz, 298 K. Spectrum no. 1 – 0 equivalents of the guest (TBACl); spectrum no. 17 – 10 equivalents of the guest (TBACl).

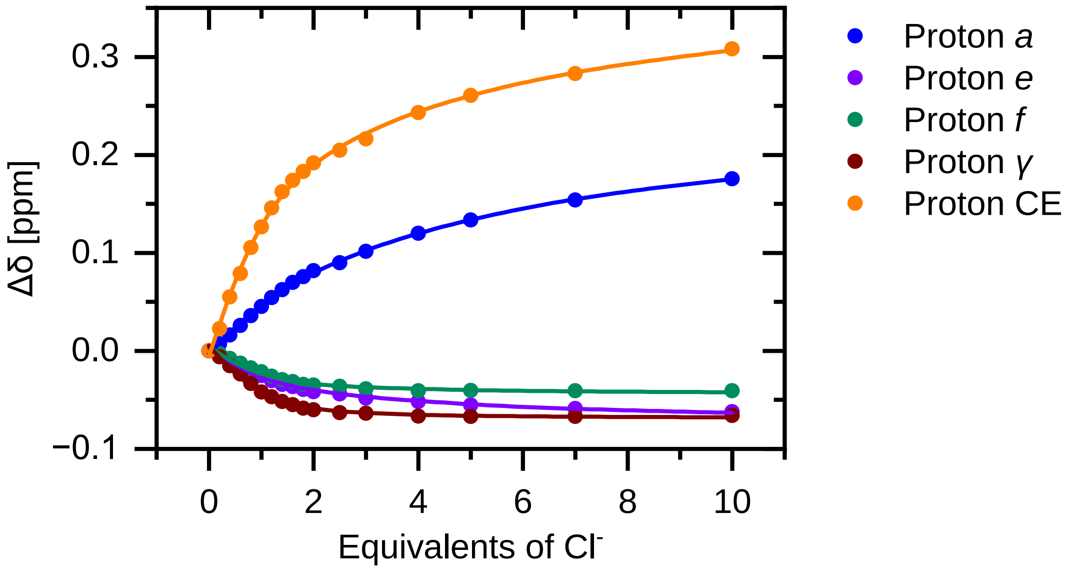

Figure S47. Examples of anion binding isotherms obtained during ^{1}H NMR titration of **2** (0.5 mM) in presence of 1 equiv. KBArF (0.5 mM) with TBACl in $CDCl_3/CD_3CN$ (3:1 v/v). Circles represent experimental data and the lines represent the fitted isotherms obtained using Bindfit (Model 1:2).

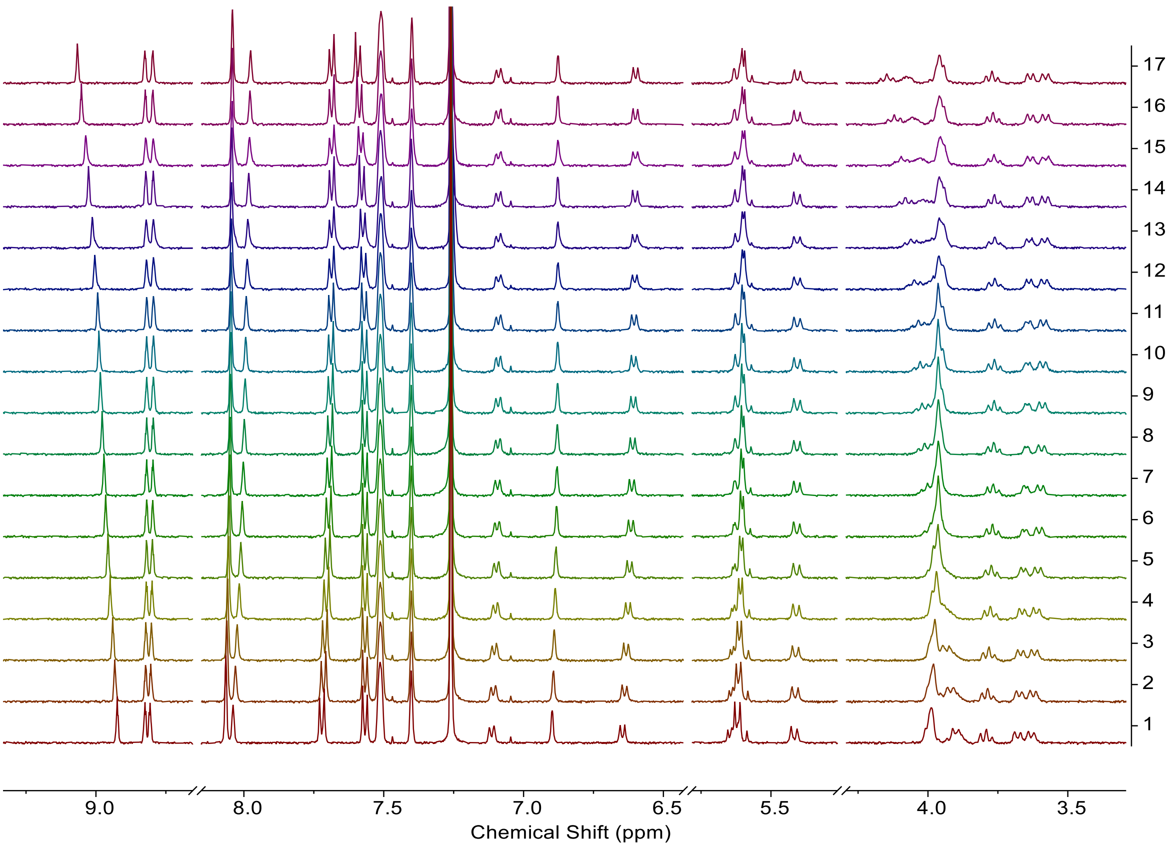

Figure S48. Truncated ^{1}H NMR titration spectra of **2** (0.5 mM) in presence of 1 equiv. KBArF (0.5 mM) with TBABr in $CDCl_3/CD_3CN$ (3:1 v/v), 500 MHz, 298 K. Spectrum no. 1 – 0 equivalents of the guest (TBABr); spectrum no. 17 – 10 equivalents of the guest (TBABr).

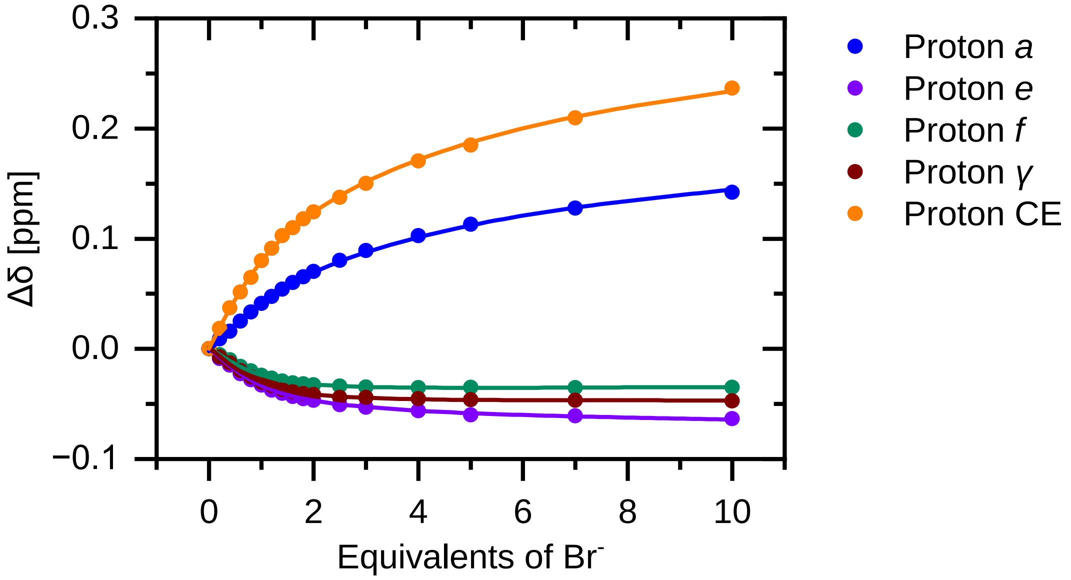

Figure S49. Examples of anion binding isotherms obtained during ^{1}H NMR titration of **2** (0.5 mM) in presence of 1 equiv. KBArF (0.5 mM) with TBABr in $CDCl_3/CD_3CN$ (3:1 v/v). Circles represent experimental data and the lines represent the fitted isotherms obtained using Bindfit (Model 1:2).

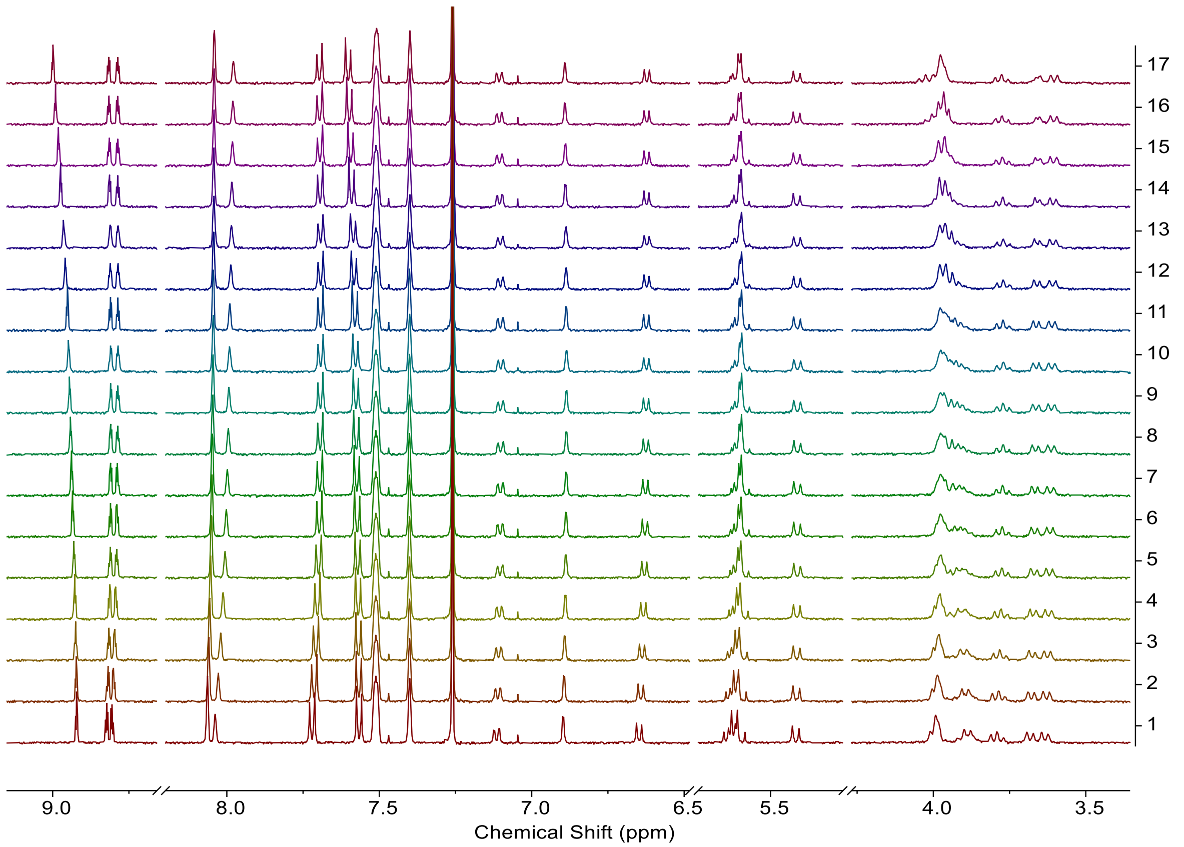

Figure S50. Truncated ^{1}H NMR titration spectra of **2** (0.5 mM) in presence of 1 equiv. KBArF (0.5 mM) with TBAI in $CDCl_3/CD_3CN$ (3:1 v/v), 500 MHz, 298 K. Spectrum no. 1 – 0 equivalents of the guest (TBAI); spectrum no. 17 – 10 equivalents of the guest (TBAI).

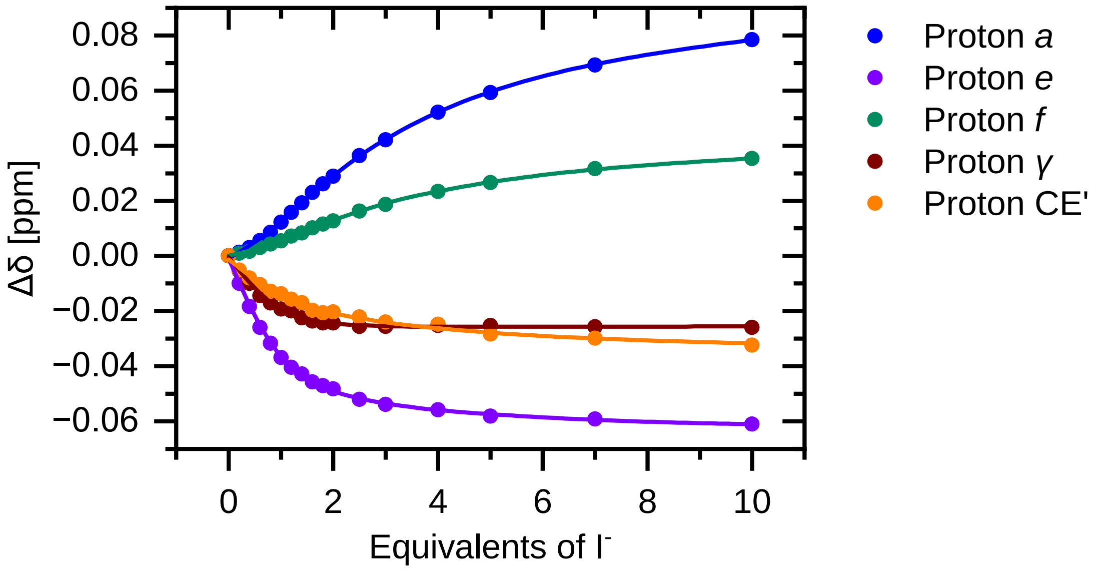

Figure S51. Examples of anion binding isotherms obtained during ^{1}H NMR titration of **2** (0.5 mM) in presence of 1 equiv. KBArF (0.5 mM) with TBAI in $CDCl_3/CD_3CN$ (3:1 v/v). Circles represent experimental data and the lines represent the fitted isotherms obtained using Bindfit (Model 1:2).

S2.4 Titrations of Receptor 9

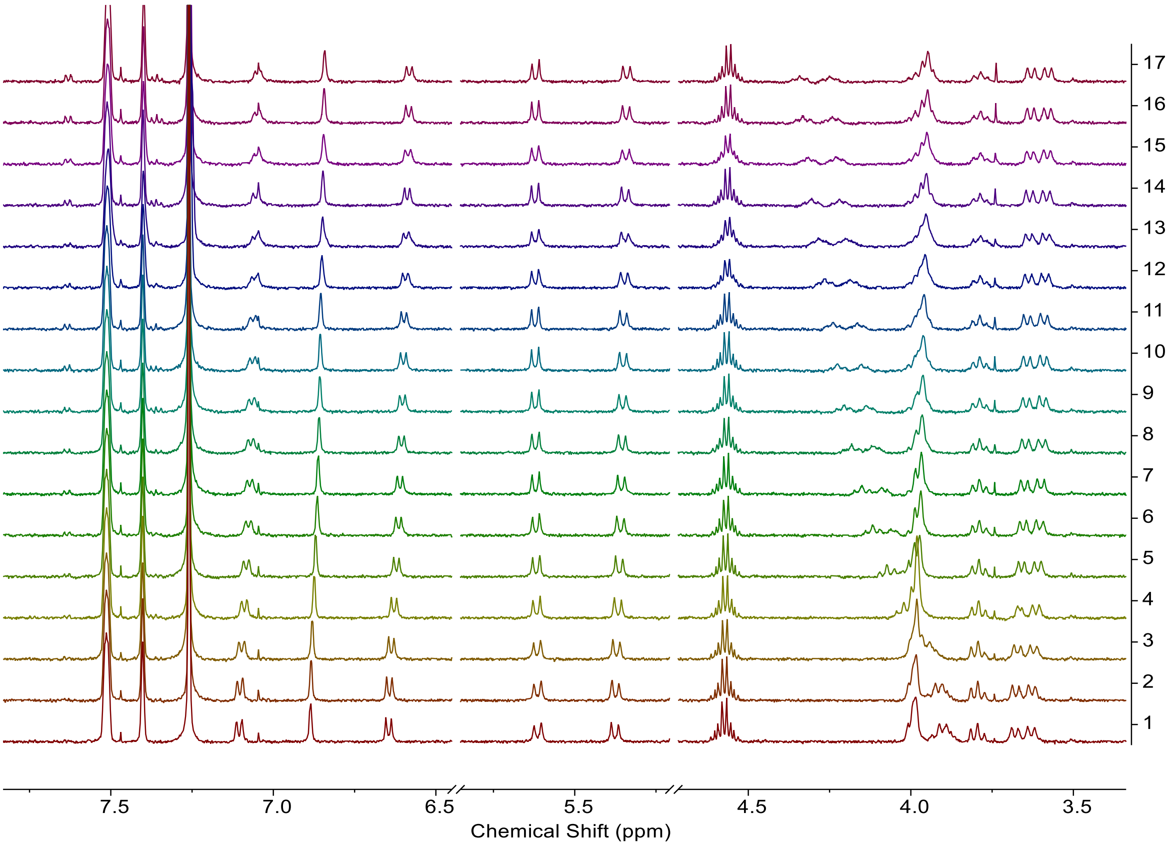

Figure S52. Truncated ^{1}H NMR titration spectra of **9** (0.5 mM) in presence of 1 equiv. KBArF (0.5 mM) with TBACl in $CDCl_3/CD_3CN$ (3:1 v/v), 500 MHz, 298 K. Spectrum no. 1 – 0 equivalents of the guest (TBACl); spectrum no. 17 – 10 equivalents of the guest (TBACl).

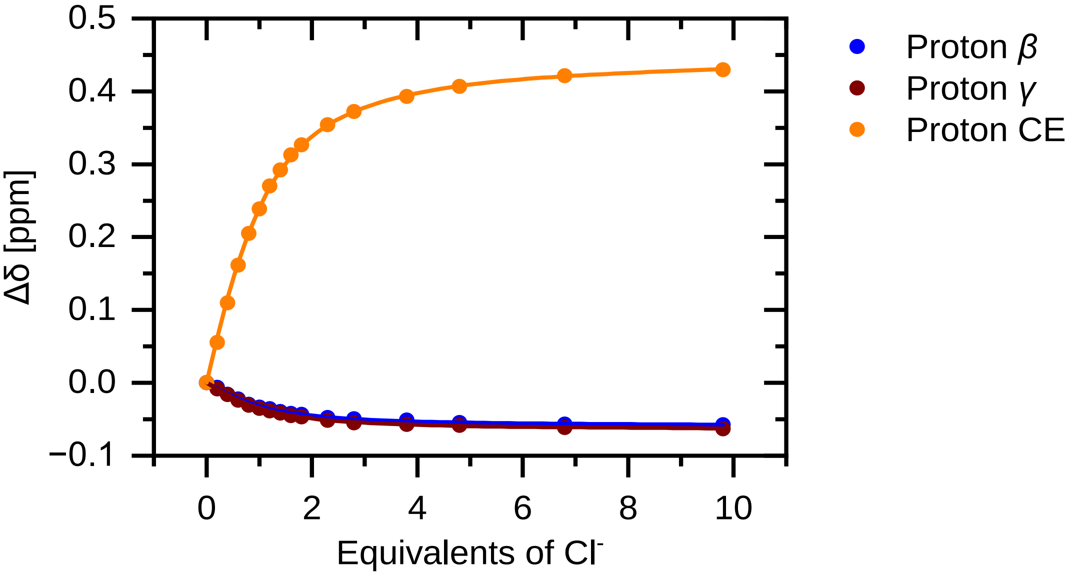

Figure S53. Examples of anion binding isotherms obtained during ^{1}H NMR titration of **9** (0.5 mM) in presence of 1 equiv. KBArF (0.5 mM) with TBACl in $CDCl_3/CD_3CN$ (3:1 v/v). Circles represent experimental data and the lines represent the fitted isotherms obtained using Bindfit (Model 1:1).

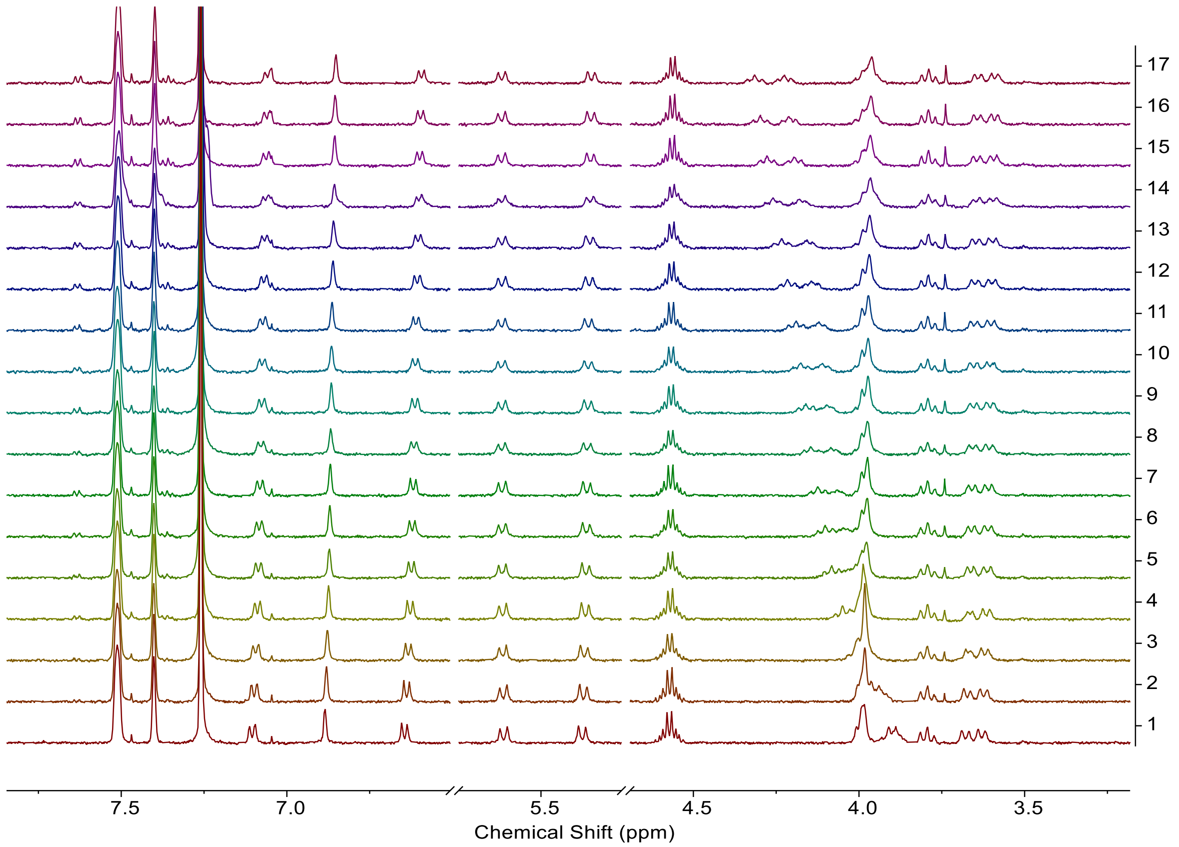

Figure S54. Truncated ^{1}H NMR titration spectra of **9** (0.5 mM) in presence of 1 equiv. KBArF (0.5 mM) with TBABr in $CDCl_3/CD_3CN$ (3:1 v/v), 500 MHz, 298 K. Spectrum no. 1 – 0 equivalents of the guest (TBABr); spectrum no. 17 – 10 equivalents of the guest (TBABr).

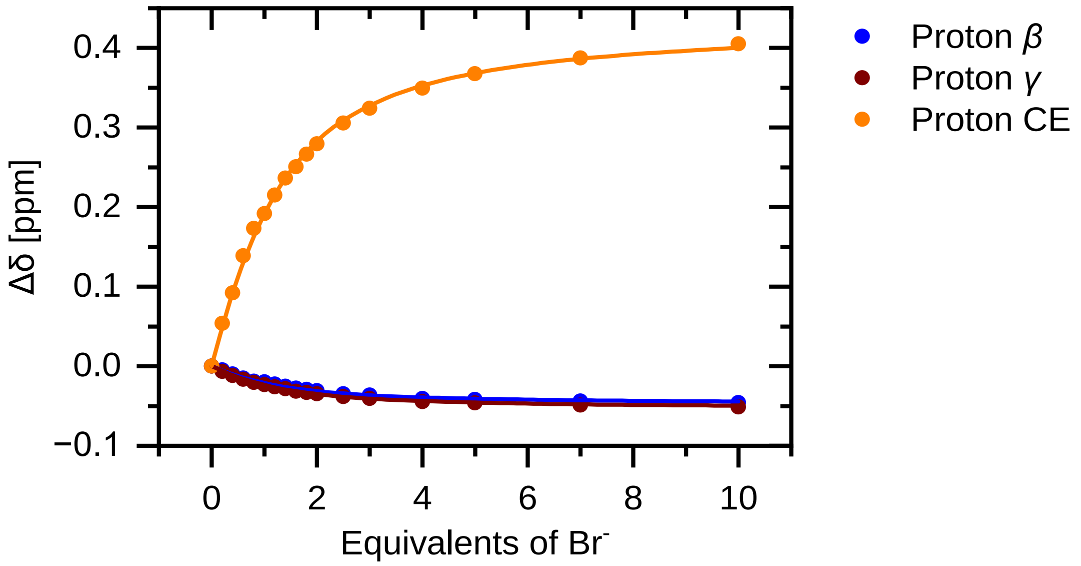

Figure S55. Examples of anion binding isotherms obtained during ^{1}H NMR titration of **9** (0.5 mM) in presence of 1 equiv. KBArF (0.5 mM) with TBABr in $CDCl_3/CD_3CN$ (3:1 v/v). Circles represent experimental data and the lines represent the fitted isotherms obtained using Bindfit (Model 1:1).

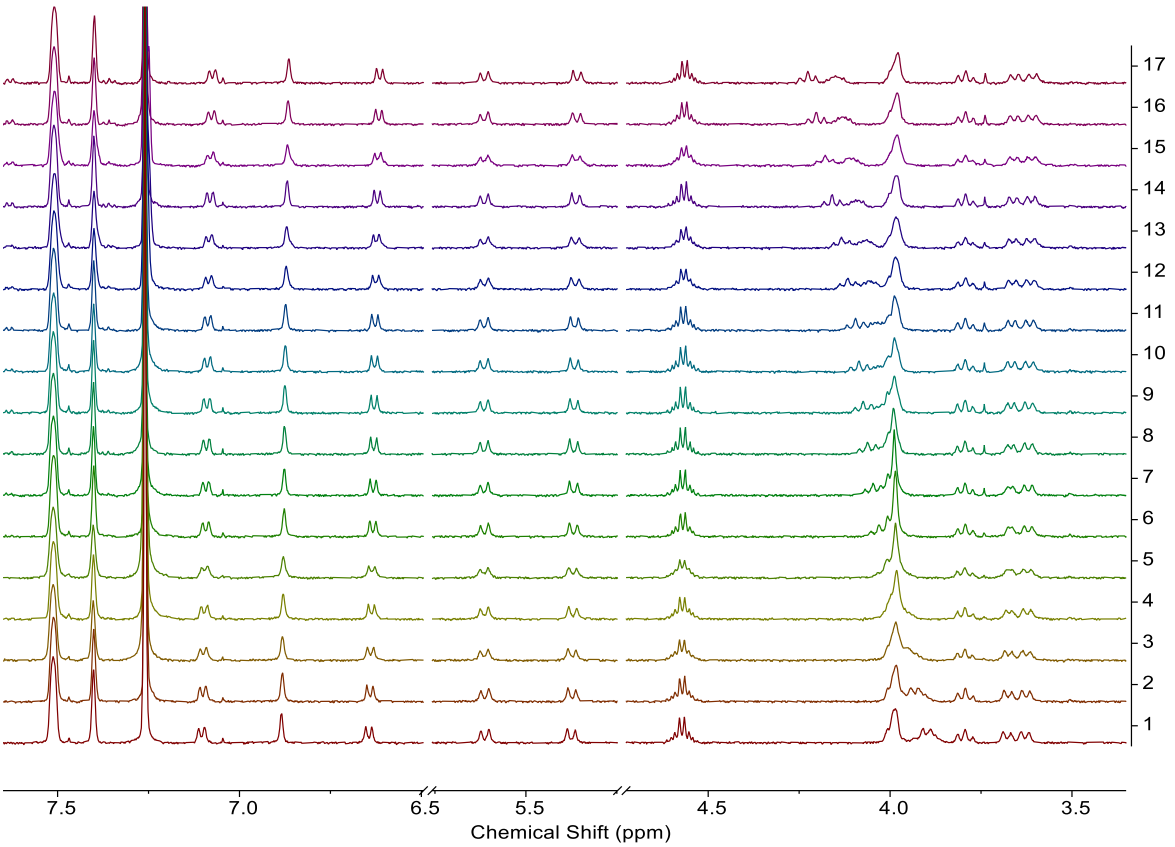

Figure S56. Truncated ^{1}H NMR titration spectra of **9** (0.5 mM) in presence of 1 equiv. KBArF (0.5 mM) with TBAI in $CDCl_3/CD_3CN$ (3:1 v/v), 500 MHz, 298 K. Spectrum no. 1 – 0 equivalents of the guest (TBAI); spectrum no. 17 – 10 equivalents of the guest (TBAI).

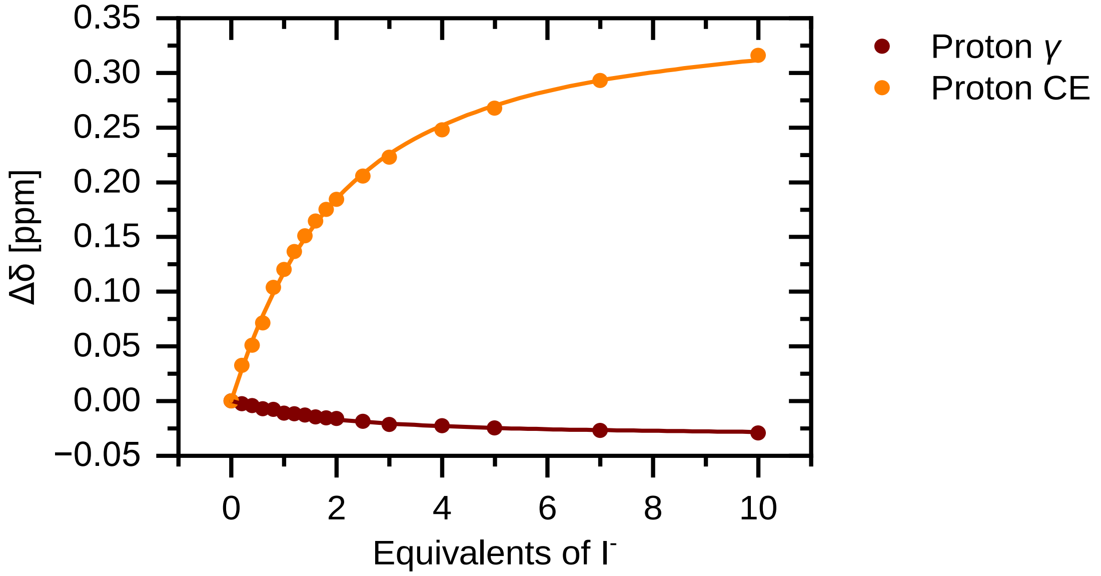

Figure S57. Examples of anion binding isotherms obtained during ^{1}H NMR titration of **9** (0.5 mM) in presence of 1 equiv. KBArF (0.5 mM) with TBAI in $CDCl_3/CD_3CN$ (3:1 v/v). Circles represent experimental data and the lines represent the fitted isotherms obtained using Bindfit (Model 1:1).

S2.5 Titrations of receptor 16

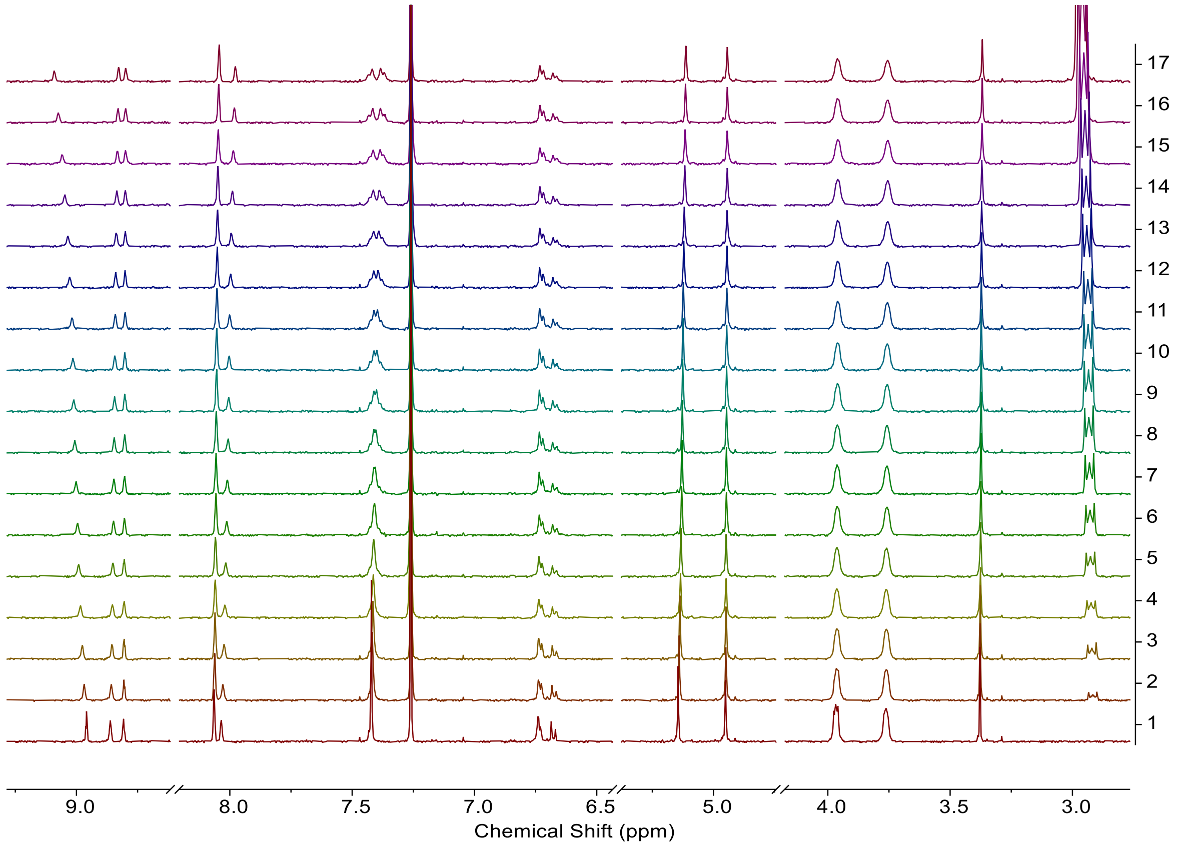

Figure S58. Truncated ^{1}H NMR titration spectra of **16** (0.5 mM) with TBACl in $CDCl_3/CD_3CN$ (3:1 v/v), 500 MHz, 298 K. Spectrum no. 1 – 0 equivalents of the guest (TBACl); spectrum no. 17 – 10 equivalents of the guest (TBACl).

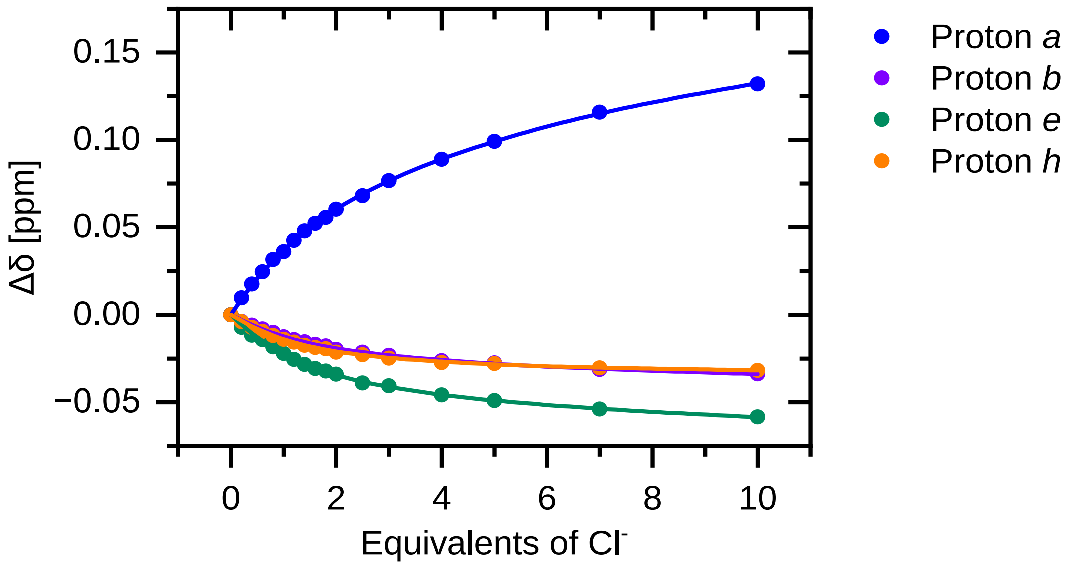

Figure S59. Examples of anion binding isotherms obtained during ^{1}H NMR titration of **16** (0.5 mM) with TBACl in $CDCl_3/CD_3CN$ (3:1 v/v). Circles represent experimental data and the lines represent the fitted isotherms obtained using Bindfit (Model 1:2).

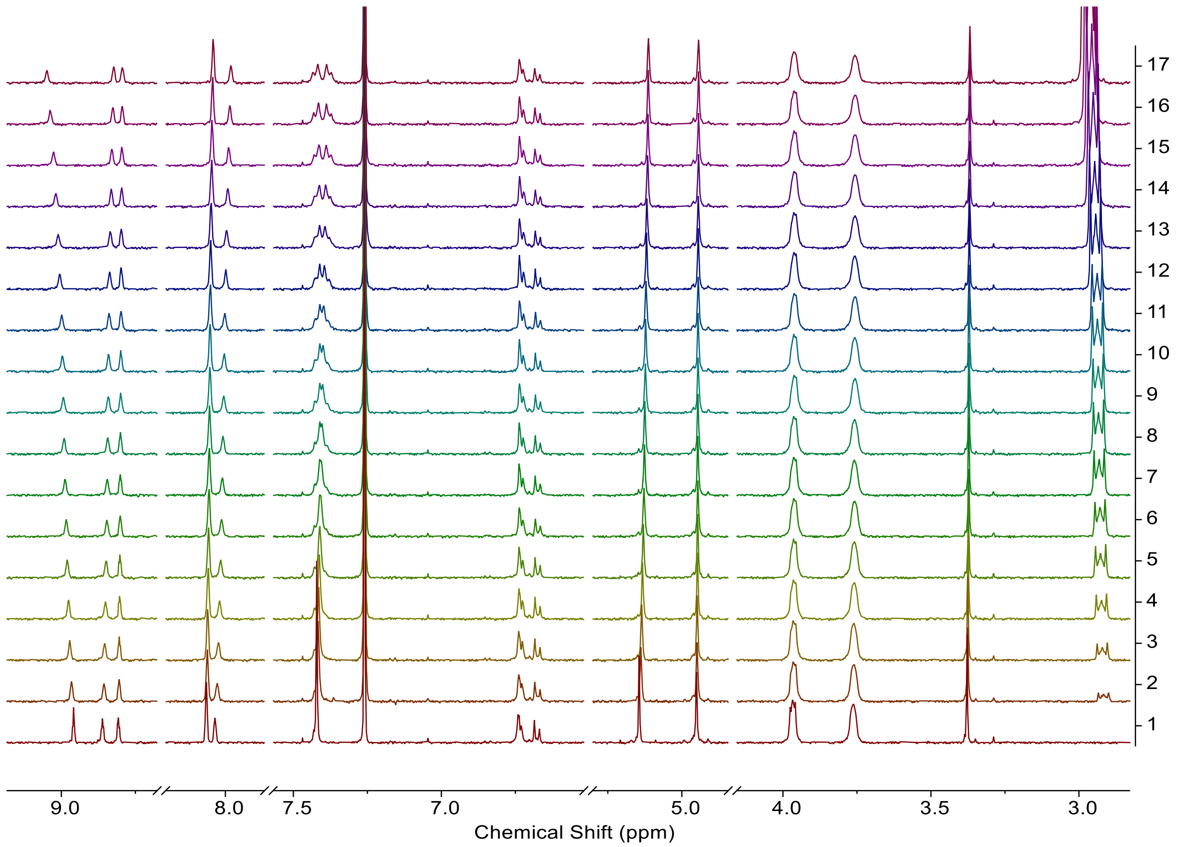

Figure S60. Truncated ^{1}H NMR titration spectra of **16** (0.5 mM) with TBABr in $CDCl_3/CD_3CN$ (3:1 v/v), 500 MHz, 298 K. Spectrum no. 1 – 0 equivalents of the guest (TBABr); spectrum no. 17 – 10 equivalents of the guest (TBABr).

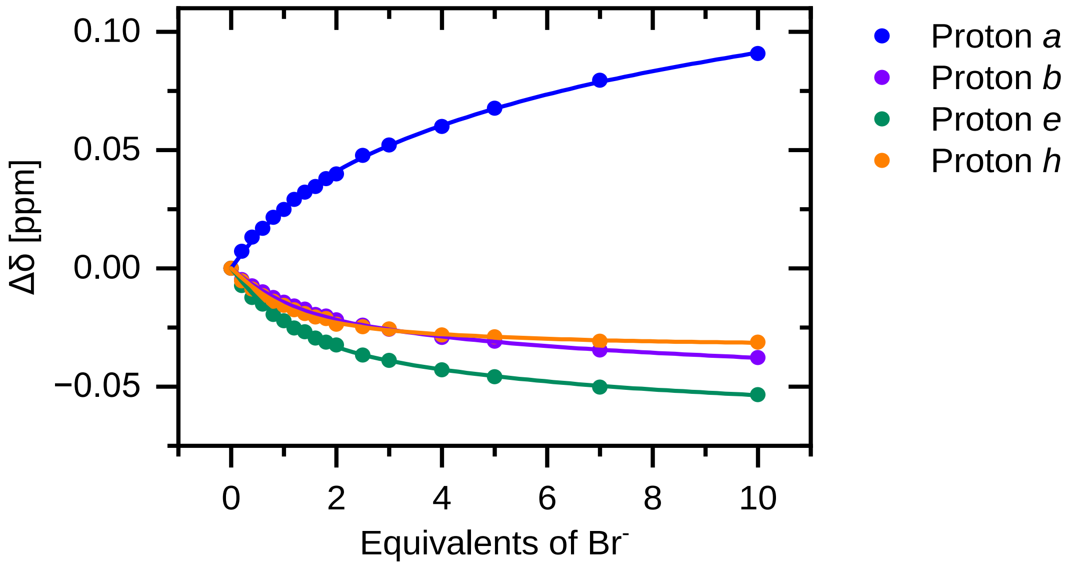

Figure S61. Examples of anion binding isotherms obtained during ^{1}H NMR titration of **16** (0.5 mM) with TBABr in $CDCl_3/CD_3CN$ (3:1 v/v). Circles represent experimental data and the lines represent the fitted isotherms obtained using Bindfit (Model 1:2).

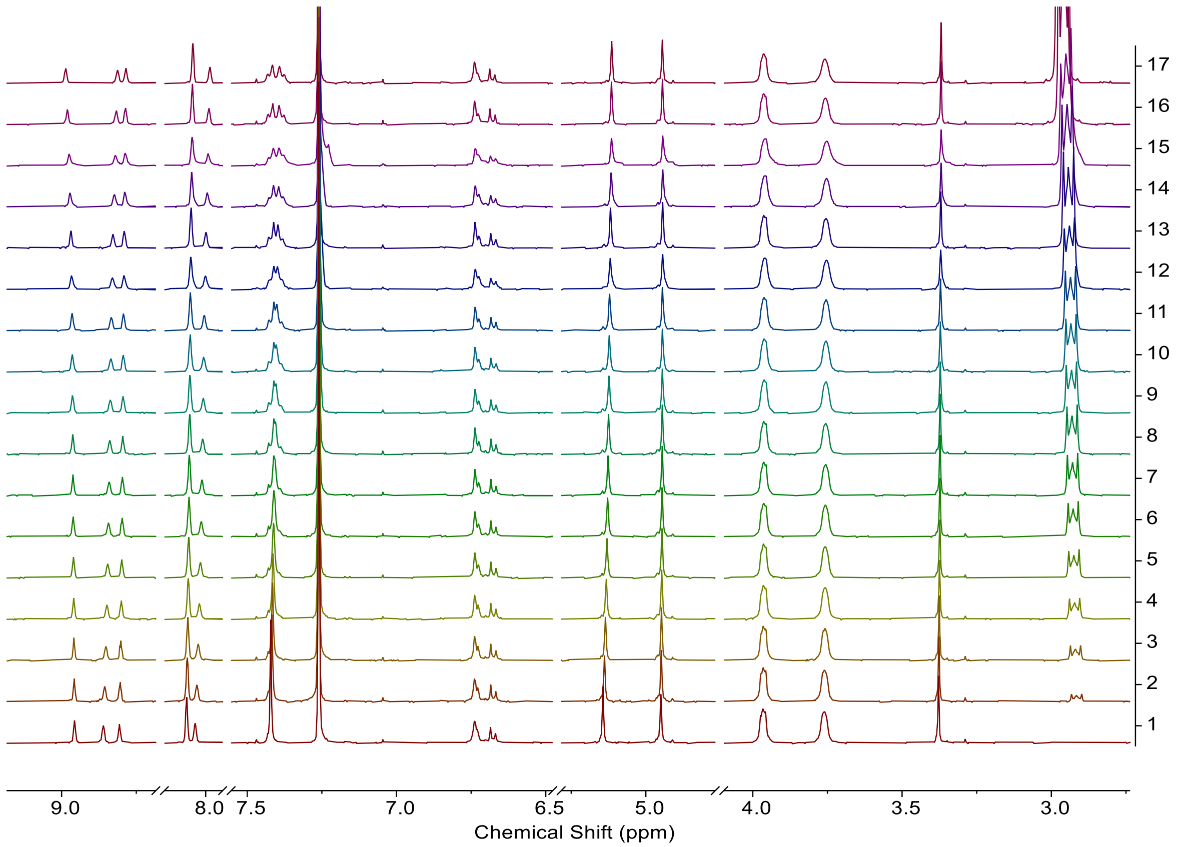

Figure S62. Truncated ^{1}H NMR titration spectra of **16** (0.5 mM) with TBAI in $CDCl_3/CD_3CN$ (3:1 v/v), 500 MHz, 298 K. Spectrum no. 1 – 0 equivalents of the guest (TBAI); spectrum no. 17 – 10 equivalents of the guest (TBAI).

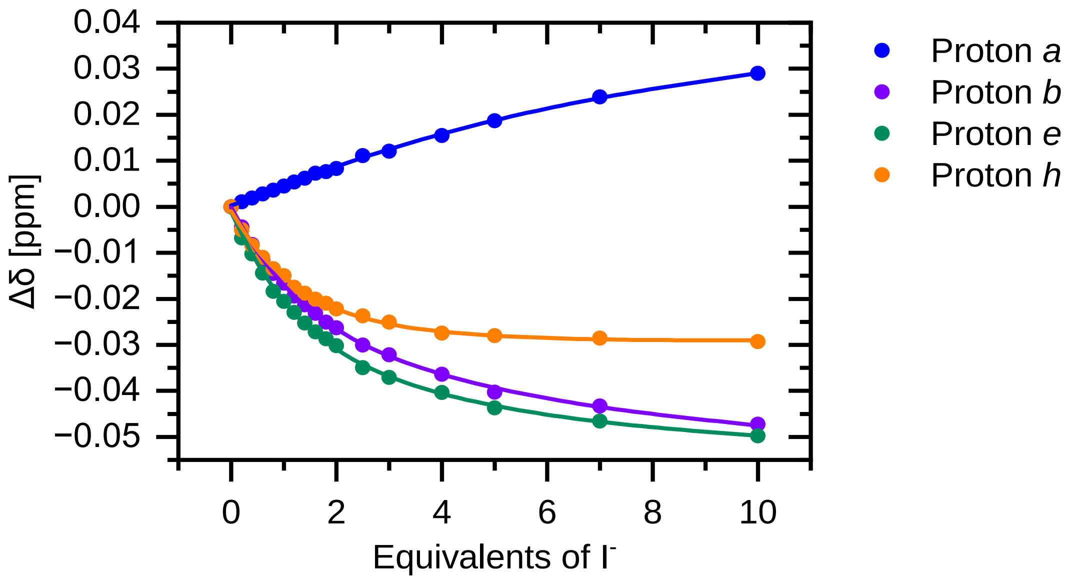

Figure S63. Examples of anion binding isotherms obtained during ^{1}H NMR titration of **16** (0.5 mM) with TBAI in $CDCl_3/CD_3CN$ (3:1 v/v). Circles represent experimental data and the lines represent the fitted isotherms obtained using Bindfit (Model 1:2).

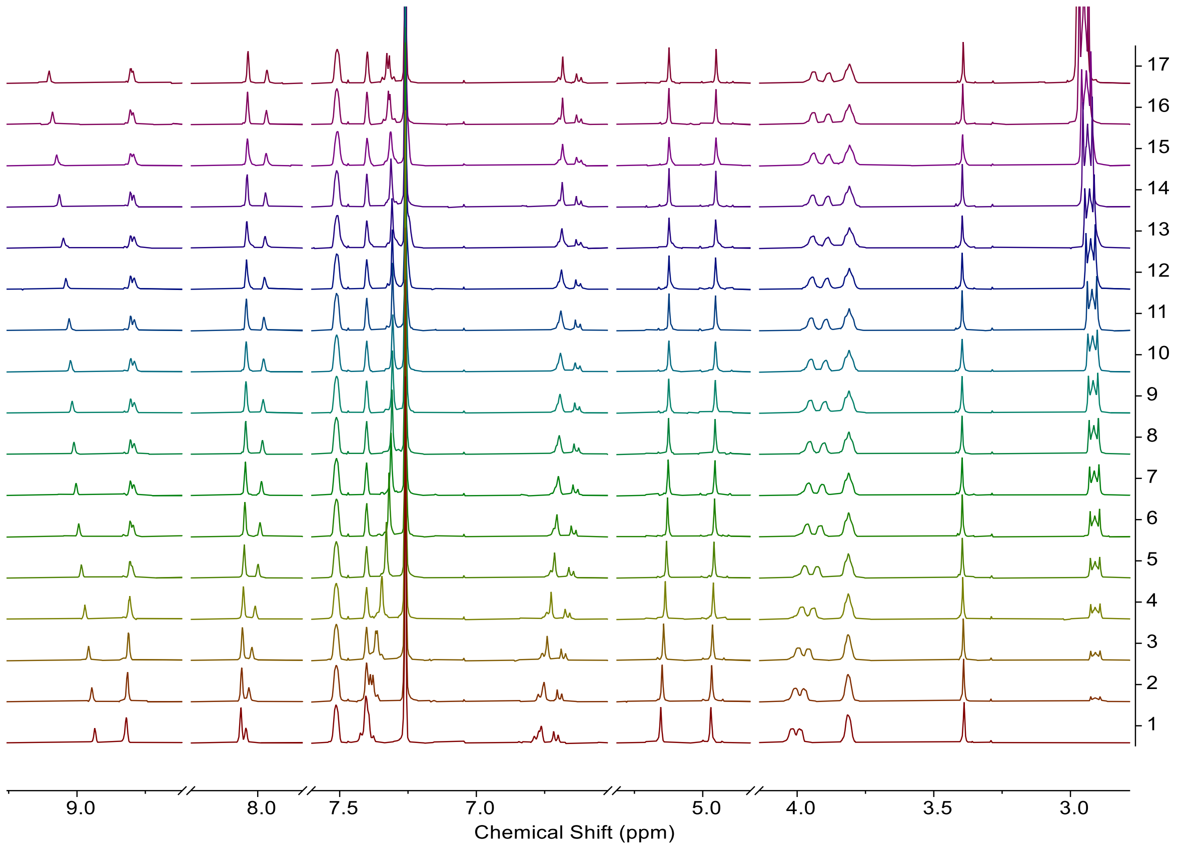

Figure S64. Truncated ^{1}H NMR titration spectra of **16** (0.5 mM) in presence of 1 equiv. KBArF (0.5 mM) with TBACl in $CDCl_3/CD_3CN$ (3:1 v/v), 500 MHz, 298 K. Spectrum no. 1 – 0 equivalents of the guest (TBACl); spectrum no. 17 – 10 equivalents of the guest (TBACl).

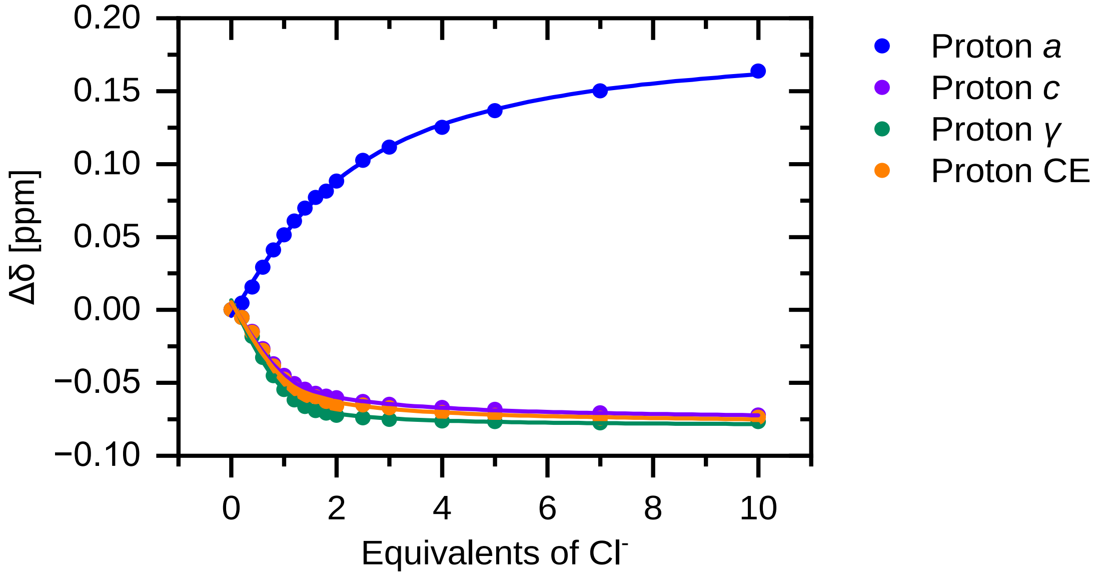

Figure S65. Examples of anion binding isotherms obtained during ^{1}H NMR titration of **1** (0.5 mM) in presence of 1 equiv. KBArF (0.5 mM) with TBACl in $CDCl_3/CD_3CN$ (3:1 v/v). Circles represent experimental data and the lines represent the fitted isotherms obtained using Bindfit (Model 1:2).

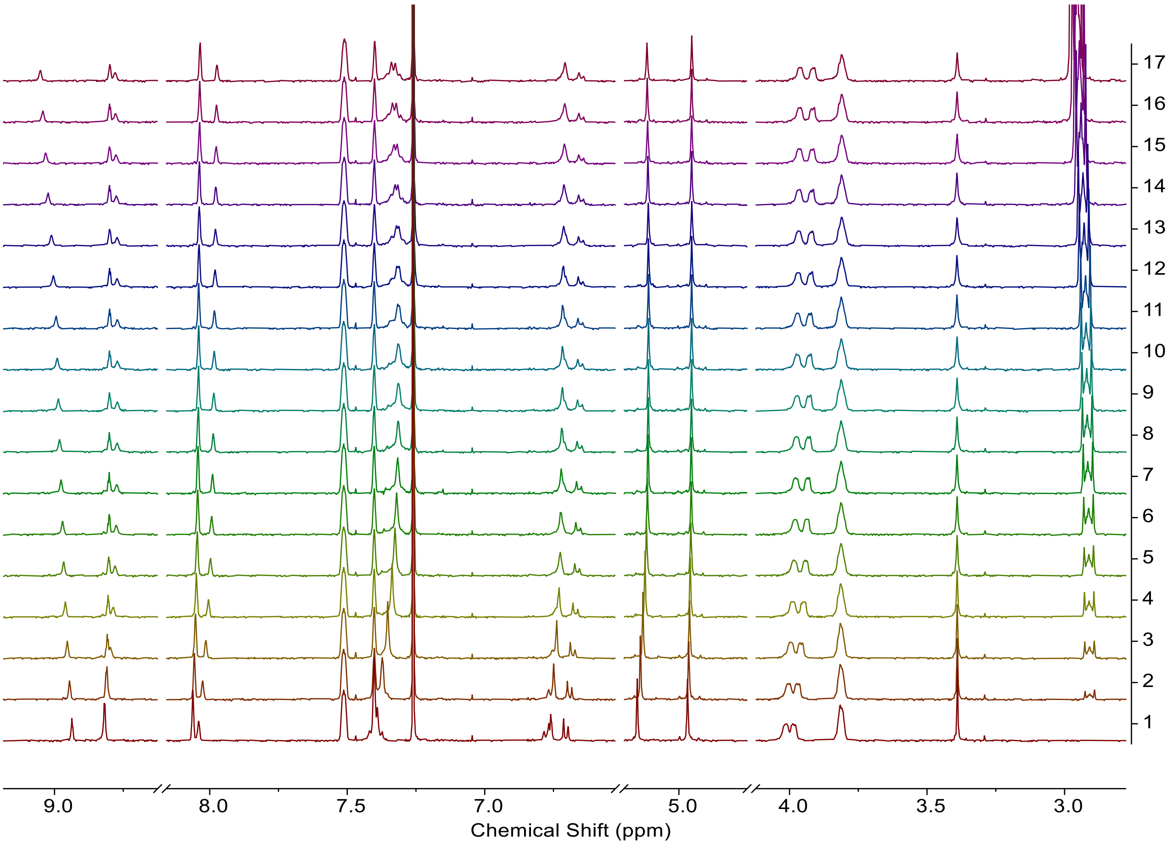

Figure S66. Truncated ^{1}H NMR titration spectra of **16** (0.5 mM) in presence of 1 equiv. KBArF (0.5 mM) with TBABr in $CDCl_3/CD_3CN$ (3:1 v/v), 500 MHz, 298 K. Spectrum no. 1 – 0 equivalents of the guest (TBABr); spectrum no. 17 – 10 equivalents of the guest (TBABr).

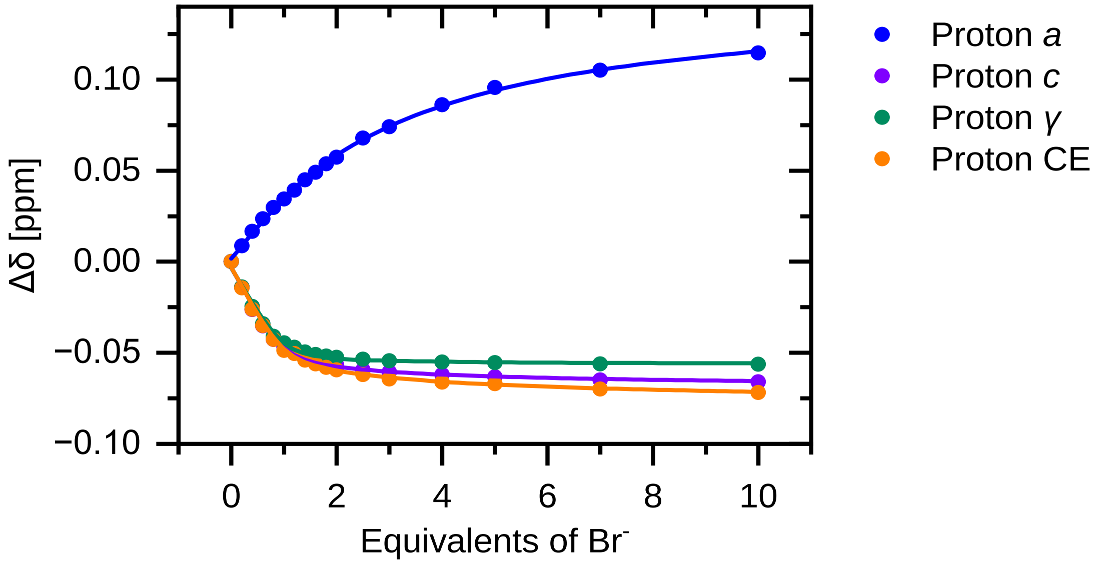

Figure S67. Examples of anion binding isotherms obtained during ^{1}H NMR titration of **1** (0.5 mM) in presence of 1 equiv. KBArF (0.5 mM) with TBABr in $CDCl_3/CD_3CN$ (3:1 v/v). Circles represent experimental data and the lines represent the fitted isotherms obtained using Bindfit (Model 1:2).

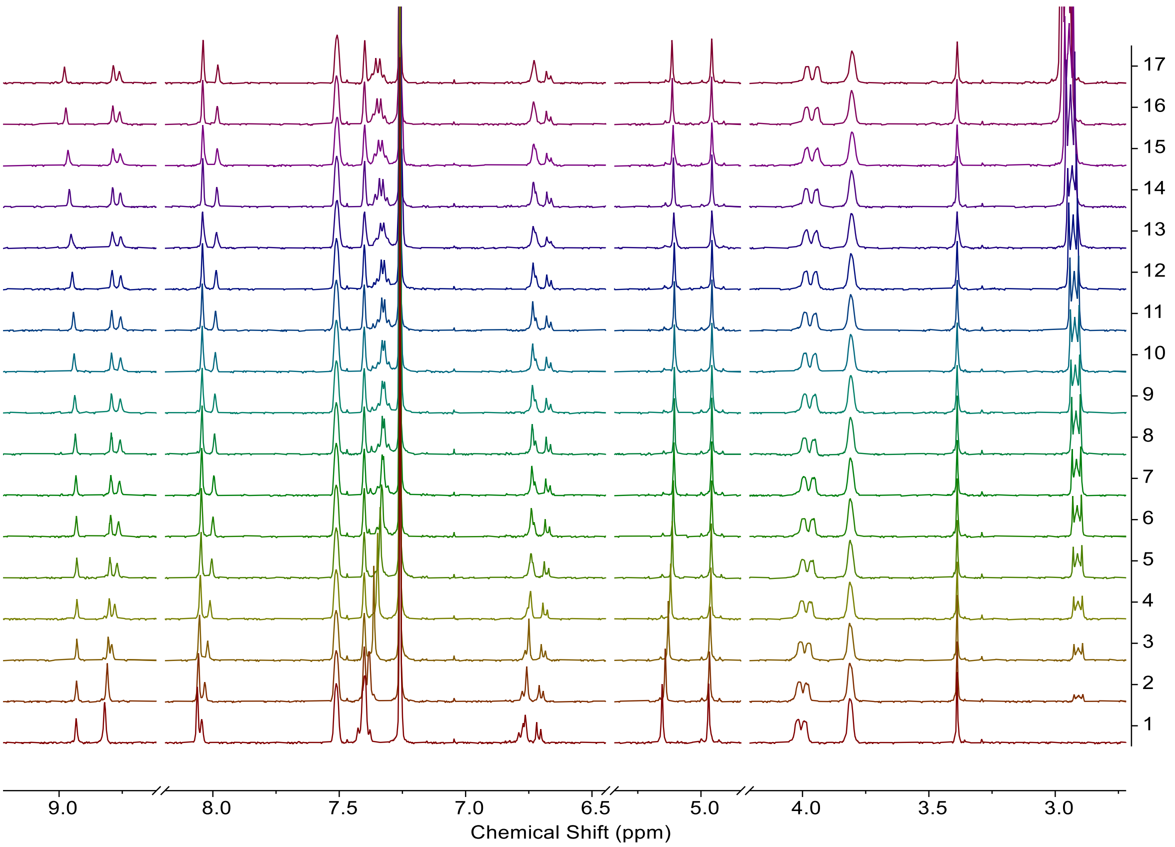

Figure S68. Truncated ^{1}H NMR titration spectra of **16** (0.5 mM) in presence of 1 equiv. KBArF (0.5 mM) with TBAI in $CDCl_3/CD_3CN$ (3:1 v/v), 500 MHz, 298 K. Spectrum no. 1 – 0 equivalents of the guest (TBAI); spectrum no. 17 – 10 equivalents of the guest (TBAI).

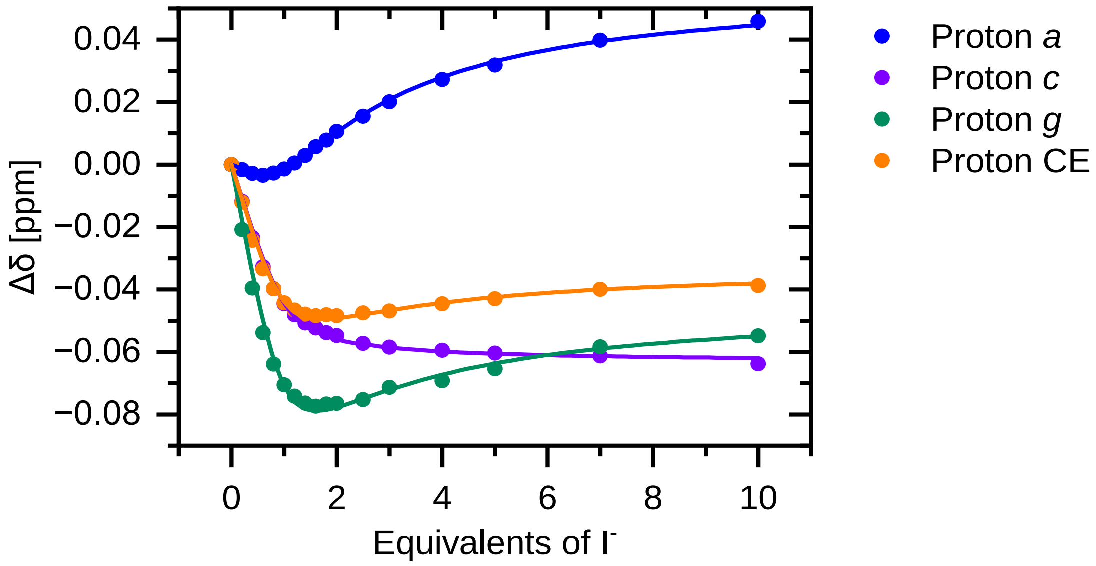

Figure S69. Examples of anion binding isotherms obtained during ^{1}H NMR titration of **1** (0.5 mM) in presence of 1 equiv. KBArF (0.5 mM) with TBABr in $CDCl_3/CD_3CN$ (3:1 v/v). Circles represent experimental data and the lines represent the fitted isotherms obtained using Bindfit (Model 1:2).

S3 Deconvolution of Binding Modes

S3.1 Two Binding Modes in 1:1 Model

Assume that receptor **R** forms two types of 1:1 complexes with guest **G**: (i) **RG** and (ii) **GR**. In this case the equilibrim can be described using the following equations:

$$(1)\ K^{RG} = \frac{[RG]}{[R][G]}$$
$$(2)\ K^{GR} = \frac{[GR]}{[R][G]}$$
$$(3)\ K^{1:1} = \frac{[RG]+[GR]}{[R][G]} = K^{RG} + K^{GR}$$

To simplify notation, parameter α is introduced, which describes a ratio of binding constant $K^{RG}$to overall binding constant $K^{1:1}$:

$$(4)\ \alpha = \frac{K^{RG}}{K^{1:1}}$$

Hence:

$$(5)\ K^{RG} = \alpha K^{1:1}$$
$$(6)\ K^{GR} = (1-\alpha)K^{1:1}$$

When this equilibrium is observed by ^{1}H NMR in a fast exchange regime, chemical shift of the receptor's observed signal, δ^{obs}, is equal to:

$$\delta^{obs} = \delta^{R}x^{R} + \delta^{RG}x^{RG} + \delta^{GR}x^{GR}$$

where δ^{i} is chemical shift of the signal in i-th species and x^{i} is a mole fraction of i-th species. Above equation can be rewritten in a simpler form:

$$1 = x^{R} + x^{RG} + x^{GR}$$
$$\delta^{obs} = \delta^{R} - \delta^{R}x^{RG} - \delta^{R}x^{GR} + \delta^{RG}x^{RG} + \delta^{GR}x^{GR}$$
$$\delta^{obs} - \delta^{R} = \left(\delta^{RG} - \delta^{R}\right)x^{RG} + \left(\delta^{GR} - \delta^{R}\right)x^{GR}$$

The chemical shift of the free receptor δ^{R} is a known parameter, therefore to simplify the notation, the above equation can be rewritten as:

$$\Delta\delta^{obs} = \delta_{\Delta}^{RG}x^{RG} + \delta_{\Delta}^{GR}x^{GR}$$

where δ_{Δ}^{i} describes the change of the chemical shift of the observed signal in i-th species. This equation can be rewritten using the definition of a mole fraction:

$$\Delta\delta^{obs} = \delta_{\Delta}^{RG}\frac{[RG]}{[R]_0} + \delta_{\Delta}^{GR}\frac{[GR]}{[R]_0}$$

Using formulas (1) and (2) yields:

$$\Delta\delta^{obs} = \frac{1}{[R]_0}\left(\delta_{\Delta}^{RG}K^{RG}[R][G] + \delta_{\Delta}^{GR}K^{GR}[R][G]\right)$$

By using formulas (5) and (6) the equation above can be rewritten as:

$$\Delta\delta^{obs} = \frac{[R][G]}{[R]_0} K^{1:1} \left(\delta_{\Delta}^{RG}\alpha + \delta_{\Delta}^{GR}(1-\alpha)\right)$$

By naming $\delta_{\Delta}^{RG}\alpha + \delta_{\Delta}^{GR}(1-\alpha) = \delta_{\Delta}^{1:1}$, the above equation takes a standard form, which is typically used for fitting isotherms with software packages like Bindfit, HypNMR, WinEQNMR, which assumes formation of one type of 1:1 complex only:

$$\Delta\delta^{obs} = \frac{[R][G]}{[R]_0} K^{1:1}\delta_{\Delta}^{1:1}$$

$\delta_{\Delta}^{1:1}$ is one of the fitted parameters (the other one is $K^{1:1}$). Importantly, it contains information about the ratio α and can be used to deconvolute binding modes:

$$\delta_{\Delta}^{1:1} = \delta_{\Delta}^{RG}\alpha + \delta_{\Delta}^{GR}(1-\alpha)$$

If formation of **GR** complex has no effect on the observed signal (e.g. binding sites are perfectly isolated), $\delta_{\Delta}^{GR} = 0$, the formula simplyfies to:

$$(7)\ \alpha = \frac{\delta_{\Delta}^{1:1}}{\delta_{\Delta}^{RG}}$$

To test this deconvolution approach using Bindfit, the artificial set of data was created using HySS software, which can simulate speciation.[9] The input values used to create the set of data are collected in Table S1.

Table S1. Binding parameters and values of chemical shifts used to simulate NMR titration of the system with two modes of 1:1 binding.

Species	K/M^{-1}	δ_1/ppm	δ_2/ppm
**RG**	6998.42	8.0	3.0
**GR**	2999.16	7.0	3.5
**R**	N/A	7.0	3.0
$[R]_0$	1 mM	$[G]_0$	0-10 mM
**No of points**		101	

The results obtained by 1:1 model fitting to the simulated data set using Bindfit are shown in Table S2. Binding constant obtained is equal to the sum of K^{RG} and K^{GR}. Values of α are equal to 0.7 and 0.3 for complexes **RG** and **RG**, respectively, which is the same as assumed in simulated data. Therefore, the suggested approach was successfully used to deconvolute binding modes after fitting 1:1 model using Bindfit.

Table S2. Results of isotherms fitting to 1:1 binding model using Bindfit for the simulated data of the system with two modes of 1:1 binding

	δ^{Host}	$\delta^{Complex}$	$\delta_{\Delta}^{1:1}$
**Proton 1**	7.0001	7.7000	0.70
**Proton 2**	3.0000	3.1500	0.15
**Binding constant**	9989.27 (± 0.1299%)		

S3.2 Two Binding Modes in 1:2 Model

The similar approach can be extended to higher models, for example 1:2 binding model, which assumes formation of 1:1 and 1:2 complexes. It allows for simple estimation of $\boldsymbol{\alpha}$ in 1:1 complexes. To test it, the artificial set of data was created, which models formation of two types of 1:1 complexes: **RG** & **GR** and two types of 1:1 complexes: $\mathbf{R_2G}$ & **GRG**. The input values used to create such a set are collected in Table S3.

Table S3. Binding parameters and values of chemical shifts used to simulate NMR titration of the system with two modes of 1:1 binding and two modes of 1:2 binding.

Species	K	δ_1	δ_2
$\mathbf{R_2G}$	489.7845	9.0	3.0
**GRG**	167.8823	8.0	3.5
**RG**	7000.032	8.0	3.0
**GR**	2999.853	7.0	3.5
**R**	N/A	7.0	3.0
$[R]_0$	1 mM	$[G]_0$	0-10 mM
**No of points**		101	

The results obtained by 1:2 model fitting using Bindfit are collected in Table S4. Again, $K^{1:1}$ is equal to the sum of $\boldsymbol{K^{RG}}$ and $\boldsymbol{K^{GR}}$, while $K^{1:2}$ is equal to $\boldsymbol{K^{GRG}}$ and $\boldsymbol{K^{G2R}}$. Values of $\boldsymbol{\alpha}$ for 1:1 complexes can be correctly calculated by equation (7).

Table S4. Results of isotherms fitting to 1:2 binding model using Bindfit for the simulated data of the system with two modes of 1:1 binding and two modes of 1:2 binding

	δ^{Host}	$\delta^{1:1}$	$\delta^{1:2}$
**Proton 1**	7.0001	7.6995	8.7445
**Proton 2**	3.0000	3.1499	3.1276
**$K^{1:1}$**	10022.32 (± 0.3152%)		
**$K^{1:2}$**	658.35 (± 0.00478%)		

S3.3 Analysis of Anion Binding by Receptors 1 and 2 in Presence of K^+

The approach presented above was used to deconvolute modes of 1:1 halide anions binding by receptors **1** and **2** in presence of 1 equiv. KBArF. Receptors **1**·K^+ and **2**·K^+ can bind anions in the anion binding domain by halogen bonding (mode **A**) and in the cation binding domain by forming the ion-pair with the crown ether bound K^+ (mode **B**). Based on the NMR titrations, it is assumed that this binding clefts are independent of each other. To find the contributions of each binding mode to the overall 1:1 binding constant, the chemical shift of one of the signal in 1:1 structure is needed. This value can be obtained from titrations of compound **9** in presence of KBArF. **9** possess only the cation binding domain and perturbations of its ^{1}H NMR signals during titrations with anions are analogues to the ones observed during titrations of **1** and **2**. The chemical shift values obtained from titration of **9**·K^+ provide good estimate of analogues values in **1**·K^+ and **2**·K^+. The values of $\delta_{\Delta}^{1:1}$ for the crown ether signal obtained from fitting are collected in Table S5. Equation (7) allows estimation of the ratio of binding mode **B** to the overall 1:1 binding for different halides.

Table S5. The change of the chemical shift (δ_{Δ}/ppm) of crown ether signal (CE) in a 1:1 complex obtained by fitting 1:2 binding isotherms using Bindfit to data from halides titrations of **9**, **1**, **2** in presence of 1 equiv. KBArF. Solvent $CDCl_3/CD_3CN$ 3:1 (v/v)

	**9·K^+**	**1·K^+**	**2·K^+**
**Cl**	0.455	0.168 (37%)[a]	0.197 (43%)[a]
**Br**	0.437	0.073 (17%)[a]	0.117 (27%)[a]
**I**	0.366	-[b]	-[b]

[a] Ratio of binding mode **B**, $\alpha = \frac{\delta_{\Delta}}{\delta_{\Delta}^{9}}$, to the overall 1:1 binding.
[b] Changes too small.

S4 Crystal Structure Determination

Table S6. Crystal data and selected structure refinement details for C_{60}-DB18C6 adduct **9** with a water molecule bound to crown ether.

CCDC Deposition Number	2282543
Identification code	DIA0441
Empirical formula	$C_{98}H_{42}O_{15}$
Formula weight	1459.40
Temperature	100 K
Wavelength	0.68890 Å
Crystal system	Monoclinic
Space Group	*C*2/*c*
Unit cell dimensions	*a* = 34.8991(4) Å<br>*b* = 10.12320(10) Å<br>*c* = 37.2234(4) Å<br>β = 93.7570(10)°
Volume	13122.4(2) Å^3
Z	8
Density (calculated)	1.4777 Mg/m^3
Absorption coefficient	0.092 mm^{-1}
F(000)	6000.007
Crystal size	0.230 × 0.075 × 0.015 mm^3
Theta range for data collection	1.063 to 35.820°
Index ranges	$-57 \leq h \leq 59$<br>$-17 \leq k \leq 17$<br>$-61 \leq l \leq 61$
Reflections collected	132092
Independent reflections	31219 [R_{int} = 0.046]
Completeness to theta = 33.825°	99.3%
Absorption correction	Empirical
Max and min transmission	1.0 and 0.972
Refinement method	Full-matrix least-squares on F^2
Data / restrains / parameters	27307 / 162 / 1068
Goodness-of-fit on F^2	0.9959
Final R indices [I>2σ(I)]	R_1 = 0.0767, wR_2 = 0.0694
R indices (all data)	R_1 = 0.1135, wR_2 = 0.0709
Largest diff. peak and hole	2.75 and -1.36 e.Å^{-3}

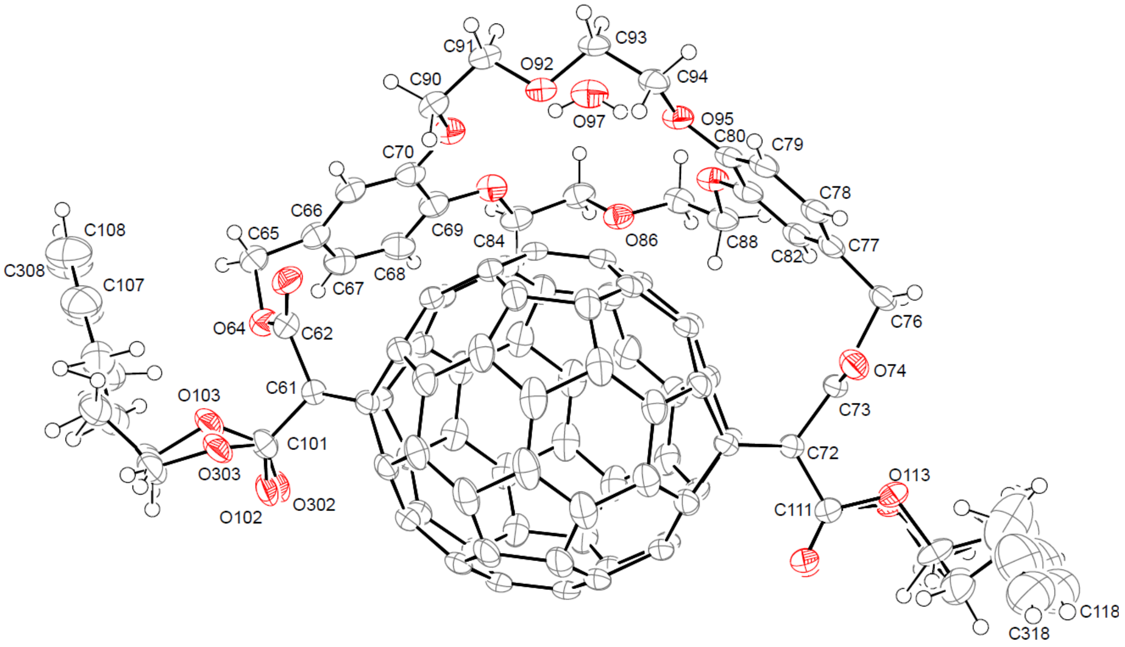

Figure S70. ORTEP view of **9** with thermal ellipsoids drawn at the 50% of probability, showing a water molecule within the crown-ether moiety of the adduct.

S5 Computational Analysis

S5.1 Methods

1 and **2** were generated by atomic manipulation of single crystal X-ray diffraction structures, deposited with the Cambridge Crystallographic Data Centre (CCDC). The C_{60}-dibenzo-18-crown-6 adduct was obtained from the crystal structure with the CSD RefCode DEXSOO,[1] while the biphenyl-1,3-bis-(iodotriazole)nitroaryl moiety was taken from the crystal structure with the CSD RefCode WOMZUW.[10] C_{60} and **9** were obtained from the solid-state structure of **9** with a water molecule bound to the crown ether.

Conformational searches for the different binding modes of **1** and **2** with K^+ and Cl^- were obtained through molecular dynamics (MD) simulations carried out in the gas phase using AMBER20.[11] The receptors were described using the updated version of the General AMBER Force Field (GAFF2),[12] together with ESP atomic charges. K^+ and Cl^- were described with net charges of +1 or -1 and suitable van der Waals parameters.[13] At this coarse level, halogen bonding interactions were preserved with distance and angle restraints appropriate for each binding scenario. The initial binding arrangements were energy minimised by molecular mechanics (MM), followed by a heating step to 500 K for 50 ps, and ensued by a 1 ns collection run, using a 1 fs time step. Each run resulted in 10000 structures, which were saved, and further energy minimised by MM until the convergence criterion of 0.0001 kcal mol^{-1} was reached. This process was repeated several times, leading to multiple independent MD runs for each binding mode. Afterwards, the binding arrangements selected for DFT optimisations, presented in the main text and herein, were chosen from an RMSD clustering analysis of the MM-minimised frames.

The Natural Population Analysis charges and Second Order Perturbation Theory energies were determined by Natural Bond Orbital analysis using NBO 7.[14,15]

S5.2 Additional Figures

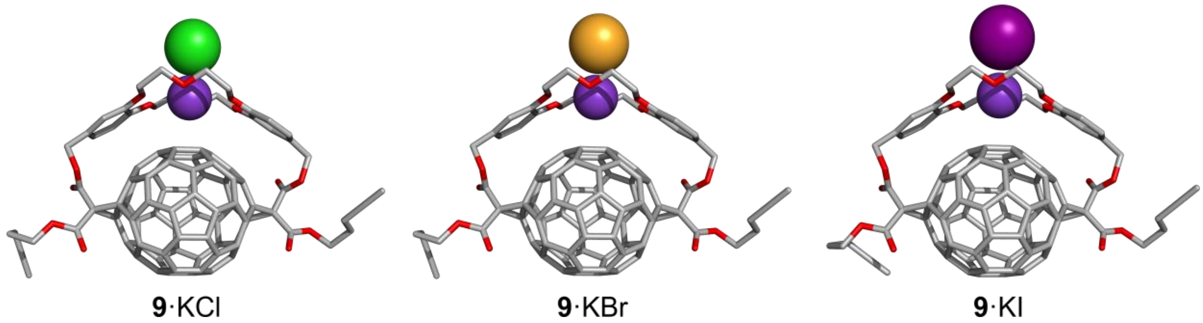

Figure S71. Structures of the **9**·KX complexes, optimized in gas-phase with the M06-2X functional and the Def2-SVP basis set (H, C, O and K^+), together with the Def2-TZVPD basis set for the halides.

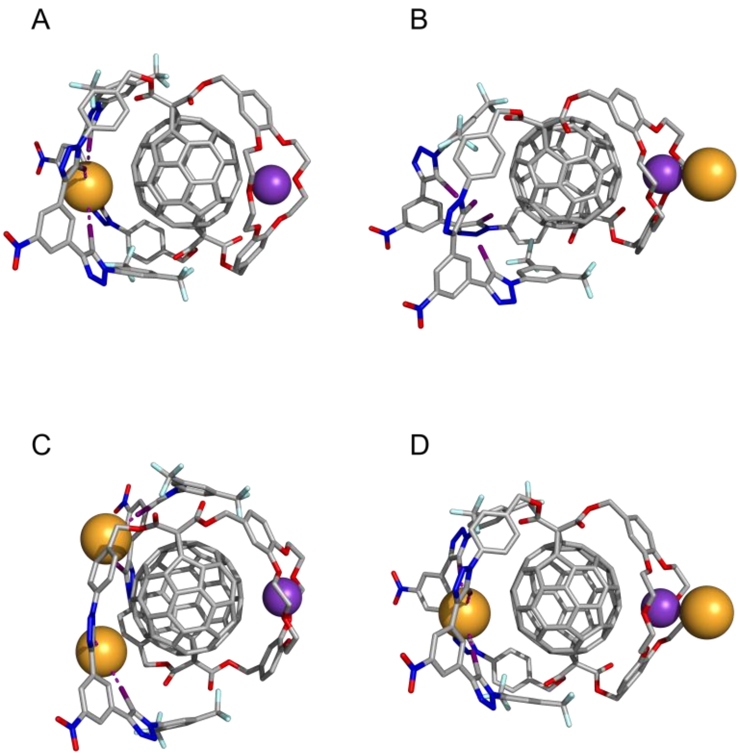

Figure S72. DFT optimized binding scenarios **A**-**D** for the bromide complexes of **2**.

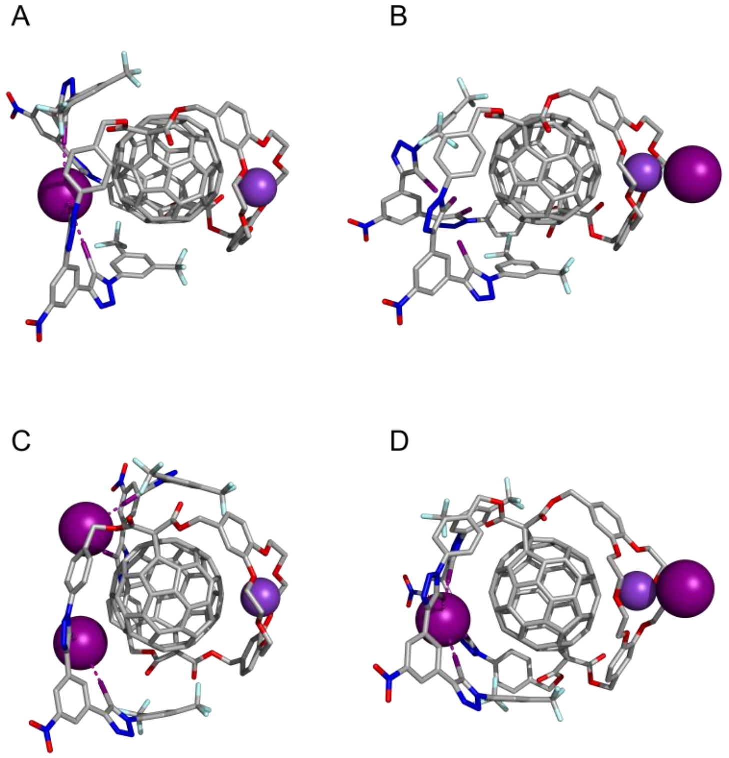

Figure S73. DFT optimized binding scenarios **A-D** for the iodide complexes of **2**.

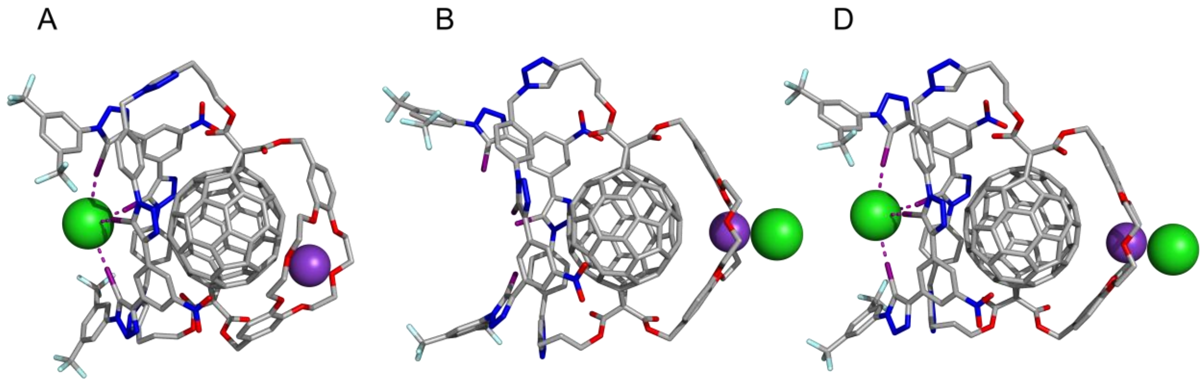

Figure S74. DFT optimized binding scenarios **A**, **B**, and **D** for the chloride complexes of **1**.

S5.3 Additional Tables

Table S7. Selected distances (Å) in halide complexes of **2** in different binding modes.[a]

Binding scenario	A	B	C[b]	D
$C_6/C_5\cdots Cl^-$	3.13	–	3.41/3.22	3.17
$K^+\cdots Cl^-$	11.96	2.83	12.02; 12.12	12.29; 2.83
$K^+\cdots C_6$	3.04	3.53	2.90	3.29
$K^+\cdots O$	2.66 - 2.79	2.74 - 2.76	2.69 - 2.77	2.72 - 2.77
$C_6/C_5\cdots Br^-$	3.35	–	3.50/3.40	3.37
$K^+\cdots Br^-$	12.19	3.00	12.13; 12.31	12.52; 3.02
$K^+\cdots C_6$	2.99	3.54	2.90	3.26
$K^+\cdots O$	2.66 - 2.79	2.74 - 2.76	2.69 - 2.78	2.72 - 2.77
$C_6/C_5\cdots I^-$	3.63	–	3.61/3.68	3.64
$K^+\cdots I^-$	12.50	3.21	12.30; 12.53	12.85; 3.24
$K^+\cdots C_6$	3.00	3.57	2.89	3.27
$K^+\cdots O$	2.66 - 2.79	2.74 - 2.77	2.70 - 2.78	2.72 - 2.78

[a)] C_6 or C_5 represents the centroid of the closest six- or five-membered ring in the C_{60} scaffold; $K^+\cdots O$ is the range of distances between potassium and the oxygen atoms of the crown-ether. [b)] $K^+\cdots X^-$ (X^- = Cl^-, Br^-, or I^-) in the C binding mode means the distances between potassium and the halides closest of the C_6 and C_5 rings, given in this order.

Table S8. Computed distances (Å) and angles (°) of the XB interactions between **2** and halides in different binding modes. Entries and values in blue correspond to the XB interactions activated by the $-CF_3$ groups.

Binding scenario	A		C		D	
$C-I_1\cdots Cl^-$	3.010	174.1	2.919	172.9	3.012	174.4
$C-I_2\cdots Cl^-$	3.371	163.2	3.259	166.2	3.369	162.8
$C-I_3\cdots Cl^-$	3.046	172.1	2.989	171.2	3.028	172.2
$C-I_4\cdots Cl^-$	3.328	162.3	3.256	165.3	3.322	161.9
$C-I_1\cdots Br^-$	3.182	175.9	3.096	174.5	3.180	175.8
$C-I_2\cdots Br^-$	3.585	162.4	3.502	166.4	3.586	161.3
$C-I_3\cdots Br^-$	3.232	173.0	3.156	171.7	3.212	173.2
$C-I_4\cdots Br^-$	3.568	160.8	3.373	167.5	3.559	160.3
$C-I_1\cdots I^-$	3.405	177.3	3.305	176.4	3.395	177.1
$C-I_2\cdots I^-$	3.795	164.5	3.759	166.4	3.831	161.1
$C-I_3\cdots I^-$	3.456	173.8	3.354	172.9	3.434	174.6
$C-I_4\cdots I^-$	3.872	159.6	3.549	169.3	3.827	159.8

Table S9. Second Order perturbation energies (E^2, kcal mol^{-1}) assessed for the XB interactions between **2** and halides in different binding modes. Entries and values in blue correspond to the XB interactions activated by the -CF_3 groups.

Binding scenario	A	C	D
C-I_1···Cl^-	14.5	24.7	14.2
C-I_2···Cl^-	5.1	9.0	5.0
C-I_3···Cl^-	12.0	19.0	12.9
C-I_4···Cl^-	5.9	8.7	5.9
Total	37.5	61.3	38.0
C-I_1···Br^-	14.6	22.6	14.5
C-I_2···Br^-	4.6	6.9	4.5
C-I_3···Br^-	12.2	17.8	13.0
C-I_4···Br^-	4.8	9.4	4.9
Total	36.3	56.7	36.8
C-I_1···I^-	13.3	20.7	13.6
C-I_2···I^-	4.8	5.7	4.2
C-I_3···I^-	11.3	16.8	12.1
C-I_4···I^-	3.7	9.5	4.2
Total	33.1	52.6	34.0

S6 References

(1) Bourgeois, J.-P.; Seiler, P.; Fibbioli, M.; Pretsch, E.; Diederich, F.; Echegoyen, L. Cyclophane-Type Fullerene-dibenzo[18]Crown-6 Conjugates with Trans-1, Trans-2, and Trans-3 Addition Patterns: Regioselective Templated Synthesis, X-Ray Crystal Structure, Ionophoric Properties, and Cation-Complexation-Dependent Redox Behavior. *Helv. Chim. Acta* **1999**, *82*, 1572–1595.

(2) Hilmer, A. J.; Tvrdy, K.; Zhang, J.; Strano, M. S. Charge Transfer Structure–Reactivity Dependence of Fullerene–Single-Walled Carbon Nanotube Heterojunctions. *J. Am. Chem. Soc.* **2013**, *135*, 11901–11910.

(3) Iehl, J.; Osinska, I.; Louis, R.; Holler, M.; Nierengarten, J.-F. A Stable Fullerene-Azide Building Block for the Construction of a Fullerene–Porphyrin Conjugate. *Tetrahedron Lett.* **2009**, *50*, 2245–2248.

(4) Bickerton, L. E.; Docker, A.; Sterling, A. J.; Kuhn, H.; Duarte, F.; Beer, P. D.; Langton, M. J. Highly Active Halogen Bonding and Chalcogen Bonding Chloride Transporters with Non-Protonophoric Activity. *Chem. Eur. J.* **2021**, *27*, 11738–11745.

(5) Sebest, F.; Casarrubios, L.; Rzepa, H. S.; White, A. J. P.; Díez-González, S. Thermal Azide–Alkene Cycloaddition Reactions: Straightforward Multi-Gram Access to Δ^2-1,2,3-Triazolines in Deep Eutectic Solvents. *Green Chem.* **2018**, *20*, 4023–4035.

(6) Hong, L.; Lin, W.; Zhang, F.; Liu, R.; Zhou, X. Ln[N(SiMe3)2]3-Catalyzed Cycloaddition of Terminal Alkynes to Azides Leading to 1,5-Disubstituted 1,2,3-Triazoles: New Mechanistic Features. *Chem. Commun.* **2013**, *49*, 5589-5591.

(7) Brynn Hibbert, D.; Thordarson, P. The Death of the Job Plot, Transparency, Open Science and Online Tools, Uncertainty Estimation Methods and Other Developments in Supramolecular Chemistry Data Analysis. *Chem. Commun.* **2016**, *52*, 12792–12805.

(8) http://supramolecular.org.

(9) Alderighi, L.; Gans, P.; Ienco, A.; Peters, D.; Sabatini, A.; Vacca, A. Hyperquad Simulation and Speciation (HySS): A Utility Program for the Investigation of Equilibria Involving Soluble and Partially Soluble Species. *Coord. Chem. Rev.* **1999**, *184*, 311–318.

(10) Bunchuay, T.; Docker, A.; Martinez-Martinez, A. J.; Beer, P. D. A Potent Halogen-Bonding Donor Motif for Anion Recognition and Anion Template Mechanical Bond Synthesis. *Angew. Chem. Int. Ed.* **2019**, *58*, 13823–13827.

(11) Case, D. A.; Belfon, K.; Ben-Shalom, I. Y.; Brozell, S. R.; Cerutti, D. S.; Cheatham, T. E., III; Cruzeiro, V. W. D.; Darden, T. A.; Duke, R. E.; Giambasu, G.; Gilson, M. K.; Gohlke, H.; Goetz, A. W.; Harris, R.; Izadi, S.; Izmailov, S. A.; Kasavajhala, K.; Kovalenko, A.; Krasny, R.; Kurtzman, T.; Lee, T. S.; LeGrand, S.; Li, P.; Lin, C.; Liu, J.; Luchko, T.; Luo, R.; Man, V.; Merz, K. M.; Miao, Y.; Mikhailovskii, O.; Monard, G.; Nguyen, H.; Onufriev, A.; Pan, F.; Pantano, S.; Qi, R.; Roe, D. R.; Roitberg, A.; Sagui, C.; Schott-Verdugo, S.; Shen, J.; Simmerling, C. L.; Skrynnikov, N. R.; Smith, J.; Swails, J.; Walker, R. C.; Wang, J.; Wilson, L.; Wolf, R. M.; Wu, X.; Xiong, Y.; Xue, Y.; York, D. M.; Kollman, P. A. AMBER 2020. University of California, San Francisco. **2020**.

(12) Wang, J.; Wolf, R. M.; Caldwell, J. W.; Kollman, P. A.; Case, D. A. Development and Testing of a General Amber Force Field. *J. Comput. Chem.* **2004**, *25*, 1157–1174.

(13) Li, P.; Song, L. F.; Merz, K. M. Systematic Parameterization of Monovalent Ions Employing the Nonbonded Model. *J. Chem. Theory Comput.* **2015**, *11*, 1645–1657.

(14) Weinhold, F. Natural Bond Orbital Analysis: A Critical Overview of Relationships to Alternative Bonding Perspectives. *J. Comput. Chem.* **2012**, *33*, 2363–2379.

(15) Glendening, J. E. D.; Badenhoop, K.; Reed, A. E.; Carpenter, J. E.; Bohmann, J. A.; Morales, C. M.; Karafiloglou, P.; Landis, C. R.; Weinhold, F. NBO 7.0. Theoretical Chemistry Institute, University of Wisconsin, Madison. **2018**.
